# A framework for individualized splice-switching oligonucleotide therapy

**DOI:** 10.1038/s41586-023-06277-0

**Published:** 2023-07-12

**Authors:** Jinkuk Kim, Sijae Woo, Claudio M. de Gusmao, Boxun Zhao, Diana H. Chin, Renata L. DiDonato, Minh A. Nguyen, Tojo Nakayama, Chunguang April Hu, Aubrie Soucy, Ashley Kuniholm, Jennifer Karlin Thornton, Olivia Riccardi, Danielle A. Friedman, Christelle Moufawad El Achkar, Zane Dash, Laura Cornelissen, Carolina Donado, Kamli N. W. Faour, Lynn W. Bush, Victoria Suslovitch, Claudia Lentucci, Peter J. Park, Eunjung Alice Lee, Al Patterson, Anthony A. Philippakis, Brad Margus, Charles B. Berde, Timothy W. Yu

**Affiliations:** 1 Graduate School of Medical Science and Engineering, Korea Advanced Institute of Science and Technology (KAIST), Daejeon, Republic of Korea; 2 Biomedical Research Center, Korea Advanced Institute of Science and Technology (KAIST), Daejeon, Republic of Korea; 3 KI for Health Science and Technology, Korea Advanced Institute of Science and Technology (KAIST), Daejeon, Republic of Korea; 4 Center for Epidemic Preparedness, Korea Advanced Institute of Science and Technology (KAIST), Daejeon, Republic of Korea; 5 Department of Neurology, Boston Children’s Hospital, Boston, MA USA; 6 Postgraduate School of Medical Science, University of Campinas (UNICAMP), São Paulo, Brazil; 7 Division of Genetics and Genomics, Boston Children’s Hospital, Boston, MA USA; 8 Manton Center for Orphan Disease Research, Boston Children’s Hospital, Boston, MA USA; 9 Department of Pediatrics, Boston Children’s Hospital, Boston, MA USA; 10 Program in Medical and Population Genetics, Broad Institute of MIT and Harvard, Cambridge, MA USA; 11 Harvard Medical School, Boston, MA USA; 12 Institutional Center for Clinical and Translational Research, Boston Children’s Hospital, Boston, MA USA; 13 Ataxia Telangiectasia Children’s Project, Coconut Creek, FL USA; 14 Department of Anesthesiology, Critical Care and Pain Medicine, Boston Children’s Hospital, Boston, MA USA; 15 Center for Bioethics, Harvard Medical School, Boston, MA USA; 16 Department of Biomedical Informatics, Harvard Medical School, Boston, MA USA; 17 Department of Pharmacy, Boston Children’s Hospital, Boston, MA USA; 18 Eric and Wendy Schmidt Center, Broad Institute of MIT and Harvard, Cambridge, MA USA

**Keywords:** Paediatric neurological disorders, Drug development, Translational research, Paediatric research

## Abstract

Splice-switching antisense oligonucleotides (ASOs) could be used to treat a subset of individuals with genetic diseases^1^, but the systematic identification of such individuals remains a challenge. Here we performed whole-genome sequencing analyses to characterize genetic variation in 235 individuals (from 209 families) with ataxia-telangiectasia, a severely debilitating and life-threatening recessive genetic disorder^2,3^, yielding a complete molecular diagnosis in almost all individuals. We developed a predictive taxonomy to assess the amenability of each individual to splice-switching ASO intervention; 9% and 6% of the individuals had variants that were ‘probably’ or ‘possibly’ amenable to ASO splice modulation, respectively. Most amenable variants were in deep intronic regions that are inaccessible to exon-targeted sequencing. We developed ASOs that successfully rescued mis-splicing and ATM cellular signalling in patient fibroblasts for two recurrent variants. In a pilot clinical study, one of these ASOs was used to treat a child who had been diagnosed with ataxia-telangiectasia soon after birth, and showed good tolerability without serious adverse events for three years. Our study provides a framework for the prospective identification of individuals with genetic diseases who might benefit from a therapeutic approach involving splice-switching ASOs.

## Abstract (web summary)

Whole-genome sequencing analyses in a cohort of individuals with ataxia-telangiectasia are used to identify genetic variants that might be amenable to treatment with splice-switching antisense oligonucleotides (ASOs), and develop ASOs with therapeutic potential.

## Main

It is estimated that 1 in 10 individuals—more than 30 million people in the USA—have a diagnosis of a rare disease^4^. Although this term encompasses an estimated 7,000 different conditions, with a notable proportion having a known or presumed genetic aetiology, treatments are available for only around 5% of them^4,5^. The rarity of these conditions often makes it economically infeasible to develop treatments based on traditional modalities. Previous work has shown that, in some cases, splice-switching ASOs may restore functional protein levels and be administered in a safe and timely manner^1^. Such therapy presents a treatment opportunity; however, identifying individuals with genetic variants suitable for such attempts remains a challenge^1,6^. Here, we present a framework to systematically discover and develop splice-switching treatments for individuals with rare diseases, using ataxia-telangiectasia (A-T) as a model.

A-T is an autosomal recessive disease caused by biallelic loss of function of *ATM*, a gene involved in the cellular response to DNA double-strand breaks^7^. Clinically, A-T is characterized by progressive cerebellar degeneration, immunodeficiency and predisposition to cancer, with early manifestations including ataxia, involuntary movements, neuropathy, oculomotor apraxia, dysphagia, slurred speech and ocular and skin telangiectasias^3^. The disease has a prevalence of 1 in 40,000 to 100,000 live births worldwide^2^. In ‘classical’ A-T (the most typical and prevalent clinical presentation), the average life expectancy is 25 years, and early death is most frequently due to lung disease or cancer^2^. Hypomorphic *ATM* variants can cause a milder, slower-progressing phenotype, albeit still with severe morbidity^8^.

A-T is the most common form of inherited childhood progressive ataxia in many countries^9^. Nevertheless, effective therapies are lacking—particularly for the profound neurological manifestations that have a marked effect on quality of life. The large coding size (9.2 kb) of *ATM* poses a challenge for gene therapy approaches, as AAV vectors that are at present available have a packaging capacity of around 4.7 kb (ref. ^10^). The protein product of *ATM* is an intracellular kinase, which makes enzyme replacement approaches difficult. By contrast, A-T could be an appealing candidate for splice-switching ASO therapy, given that ASOs readily distribute to the cerebellum when injected intrathecally^11^.

The Global A-T Family Data Platform is an international initiative led by family advocates and primarily funded by A-T Children’s Project (ATCP), a patient-advocacy foundation for A-T. ATCP has systematically collected clinical information and genomic DNA (gDNA) from individuals who have been clinically or genetically diagnosed with A-T. Through this effort, whole-genome sequencing (WGS) was performed, yielding a WGS dataset of 235 individuals with A-T. We analysed this ‘ATCP cohort’ to establish molecular diagnoses, identify pathogenic variants that could be amenable to splice-switching ASO therapy, develop proof-of-concept individualized ASOs and initiate a pilot investigational trial.

## Patients and diagnostic yield

The ATCP cohort comprised de-identified phenotypic and genomic data from 235 individuals who were clinically or genetically diagnosed with A-T, from 209 families. Of these individuals, 46% were female (108/235) and the median age of clinical A-T diagnosis was 3.6 years (range 0–32; Extended Data Table 1 and Supplementary Table 1).

**Fig. 1:**
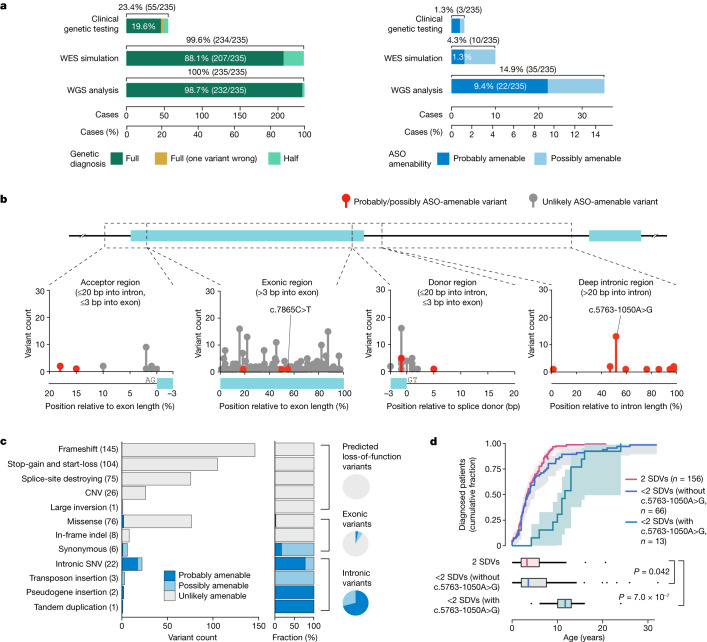
*ATM* disease candidate variants. **a**, Diagnostic (left; green) and ASO therapeutic (right; blue) yields of clinical genetic testing, WES simulation and WGS analysis for the 235 patients in the ATCP cohort. The WES simulation assumed complete recovery of all exonic variants ± 20 bp. **b**, Positions of *ATM* disease candidate variants. All SNVs and short indels, as well as SVs that are fully contained in a single intron, are depicted. Two variants for which a splice-switching ASO was developed (c.7865C>T and c.5763-1050A>G) in this study are indicated. Deletion variants that span a splice junction were considered to be located at the junction (position 0). Short indels and SVs were considered to be located at the boundary of the variant closest to a nearby splice junction. **c**, Types and ASO amenability of all disease candidate variants present in the cohort. **d**, Cumulative fraction and box plot distribution of the age at diagnosis, based on the possession of SDVs and a known hypomorphic variant (c.5763-1050A>G). The *P* values (shown in the figure) were calculated through the log-rank test using the survminer R package, and were adjusted by the Bonferroni correction. Shaded areas, fraction ± 95% confidence interval. For the box plots: centre, median; lower hinge, 25th percentile; upper hinge, 75th percentile; lower whisker, smallest value greater than or equal to lower hinge − 1.5 × interquartile range (IQR); upper whisker, largest value less than or equal to upper hinge + 1.5 × IQR.

The results of molecular genetic testing were available for only a small subset of individuals within the cohort (23%, 55/235), mostly from sequencing of *ATM* coding regions (Fig. 1a and Extended Data Table 1). We therefore performed an extensive set of WGS analyses on all individuals to generate a comprehensive picture of genetic variation in A-T. We identified single-nucleotide variants (SNVs) and short indels using GATK^12^ (Supplementary Table 2), VarScan2^13^ and Strelka2^14^; and copy number variants (CNVs), structural variants (SVs) and mobile DNA element insertions using Delly^15^, Pindel^16^, MELT^17^ and xTea^18^ (Supplementary Table 3). For completeness, we manually inspected sequencing reads across the *ATM* locus using the Integrative Genomics Viewer to identify variants that might have been missed by the aforementioned methods. Variants were named with respect to the GRCh38/hg38 reference genome and NM_000051.3, the canonical RefSeq transcript for *ATM*.

We assessed the pathogenicity of variants using coding and splice impact predictions, population allele frequency (gnomAD and TOPMed) and experimental evidence (Supplementary Table 1; for details, see Methods). Variants that we considered to be disease-causing were referred to as ‘disease candidate’ variants. We noted that some of the disease candidate variants had been formally classified as variants of uncertain significance (VUSs) by American College of Medical Genetics and Genomics (ACMG) guidelines^19^. These were considered disease-causing for the purpose of this analysis if (1) there was strong support from computational predictions as disrupting gene function (for example, SpliceAI^20^ or REVEL^21^ for splicing and coding impact predictions, respectively); and (2) no other variants were identified by WGS that could account for the clinical diagnosis (Supplementary Note 1). Similarly, some coding CNVs were considered disease candidate variants even when officially classified as VUSs by ACMG guidelines. Use of such a broader definition of pathogenicity is not unprecedented in WGS studies^22^, in which a near-complete landscape of mutational events for a given individual can be characterized. Such VUSs constituted a relatively small proportion of the disease candidate variants (22 of 220 unique variants; 27 of 469 total mutational events).

**Extended Data Fig. 1:**
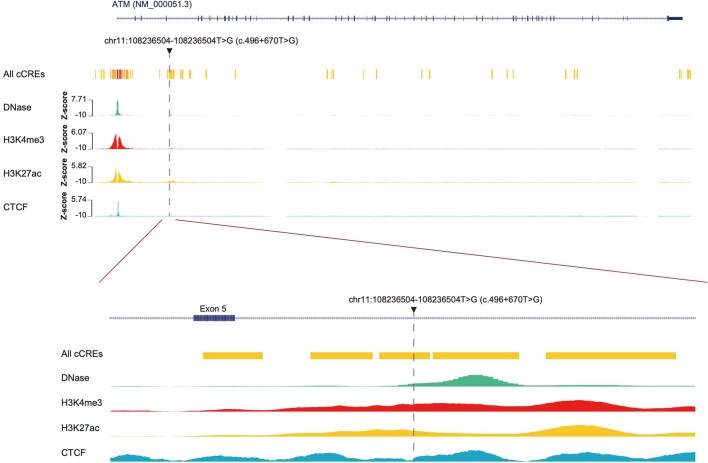
A VUS in a potential *ATM* enhancer. The *ATM* c.496+670T>G (chr11:108236504-108236504T>G) variant, identified in DDP_ATCP_368, is located in a distal enhancer-like element, as catalogued by ENCODE. The absence of this variant in both gnomAD v3.1 and TOPMed freeze 8, with an allele frequency of 0.0, is consistent with a potential pathogenic role. Declaring this variant as disease-causing could complete the genetic diagnosis for DDP_ATCP_368. However, owing to a lack of substantial evidence confirming that the enhancer-like element is functional, and that the variant disrupts its function, we have refrained from declaring this variant as a disease candidate. For more details, see Supplementary Note 2.

At least 2 disease candidate *ATM* variants were identified in 232 out of 235 individuals (98.7%; Fig. 1a and Supplementary Table 1). Two of the other three individuals had one disease candidate variant and a VUS that we considered to have plausible biological impact, but were not confident enough to classify as a disease candidate variant (Extended Data Fig. 1 and Supplementary Note 2). In the remaining case, only one heterozygous disease candidate variant was found (that is, a second pathogenic event was not found). We also noted that in 3 out of 235 cases, our WGS analyses resulted in a revision of previously reported genetic testing results (Supplementary Note 3).

To confirm the fidelity of our variant calls, gDNA samples were obtained from 16 patients. Sanger sequencing validated all tested variant calls in these samples (31/31 mutational events; Supplementary Tables 4 and 5). We were also able to use experimental or in silico approaches to analyse variant phasing in 88 patients (from 78 families): (1) trio Sanger sequencing was performed for 5 cases for which gDNA samples were available (Supplementary Tables 4 and 6); (2) for two cases, read-based phasing was performed by using WhatsHap^23^; (3) for 47 cases, analyses of variant co-occurrence in gnomAD strongly predicted that the 2 observed variants were in *trans*; (4) 32 cases involved homozygous *ATM* variants; and (5) for 5 cases, a variant appeared homozygous because it was in *trans* to a deletion. In aggregate, these analyses confirmed that 88 cases (from 78 families) have disease candidate variants on both alleles (Supplementary Table 1; for more details on phasing, see Methods).

We simulated the diagnostic yield of an exon-targeted sequencing approach if performed in lieu of WGS. Perfect coverage of all exonic variants ± 20 nucleotides from flanking regions would have established a molecular diagnosis in 207 of 235 individuals (a diagnostic yield of 88.1%, compared to 98.7% achieved with comprehensive WGS analyses; Fig. 1a,b).

## Disease candidate variants

We identified 469 disease candidate *ATM* mutational events in 235 patients. These comprised a total of 220 unique *ATM* variants, ranging from SNVs to short indels and SVs. A substantial fraction of these events (75%, 353/469) were predicted to confer strong loss of function, including frameshift variants (31%, 145/469), stop-gain or start-loss variants (22%, 104/469), splice-site-destroying variants (16%, 75/469; defined on the basis of SpliceAI^20^ and MaxEntScan^24^ predictions; for details, see Methods) and missense variants or short in-frame indels with previous experimental evidence of complete loss of ATM kinase activity (29/469, 6.2%; Fig. 1c and Supplementary Table 7).

**Extended Data Fig. 2:**
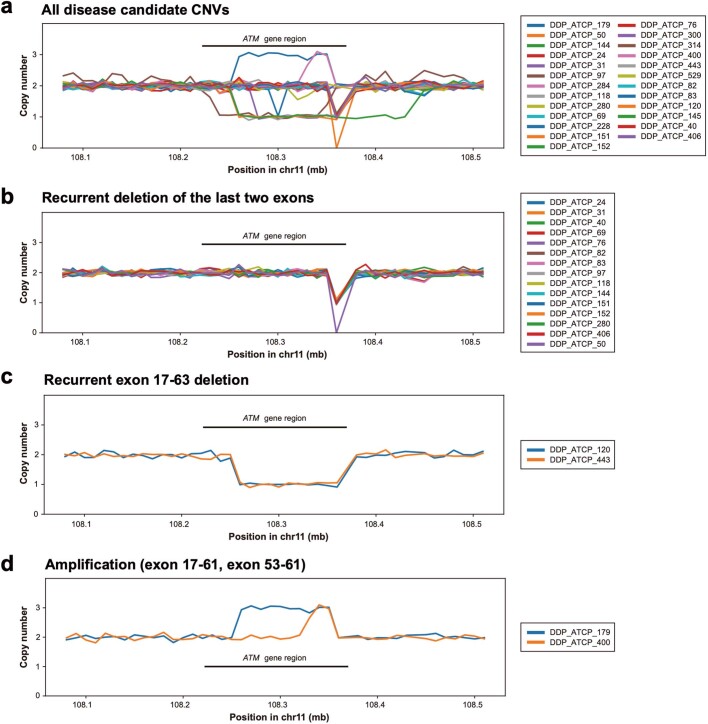
Disease candidate CNVs. **a**, All disease candidate CNVs (26 mutational events, 25 patients including one homozygote, 10 unique variants; Supplementary Table 3). **b**, A recurrent (16 mutational events, 15 patients including one homozygote) CNV event, which deletes the last two *ATM* exons (exon 62 and 63). The deletion is found in 15 patients (DDP_ATCP_50 is homozygous). **c**, A recurrent (2 mutational events, 2 patients) CNV event, which deletes exon 17 through 63. Precise breakpoints of these two deletions are yet to be pinpointed. **d**, Two CNV events that duplicate a portion of the *ATM* gene, exon 17–61 and exon 53–61.

SVs that severely disrupt the open reading frame of *ATM* (out-of-frame inversions or deletions; inversions or deletions involving the start or stop codon; or duplication of more than 10 middle coding exons) made up 5.8% (27/469) of all disease candidate mutational events (Supplementary Table 3). These included 26 copy number changes (23 deletions, 2 duplications and 1 complex rearrangement (combined inversion and deletion); Extended Data Fig. 2 and Supplementary Fig. 2) and 1 copy number-neutral inversion event (involving the first 16 exons of *ATM*; Supplementary Fig. 3). Of the 23 deletion events, 16 involved the last 2 exons of *ATM* (a region that includes the stop codon and partially encodes the ATM kinase domain), present in 15 patients (1 patient was homozygous for the deletion, hence 16 events in 15 patients).

The 27 SVs disrupting the open reading frame, together with the 353 additional strong loss-of-function mutational events mentioned above (frameshift, stop-gain, start-loss, splice-site-destroying or missense variants with previous experimental evidence of loss of function), are expected to lead to deficient and/or absent ATM protein expression or function (*ATM* null). This group, which we refer to as ‘severely disruptive variants’ (SDVs), constitutes 81% (380/469) of the disease candidate mutational events encountered. More than half of the individuals in this cohort (66%, 156/235) had two of these SDV events, which would be expected to lead to the classical A-T phenotype with a more severe prognosis. Indeed, individuals with 2 SDV events leading to *ATM* null status tended to have an earlier clinical diagnosis compared to individuals with other genotypes (79/235; *P* <  0.05, log-rank test), even after factoring out 13 patients with a known hypomorphic variant (c.5763-1050A>G, prevalent in the UK) as one of the pathogenic variants (Fig. 1d).

In addition, we detected six insertion events (100 bp–4 kb) fully contained within introns. These included two pseudogene insertions (4 kb; one homozygous patient), one tandem duplication (382 bp; Supplementary Fig. 4), and three Alu retrotransposon insertions (around 280 bp, one *n* = 1 event; around 131 bp, one *n* = 2 event). Although these intronic events do not directly disrupt the coding sequence (and therefore were not counted as SDVs), they could still contribute to loss of function by activating cryptic splice sites or disrupting canonical splicing. For instance, the 382-bp tandem duplication was strongly predicted by SpliceAI^20^ to cause inclusion of an out-of-frame (256-bp) pseudoexon in intron 33, which would lead to premature translational termination and nonsense-mediated decay (Supplementary Fig. 4).

**Extended Data Fig. 3:**
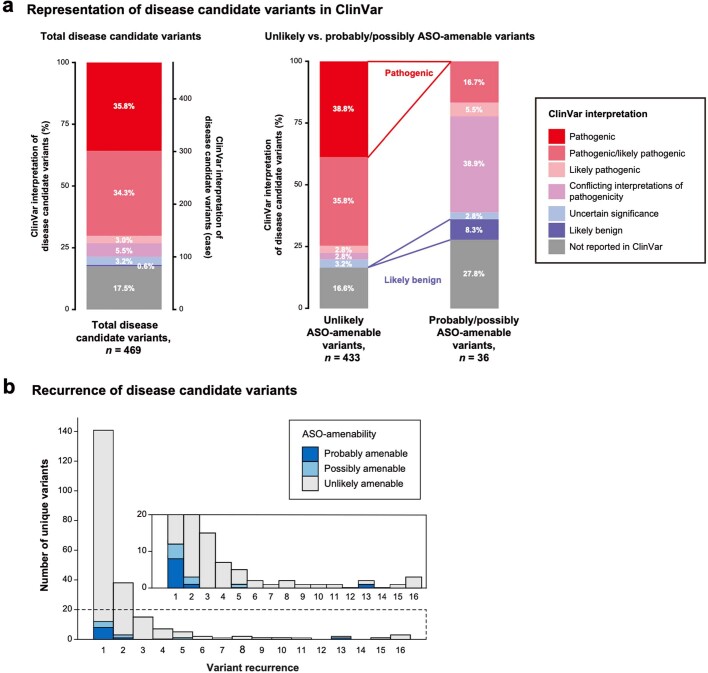
Disease candidate variant characteristics. **a**, Representation of disease candidate variants in ClinVar. Total disease candidate variants (*n* = 469) by ClinVar interpretation category and comparison of the composition of ClinVar interpretation categories of probably/possibly ASO-amenable variants versus unlikely ASO-amenable variants are shown. 73% (343/469) of all the disease candidate mutational events had been classified in ClinVar as pathogenic or likely pathogenic, 8.7% (41/469) as conflicting interpretations of pathogenicity or uncertain significance, 0.6% (3/469) as likely benign, and 17% (82/469) had not been reported in ClinVar. We note in particular that the three likely benign mutational events (c.3489C>T, *n* = 1; c.2639-22_2639-20del, *n* = 2) have strongly predicted mis-splicing potential and have not been found in gnomAD v3.1 and TOPMed freeze 8 (Supplementary Table 1); because no other variants can explain the clinical diagnosis in the respective patients, we considered them as disease candidate variants. **b**, Recurrence of disease candidate variants. The recurrence distribution of disease candidate variants in 235 patients in the ATCP cohort is depicted. Homozygous variants are double-counted. The inset shows a magnified view of the dotted box region in the main graph.

Evidence supporting the remaining 83 disease candidate variants (mostly missense and intronic and synonymous variants) was obtained from previous interpretations in ClinVar, their rarity and/or other predictions of functional impact (for details of the procedure for determining disease candidate variants, see Methods; for the overlap between disease candidate variants and ClinVar variants, see Extended Data Fig. 3a).

Of the variants encountered, 64% (141/220) were singletons (that is, appeared only once in this cohort). The remaining 36% (79/220) were observed at least twice. The most highly recurrent variant was c.5932G>T (p.Glu1978Ter), present in 16 individuals (Extended Data Fig. 3b and Supplementary Table 8).

## ASO-amenable variants

**Fig. 2:**
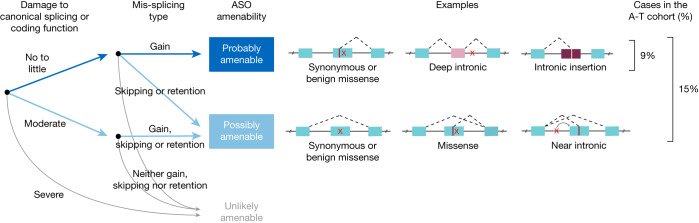
Taxonomy of ASO-amenable variants. Two-step logic for classifying the amenability of genetic variants to splice modulation rescue (probably, possibly or unlikely). First, each variant is evaluated for its damaging impact on either canonical splicing or protein-coding function, on the basis of SpliceAI, MaxEntScan, LaBranchoR and REVEL. Second, each variant is evaluated for potential mis-splicing impact: gain of a splice site, exon skipping or intron retention. See Methods for complete details.

We developed a taxonomy to classify disease-causing variants on the basis of their predicted amenability to splice-switching ASO intervention, using categories of ‘probably’, ‘possibly’ or ‘unlikely’ (further details of the taxonomy are provided in Fig. 2 and Methods). In brief, the taxonomy addresses two questions:

(1) Does the variant directly damage the coding function and/or a canonical splice site? If yes, by how much? Damage to the coding function was assessed by using in silico predictor (REVEL^21^) and experimental evidence from the literature; damage to canonical splicing function was assessed by using SpliceAI^20^, MaxEntScan^24^ and LaBranchoR^25^.

(2) Does the variant lead to a type of mis-splicing that might be rescued by an ASO? Mis-splicing events can be induced when a variant creates a novel splice site or weakens exon definition (through disruption of canonical donor–acceptor sites or splicing enhancers, or creation of splicing silencers). Of the two types, a gain of a splice site is more amenable to rescue than a weakening of exon definition, because repressing a cryptic splice site can often be achieved by simply blocking the site with an ASO, whereas promoting a weakened exon requires identifying and blocking silencer elements within or near the exon. These possibilities were assessed with in silico splicing predictions based on SpliceAI and MaxEntScan, as well as experimental evidence.

**Fig. 3:**
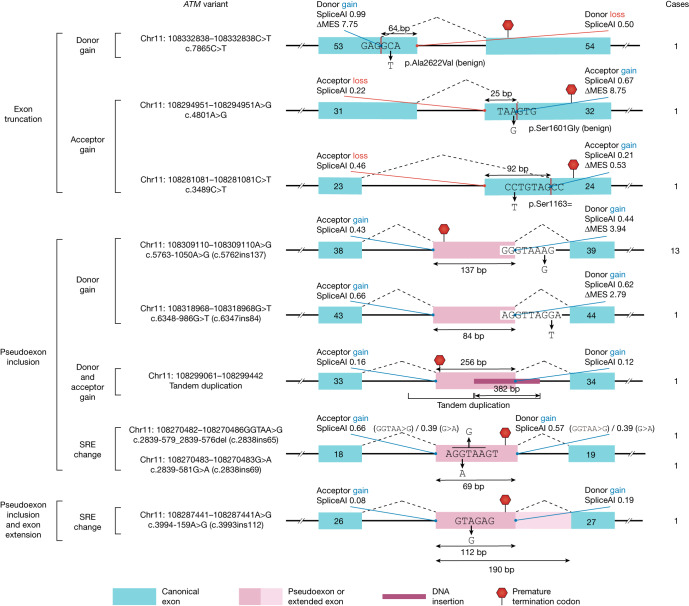
Probably ASO-amenable variants. Cases indicates the number of individuals with the variant in the ATCP cohort of 235 individuals. The homozygous *DUSP16* pseudogene insertion event (Supplementary Table 3) is not shown. For the full list of probably ASO-amenable variants, see Supplementary Table 9. SRE, splicing regulatory element; ΔMES, change in MaxEntScan score when a given variant is introduced.

**Extended Data Fig. 4:**
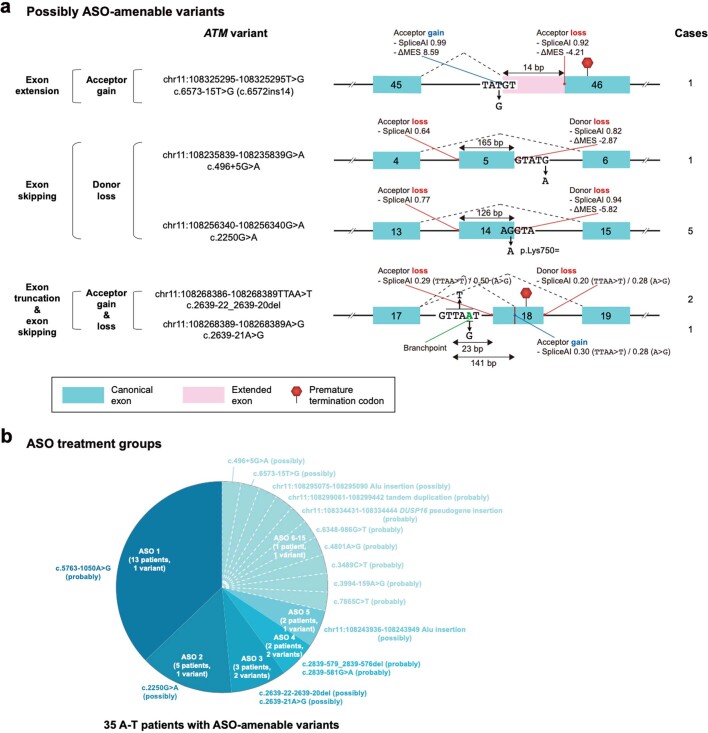
Possibly ASO-amenable variants and ASO treatment groups. **a**, Cases indicate the number of patients having the variant in the ATCP cohort of 235 patients. Three Alu insertion events are not depicted here. For the full list of possibly ASO-amenable variants, see Supplementary Table 9. ΔMES, change in MaxEntScan score when a given variant is introduced. **b**, 35 patients with A-T with ASO-amenable variants can be divided into 15 ASO treatment groups; patients in each group can be treated with the same ASO therapy.

Using this taxonomy, we encountered 36 probably/possibly ASO-amenable mutational events (representing 17 unique variants) in 35 patients from the ATCP cohort (15%, 35/235; Fig. 1a and Supplementary Table 9). Specifically, 22 individuals (9%, 22/235) had at least one probably amenable variant (Fig. 3), and 13 (6%, 13/235) had a possibly amenable variant (Extended Data Fig. 4a). Four of the 17 probably/possibly amenable variants were found in more than a single patient, with the most highly recurrent variant (c.5763-1050A>G) present in 13 of the 235 patients. Only 22% (8/36) of the ASO-amenable variants had been previously classified in ClinVar as pathogenic or likely pathogenic (Extended Data Fig. 3a); 50% (18/36) had been previously classified as likely benign, uncertain significance or conflicting interpretations of pathogenicity; and 28% (10/36) were not reported in ClinVar (as opposed to around 17% for non-ASO-amenable variants). These findings suggest that ASO-amenable variants are often misinterpreted and underrepresented in ClinVar.

The majority (26/36, 72%) of the probably/possibly ASO-amenable mutational events were deep intronic, defined as being located more than 20 nucleotides away from an exon (Fig. 1b). These included six SV events that were fully contained in an intron (Supplementary Fig. 4 and Supplementary Tables 3 and 9). Restricting the analysis to exons and the flanking intronic regions (20 or more nucleotides from splice junctions), the yield of ASO-amenable variants was only 4% (1%, probably; 3%, possibly). Hence, WGS (9%, probably; 6%, possibly) provides a 3.5-fold (counting all amenable variants; 35 cases versus 10 cases) to 7-fold (counting only probably amenable variants; 22 cases vs. 3 cases) higher ‘ASO therapeutic yield’ than whole-exome sequencing (WES; Fig. 1a).

**Fig. 4:**
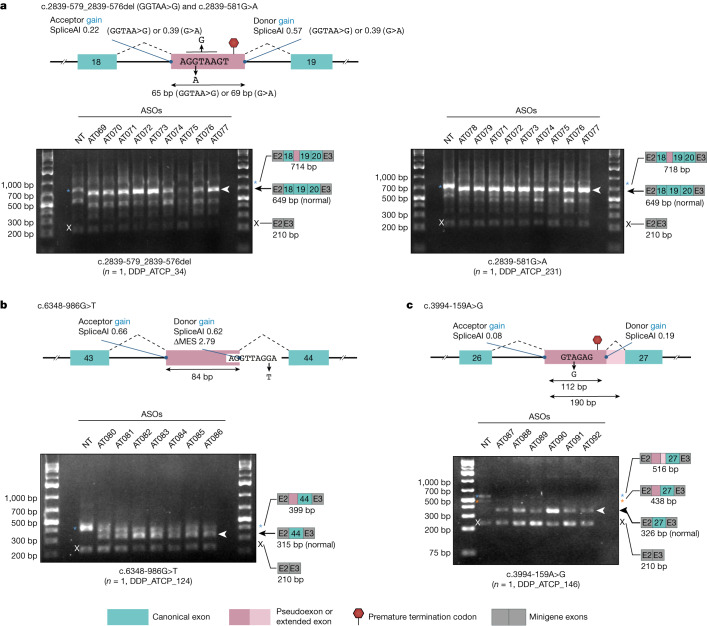
Validation of ASO amenability. **a**–**c**, For four probably ASO-amenable variants for which a minigene-based splicing assay is robustly established (**a**, c.2839-579_2839-576del (GGTAA>G) and c.2839-581G>A; **b**, c.6348-986G>T; and **c**, c.3994-159A>G), small-scale ASO screening was performed, which showed that the mis-splicing events caused by all tested variants can be rescued by ASOs. NT, NT-22 (a non-targeting ASO). Blue asterisks, white (or black) arrows and white (or black) ‘x’ marks indicate bands validated by Sanger sequencing (orange asterisk indicates a band not validated by Sanger sequencing). ΔMES, change in MaxEntScan score when a given variant is introduced. For gel source data, see Supplementary Fig. 1.

**Extended Data Fig. 5:**
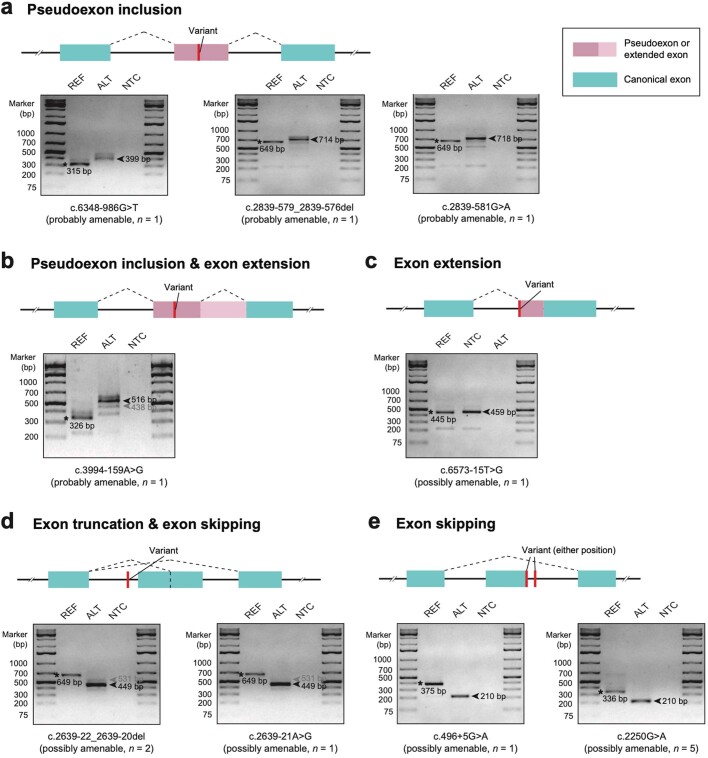
Mis-splicing validation by minigene assay. **a**, Validation of the pseudoexon inclusion effects of ASO-amenable variants, c.6348-986G>T (probably), c.2839-579_2839-576del (probably), and c.2839-581G>A (probably). **b**, Validation of complex mis-splicing effects (pseudoexon inclusion and exon extension) of an ASO-amenable variant, c.3994-159A>G (probably). **c**, Validation of exon extension effects of an ASO-amenable variant, c.6573-15T>G (possibly). **d**, Validation of complex mis-splicing effects (exon truncation and exon skipping) of ASO-amenable variants, c.2639-21A>G (possibly) and c.2639-22_2639-20del (possibly). **e**, Validation of exon skipping effects of ASO-amenable variants, c.496+5G>A (possibly) and c.2250G>A (possibly). For **a**–**e**, black asterisks and black arrows indicate PCR products confirmed by gel extraction and Sanger sequencing; grey arrows indicate bands not validated by Sanger sequencing (Supplementary Table 12). REF, reference allele; ALT, alternative allele; NTC, no-template control. For gel source data, see Supplementary Fig. 1.

We used RNA sequencing (RNA-seq) or experimental minigene splicing assays to benchmark the accuracy of our predictions. For two probably ASO-amenable variants (c.7865C>T, c.5763-1050A>G), patient fibroblasts were available for RNA-seq; for nine additional probably/possibly ASO-amenable variants, gDNA samples were available, and experimental minigene splicing assays were successfully established (for more details about minigene assays, see Methods and Supplementary Tables 10–12). All tested variants (11/11) yielded the predicted mis-splicing consequences (for RNA-seq, see the following sections; for minigene assays, see Extended Data Fig. 5). Furthermore, we conducted ASO screens for six probably ASO-amenable variants (two based on patient fibroblasts and four based on minigene assays), and successfully identified ASOs capable of correcting mis-splicing for all six (see the following sections for patient-fibroblast-based screens, Fig. 4 for minigene-based screens and Supplementary Table 9 for a summary).

Overall, four ASO-amenable variants were recurrent within the cohort: c.5763-1050A>G, c.2250G>A, chr11:108243936-108243949insAlu and c.2639-22_2639-20del were found in thirteen, five, two and two individuals, respectively (Supplementary Table 9). In addition, several different ASO-amenable variants had splicing consequences that appeared addressable with a single ASO; for instance, both c.2839-579_2839-576del and c.2839-581G>A result in the inclusion of the same pseudoexon (Fig. 3). We therefore subdivided variants into ‘treatment groups’, each potentially addressable with a single ASO drug. On the basis of these patterns, around 70% (24/35) of amenable individuals were predicted to be treatable with a total of five different splice-switching ASOs, whereas developing splice-switching treatments for all 35 individuals would require 15 distinct therapeutic ASO drugs (Extended Data Fig. 4b and Supplementary Table 9).

## Selection of variants for ASO development

Two ASO-amenable *ATM* variants were selected for further development. The first was c.5763-1050A>G (Fig. 3). The variant, located deep in intron 38, results in the inclusion of a 137-bp pseudoexon, thereby causing a frameshift in the resulting mature product^26^. This variant is associated with a mild A-T phenotype owing to partial leakiness of its gain-of-splicing effect. It is a founder variant in the UK, with an estimated disease allele frequency of 18% in individuals with A-T in the UK (refs. ^27,28^). In the ATCP cohort, composed predominantly of individuals from the USA, it was found in a compound heterozygous state in 13 unrelated patients, representing a population frequency of 5.5% (13/235) and a disease allele frequency of 2.8% (13/469).

**Extended Data Fig. 6:**
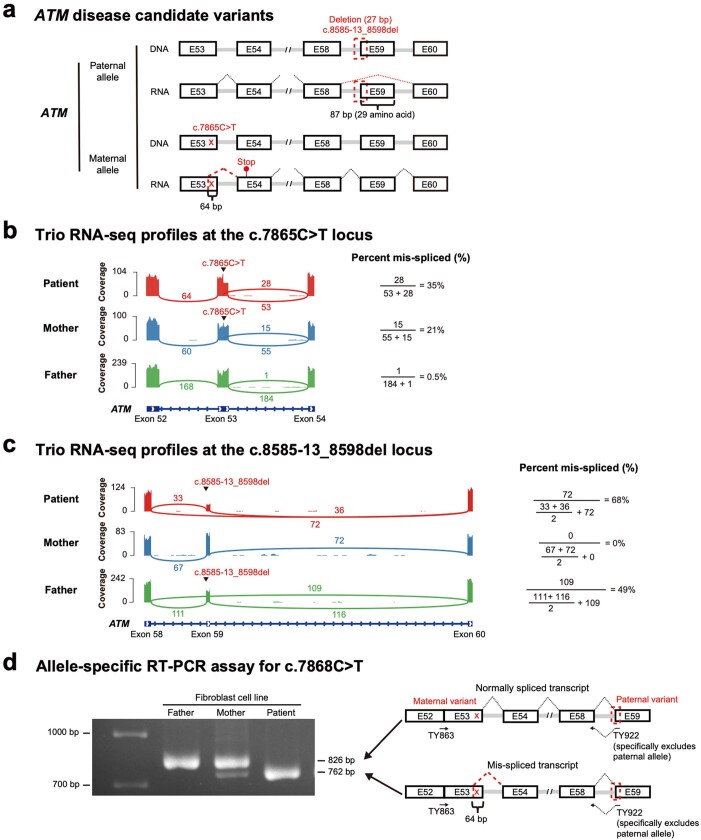
A patient with A-T with c.7865C>T. **a**, The paternal and maternal variants of a currently six-year-old patient with c.7865C>T. Note: this case is not in the ATCP cohort and is not the same case as DDP_ATCP_520, who is a currently 20-year-old patient with c.7865C>T. The paternally inherited c.8585-13_8598del variant disrupts the canonical splice acceptor site of exon 59, leading to the skipping of exon 59 (87 nucleotides), and removes critical residues from the highly conserved PI3K/PI4K kinase domain of the ATM protein. Although it is in-frame, because the skipped region contains a critical kinase domain, it results in complete loss of function. The maternally inherited c.7865C>T variant creates a novel splice site in exon 54, leading to mis-splicing that results in an out-of-frame truncation of 64 nucleotides from the 3′ end of exon 53. **b**, RNA-seq analysis of the trio’s fibroblast cell lines shows the gain-of-splicing effect in exon 53 by c.7865C>T in the patient and the mother. **c**, RNA-seq analysis of the trio’s fibroblast cell lines shows the gain-of-splicing effect in exon 59 by c.8585-13_8598del in the patient and the father. **d**, Determination of the gain-of-splicing effect of c.7865C>T in the trio’s fibroblast cell lines by allele-specific PCR that is specifically designed to exclude the non-target (parental) allele. This result of the allele-specific PCR on the naive patient fibroblasts was replicated in 11 additional biologically independent experiments (*n* = 3, initial; *n* = 2, fine-tuning; *n* = 3, AT008 dose–response; *n* = 3, AT026 dose–response; Fig. 5a and Extended Data Fig. 8c). For gel source data, see Supplementary Fig. 1.

The second variant was c.7865C>T (Fig. 3). This variant has been previously identified in the homozygous state in patients with classical (severe) A-T. A lymphoblastoid cell line with this variant had no residual protein or enzymatic activity^29^ (A. M. R. Taylor, personal communication); that is, this variant is a null variant. This variant is predicted to have a benign coding effect (p.Ala2622Val; predicted benign by REVEL), with a pathogenic effect that is mediated by mis-splicing: it creates a strong splice donor site within exon 53 (of 63 exons), causing truncation of the exon by 64 bp, which results in frameshift and subsequent premature translational termination. Inhibition of this splice donor site with a morpholino oligonucleotide has been previously shown to rescue the cellular phenotype of a patient-derived cell line^30^.

c.7865C>T was encountered in one individual in the ATCP cohort (DDP_ATCP_520, currently 20 years old). Separately, we also identified a second, younger (one year old at referral; currently six years old) child with A-T with this variant as well (Extended Data Fig. 6a and Supplementary Note 4). Whereas most patients with A-T are diagnosed after the initial onset of symptoms (Supplementary Table 1), this child was diagnosed as an infant on the basis of an abnormally low T cell receptor excision circle (TREC) count. TREC assays are used in newborn screening to identify infants at risk for severe combined immune deficiency (SCID), but have also incidentally identified cases of A-T (ref. ^31^). Exome sequencing in this child showed compound heterozygosity for two *ATM* variants: c.7865C>T and c.8585-13_8598del (confirmed by trio Sanger sequencing; Supplementary Table 6). The latter is a 27-bp deletion at the intron–exon junction of exon 59, strongly predicted to cause complete loss of function (Supplementary Note 4). This combination of variants predicted a classical, early-onset A-T phenotype (Fig. 1d).

## Correcting c.7865C>T and c.5763-1050A>G

A fibroblast cell line was established from the younger patient with c.7865C>T. PCR with reverse transcription (RT–PCR) and RNA-seq analysis of splicing patterns in this cell line showed an abnormal truncation of exon 53 owing to premature splice donor site usage, consistent with previous studies^30^ (Extended Data Fig. 6b,c). Analysis using allele-specific PCR primers (designed to exclude the non-target c.8585-13_8598del allele) showed that *ATM* mis-splicing by c.7865C>T is complete, without detectable leakiness (Extended Data Fig. 6d).

**Fig. 5:**
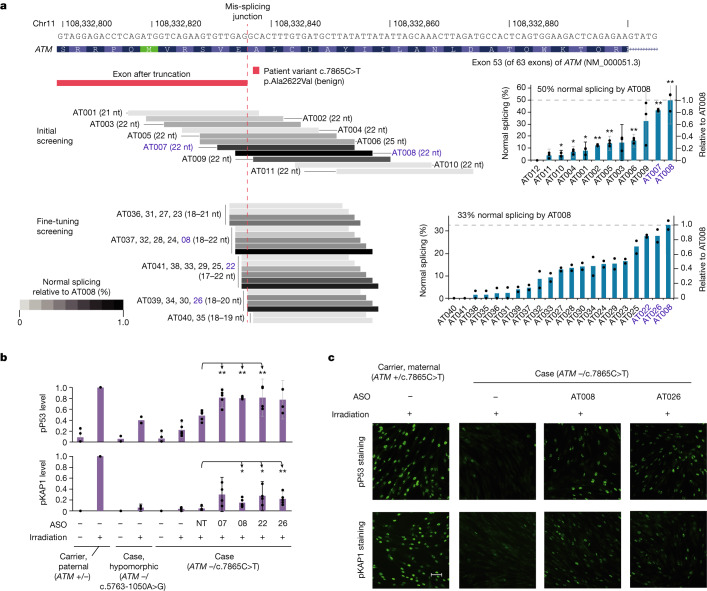
Preclinical development of AT008 (atipeksen). **a**, Design and screening of ASOs targeting c.7865C>T. Biologically independent experiments (independent transfections) were conducted (*n* = 3, initial; *n* = 2, fine-tuning). For some ASOs in the fine-tuning screening, the first two letters are omitted. Error bars, mean ± 95% confidence interval (shown only for conditions with *n* ≥ 3). A one-sample two-tailed *t*-test was used to assess statistical significance; means were compared to a constant value of 0 because no background normal splicing was observed in cells that were mock-transfected or transfected with non-targeting ASOs. *P < 0.05; **P < 0.01. AT010, *P = 0.0441; AT004, *P = 0.0240; AT001, *P = 0.0453; AT002, **P = 0.0010; AT005, **P = 0.0093; AT006, **P = 0.0060; AT007, **P = 0.0001; AT008, **P = 0.0082. Four top-performing ASOs (blue letters) were selected for further validation (**b**,**c**). For RT–PCR gel source data, see Supplementary Fig. 1. **b**, ASO-mediated restoration of irradiation-induced ATM signalling in patient fibroblasts, measured by immunoblotting. 07, 08, 22 and 26 represent AT007, AT008, AT022 and AT026, respectively; NT, NT-22 (a non-targeting ASO). pP53, phospho-P53; pKAP1, phospho-KAP1. Biologically independent experiments (independent transfections) were conducted: pP53 (*n* = 2, hypomorphic cases ± irradiation; *n* = 4, AT022, AT026; *n* = 5, the other conditions), pKAP1 (*n* = 4, AT007 and AT022; *n* = 5, the other conditions). Error bars, mean ± 95% confidence interval (shown only for conditions with *n* ≥ 3). A two-sample (comparing each condition to NT-22) two-tailed *t*-test was used for statistical analysis. *P < 0.05; **P < 0.01. For pP53, AT007, **P = 0.0024; AT008, **P = 0.0001; AT022, **P = 0.0471. For pKAP1, AT008, *P = 0.0201; AT022, *P = 0.0175; AT026, **P = 0.0073. Representative blot images are shown in Extended Data Fig. 8a. For blot source data, see Supplementary Fig. 1. **c**, ASO-mediated restoration of irradiation-induced ATM signalling in patient fibroblasts, measured by immunofluorescence staining. Scale bar, 50 μm. For a quantitative summary of the complete results, see Extended Data Fig. 8b.

**Extended Data Fig. 7:**
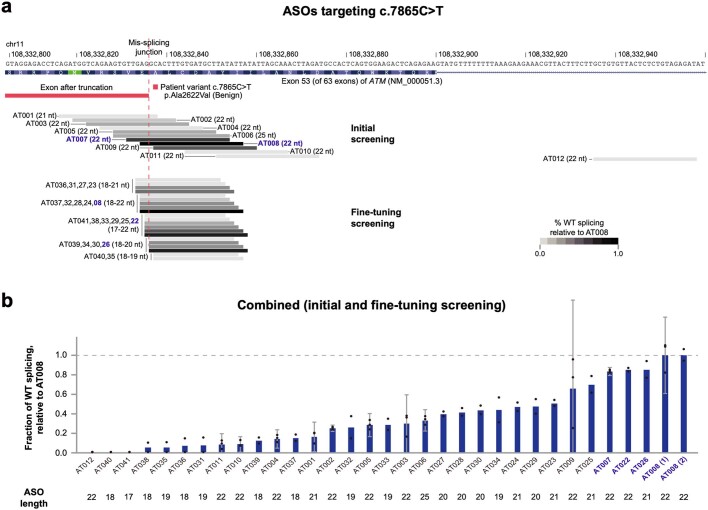
Design and screening of ASOs targeting c.7865C>T. **a**, The initial and fine-tuning rounds of screening of ASOs for rescuing the mis-splicing effects of c.7865C>T in patient fibroblasts. It is depicted as in Fig. 5a, except the twelfth ASO of the initial screening round, AT012, is shown. AT012, designed to inhibit an intronic splicing silencer downstream of exon 53, showed no efficacy. **b**, Combined analysis of initial and fine-tuning rounds of screening for c.7865C>T. As AT008 was used in both the initial and fine-tuning rounds of screening (Fig. 5a), the ASO efficacy measurements of the two rounds were combined into a single figure by normalizing the measurements to the matched mean efficacy of AT008 of each round. All statistical information (sample size, error bar, statistical test, *P* value) is as in Fig. 5a, because this is a reanalysis of the data from that figure. For RT–PCR gel source data, see Supplementary Fig. 1.

To attempt to correct the mis-splicing, we designed an initial set of 12 ASOs, targeting either the novel splice donor site in the exon (AT001-AT011; Fig. 5a and Supplementary Table 13) or a nearby predicted splice regulatory element (AT012; Extended Data Fig. 7a). Several of these ASOs were effective in restoring normal splicing on allele-specific RT–PCR in patient fibroblasts (Supplementary Table 14), with two designs (AT007 and AT008) showing the most promise. Both ASOs were 22 nt long and were offset only by 3 nt, revealing an efficacy hotspot. A set of 20 additional ASOs was designed against this region and tested for the mis-splicing rescue in a secondary screen (Extended Data Fig. 7b), revealing additional ASOs with an efficacy similar to that of AT008.

**Extended Data Fig. 8:**
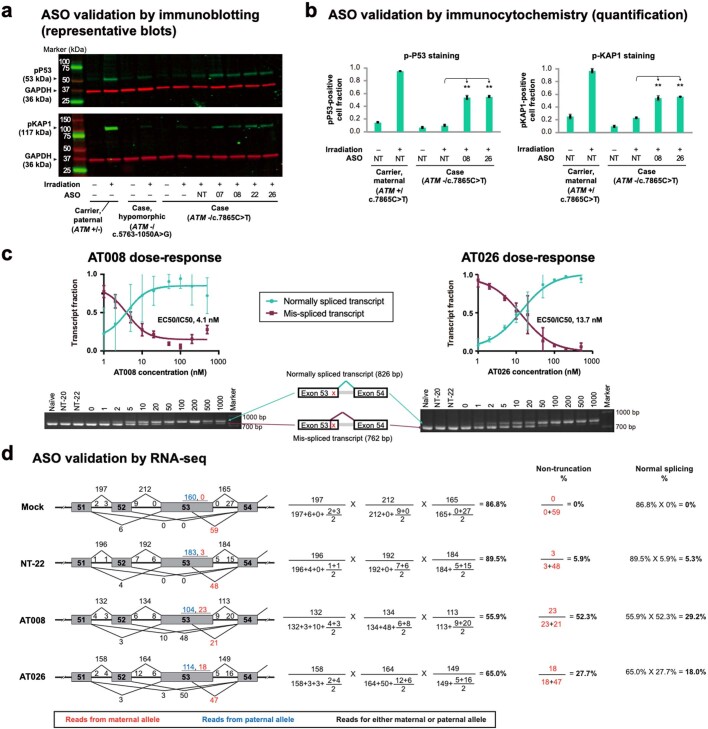
Validation of ASOs targeting c.7865C>T. **a**, Representative blots of immunoblotting analysis of the rescue of ATM function by the ASOs targeting c.7865C>T. Phosphorylation of P53 and KAP1 in response to irradiation-induced DNA damage. Five independent replicate experiments were performed and band intensities were quantified to generate Fig. 5b. For AT007, AT008, AT022, and AT026, “AT0” was omitted. NT represents NT-22, a non-targeting ASO. For blot source data, see Supplementary Fig. 1. **b**, Quantification of immunocytochemistry analysis of the rescue of ATM signalling by the ASOs targeting c.7865C>T, demonstrating recovery of P53 and KAP1 phosphorylation following treatment with AT008 and AT026. For representative micrographs, see Fig. 5c. For quantification, 10 microscope field images were taken from each of three biologically independent wells for each condition. The % positive cells of the 10 microscope field images were averaged to yield the % positive cells value for each well. Error bars, mean of the three biological replicates ± 95% confidence interval. Two-sample (comparing each condition to NT-22) two-tailed *t*-test was performed. *, *P* < 0.05; **, *P* < 0.01. For pP53, AT008, **P = 9.5E-7; AT026, **P = 2.5E-7. For pKAP1, AT008, **P = 5.0E-6; AT026, **P = 8.5E-8. **c**, Dose–response analysis of rescue of *ATM* mis-splicing by AT008 and AT026. NT-20 and NT-22 are non-targeting ASOs. The fraction of the intensity of the normally spliced transcript of the total intensity of the normally and abnormally spliced transcripts was calculated for each condition. Three biologically independent experiments (independent electroporations) were conducted. Error bars, mean ± 95% confidence interval. The regression curves and EC_50_/IC_50_ values were calculated by using “log(agonist) vs. response - Variable slope” model in GraphPad Prism 8. 1,000 nM concentrations of AT008 and AT026 were excluded from analysis because they showed cell-level toxicity and anomalous RT–PCR results. Representative agarose gel images are shown at the bottom of the dose–response curves. For gel source data, see Supplementary Fig. 1. **d**, RNA-seq analysis of the rescue of mis-splicing in patient fibroblasts by AT008 and AT026. Numbers marked in red and blue represent the number of reads that can be unambiguously determined to be originated from either paternal or maternal allele. The percentage of functional splicing was calculated by scoring the fraction of transcripts that were without exon 52/53/52-53 skipping, intron 51/52/53 retention, and exon 53 truncation. pP53, phospho-P53; pKAP1, phospho-KAP1.

Four ASOs (AT008, AT007, AT022 and AT026) were selected for further validation. To determine whether the ASOs could rescue *ATM* cellular function in addition to splicing, we studied their ability to restore cellular defence responses to radiation, as measured by phosphorylation of P53 and KAP1, two key downstream effectors in the ATM-mediated DNA damage response pathway^32^. Immunoblotting experiments using antibodies against phospho-P53 and phospho-KAP1 showed ASO-mediated rescue of radiation-induced ATM signalling in patient cell lines (Fig. 5b and Extended Data Fig. 8a). Partial retention of ATM kinase activity has been shown to be associated with increased median age of diagnosis and age of death, and later onset of neurological symptoms^33^. In particular, after ASO treatment, the levels of phospho-P53 and phospho-KAP1 exceeded those measured in fibroblasts bearing the hypomorphic c.5763-1050A>G variant, supporting the potential clinical relevance of this rescue.

AT008 (22 nt) and AT026 (20 nt) were selected for additional characterization. Both ASOs were capable of rescuing radiation-induced P53 and KAP1 activation by immunocytochemistry (Fig. 5c and Extended Data Fig. 8b), confirming the previous immunoblot results. Dose–response analysis showed that AT008 and AT026 had half-maximum effective concentration (EC_50_) and half-maximum inhibitory concentration (IC_50_) values in the nanomolar range (Extended Data Fig. 8c), consistent with previously reported ASOs for clinical use^1^, but AT008 was three times more potent than AT026. AT008 also showed substantially greater suppression of abnormal splicing compared to AT026 in RNA-seq experiments from treated patient fibroblasts (52% versus 28% correction for AT008 versus AT026, versus 0–6% in controls; Extended Data Fig. 8d). Both ASOs were also found to have the undesirable effect of increasing exon 53 skipping (although we noted that ASOs that target intronic splicing silencers could potentially avoid exon skipping, AT012, designed to explore this strategy, did not show any promising activity; Extended Data Fig. 7). Nevertheless, even adjusting for this undesirable effect, AT008 and AT026 boosted the levels of functional *ATM* transcript from 0–5% (untreated patient fibroblasts) to 29% (AT008) and 18% (AT026), in addition to restoring P53 and KAP1 phosphorylation as described above. In silico analyses showed that AT008 and AT026 had clean off-target profiles in the human genome (no binding sites within exons, or within introns up to 5 kb from the nearest exon, allowing end-trimming of up to 6 nt and internal mismatches of up to 2 nt; Supplementary Figs. 5 and 6). These off-target profiles compared favourably with nusinersen and milasen, two clinically administered intrathecal ASOs (Supplementary Figs. 7 and 8). In vitro cellular toxicity assays showed that AT008 is as tolerable as a non-targeting control ASO (Supplementary Fig. 9).

**Extended Data Fig. 9:**
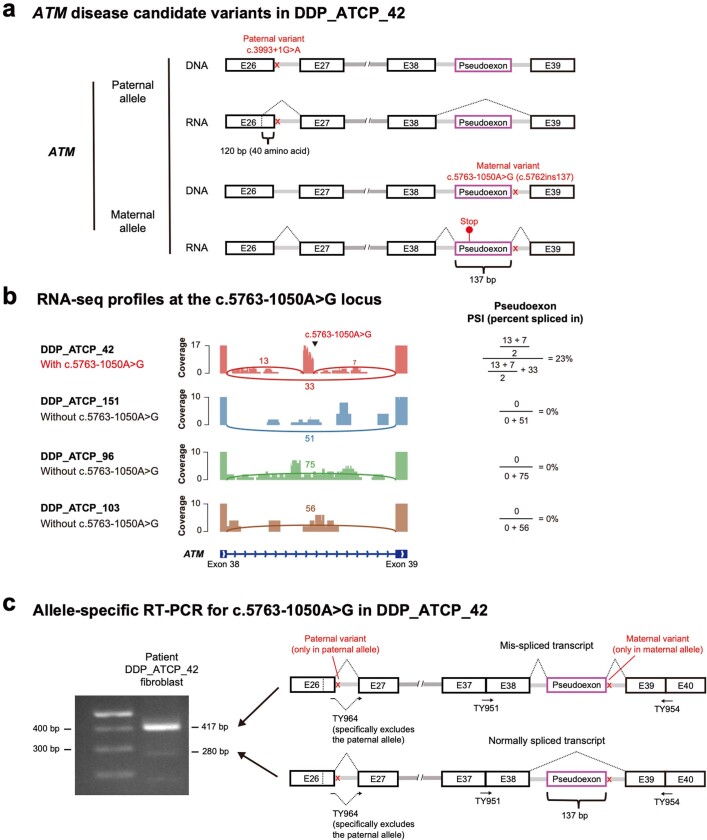
A patient with A-T with c.5763-1050A>G. **a**, c.5763-1050A>G in one allele of DDP_ATCP_42 creates a novel splice site that causes the out-of-frame (137 bp) inclusion of a pseudoexon and premature translation termination of *ATM*. c.3993+1G>A in the other allele of the patient destroys a splice donor site, which leads to the use of a cryptic splice donor site in exon 26. The resulting mis-splicing truncates 120 nt from the 3’ end of exon 26. **b**, RNA-seq analysis of whole-blood samples from the patient and three other patients without c.5763-1050A>G shows the pseudoexon inclusion effect between exon 38 and exon 39 by c.5763-1050A>G. **c**, Determination of the pseudoexon inclusion effect of c.5763-1050A>G in the patient’s fibroblast cell line by allele-specific RT–PCR that is specifically designed to exclude the non-target allele (c.3993+1G>A). Because the distance between the two *ATM* variants were too far (~2 kb) to distinguish the two bands representing normally and abnormally spliced products (which differ by 137 bp) on an agarose gel, a nested PCR was performed. This result of the allele-specific PCR on the naive patient fibroblasts was replicated in 7 additional biologically independent experiments (*n* = 3, initial; *n* = 2, fine-tuning; *n* = 4; Extended Data Fig. 10b). For gel source data, see Supplementary Fig. 1.

**Extended Data Fig. 10:**
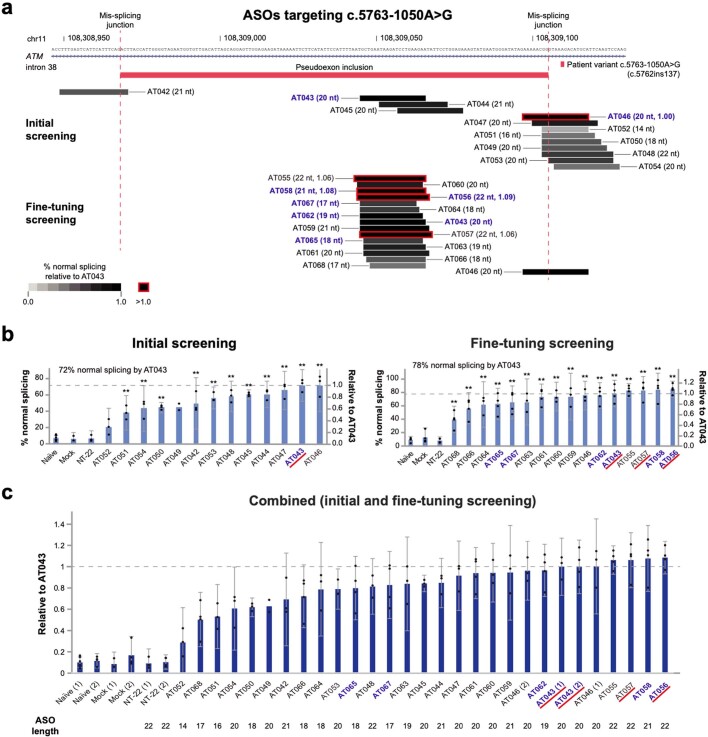
Design and screening of ASOs targeting c.5763-1050A>G. **a**,**b**, The initial and fine-tuning rounds of screening of ASOs for rescuing the mis-splicing by c.5763-1050A>G. These ASOs were tested in the fibroblasts from DDP_ATCP_42 by using the allele-specific RT–PCR assay (Extended Data Fig. 9c). The positions and efficacy of the ASOs are illustrated (**a**, mean efficacy is represented using a grey scale; **b**, mean efficacy, along with a 95% confidence interval, is illustrated using bar graphs). Biologically independent experiments (independent transfections) were conducted for initial screening (*n* = 6, naive; *n* = 2, AT049, one of the three experiments was excluded from statistical analysis as it showed a highly anomalous result, Supplementary Fig. 1; *n* = 3, the other conditions) and fine-tuning screening (*n* = 3, AT046, AT059, AT060, AT063, AT064; *n* = 4, the other conditions). Error bars, mean ± 95% confidence interval (shown only for conditions with *n* ≥ 3). Two-sample (comparing each condition to NT-22) two-tailed *t*-test was performed for statistical analysis. *, *P* < 0.05; **, *P* < 0.01. For initial screening, AT051, **P = 0.0046; AT054, **P = 0.0058; AT050, **P = 0.0002; AT042, **P = 0.0048; AT053, **P = 0.0002; AT048, **P = 0.0004; AT045, **P = 3.3E-5; AT044, **P = 0.0003; AT047, **P = 0.0006; AT043, **P = 0.0002; AT046, **P = 0.0011. For fine-tuning screening, AT068, **P = 0.0030; AT066, **P = 0.0006; AT064, **P = 0.0006; AT065, **P = 0.0004; AT067, **P = 0.0042; AT063, **P = 0.0004; AT061, **P = 4.1E-5; AT060, **P = 3.2E-5; AT059, **P = 0.0002; AT046, **P = 2.9E-5; AT062, **P = 3.7E-5; AT043, **P = 3.6E-5; AT055, **P = 8.8E-7; AT057, **P = 2.8E-5; AT058, **P = 6.8E-5; AT056, **P = 1.6E-6. **c**, Combined analysis of initial and fine-tuning rounds of screening for c.5763-1050A > G. As AT043 was used in both the initial and fine-tuning screening, the ASO efficacy measurements of the two rounds were combined into a single figure by normalizing the measurements to the matched mean efficacy of AT043 of each round. All statistical information (sample size, error bar, statistical test, *P* value) is as in **b** because this is a reanalysis of the data from that panel. For **a**–**c**: six top-performing ASOs (blue letters) were selected from each length group (picking one ASO from each of the 17-, 18-, 19-, 20-, 21-, and 22-mers) for further validation (Extended Data Fig. 11a,b); three ASOs (underlined in red) were subjected to additional RNA-seq validation (Extended Data Fig. 11c); for RT–PCR gel source data, see Supplementary Fig. 1.

**Extended Data Fig. 11:**
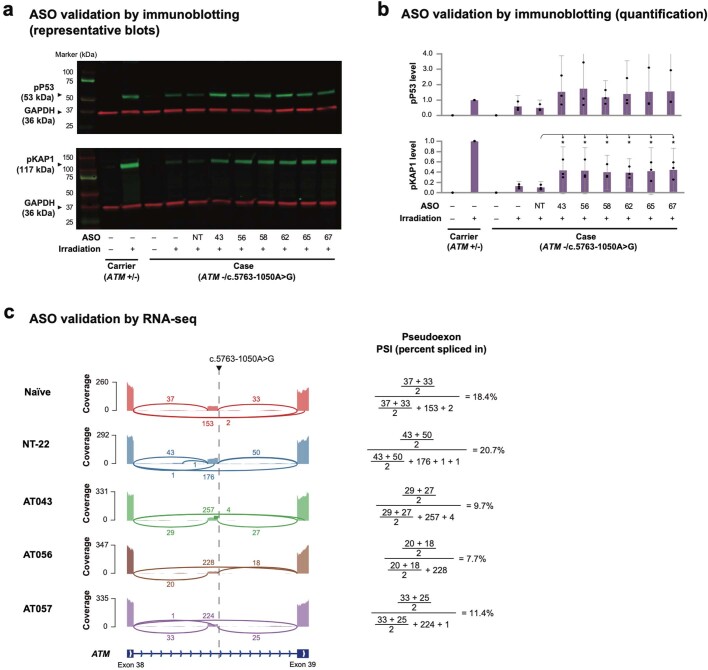
Validation of ASOs targeting c.5763-1050A>G. **a**, Representative immunoblotting results demonstrating rescue of ATM function by ASOs targeting c.5763-1050A>G. pP53, phospho-P53; pKAP1, phospho-KAP1. “AT0” was omitted in AT043, AT056, AT058, AT062, AT065, and AT067. NT represents NT-22, a non-targeting ASO. **b**, Quantification of the immunoblots using GAPDH as the loading control. Irradiated, control fibroblasts (*ATM* +/−) were used for normalization. All six tested ASOs showed comparable efficacy in restoring the phosphorylation of the two downstream effectors. Three biologically independent experiments (independent transfections) were conducted for pP53 and pKAP. Two-sample (comparing each condition to NT-22) two-tailed *t*-test was performed to assess the statistical significance of the means normalized to carrier case with irradiation. Error bars, mean ± 95% confidence interval. *, *P* < 0.05. For pKAP1, AT043, *P = 0.0386; AT056, *P = 0.0395; AT058, *P = 0.0232; AT062, *P = 0.0145; AT065, *P = 0.0447; AT067, *P = 0.0287. For **a**,**b**, for blot source data, see Supplementary Fig. 1. **c**, RNA-seq validation of ASOs targeting c.5763-1050A>G. RNA-seq analysis of the fibroblast cell line established from DDP_ATCP_42 shows the pseudoexon inclusion effect of c.5763-1050A>G can be mitigated by AT043, AT056, and AT057. Among the tested ASOs, AT056 showed the highest efficacy in reducing the pseudoexon inclusion, making it the lead candidate. NT-22, non-targeting ASO.

Parallel experiments were performed for the c.5763-1050A>G variant. A fibroblast cell line was established from a patient with A-T from the ATCP cohort (DDP_ATCP_42) and used to confirm the mis-splicing consequences of c.5763-1050A>G (Fig. 3 and Extended Data Fig. 9). ASOs were designed to block the pseudoexon usage associated with this allele, and screening in patient fibroblasts successfully identified a lead ASO that was capable of rescuing ATM function (Extended Data Figs. 10 and 11, Supplementary Note 5 and Supplementary Figs. 9 and 11).

## Individualized trial for *ATM* c.7865C>T

The c.7865C>T-targeting ASO AT008 (renamed atipeksen) was selected for further clinical development. It was chosen because of the association of c.7865C>T with severe disease (classical A-T), the robustness of atipeksen-mediated RNA and cellular functional rescue and the opportunity for early therapeutic intervention before the onset of major neurological morbidity in the previously identified young child with this variant. (Note that the other identified individual with this variant, DDP_ATCP_520, was not considered to be a suitable candidate for clinical intervention owing to the advanced stage of the disease).

**Extended Data Fig. 12:**
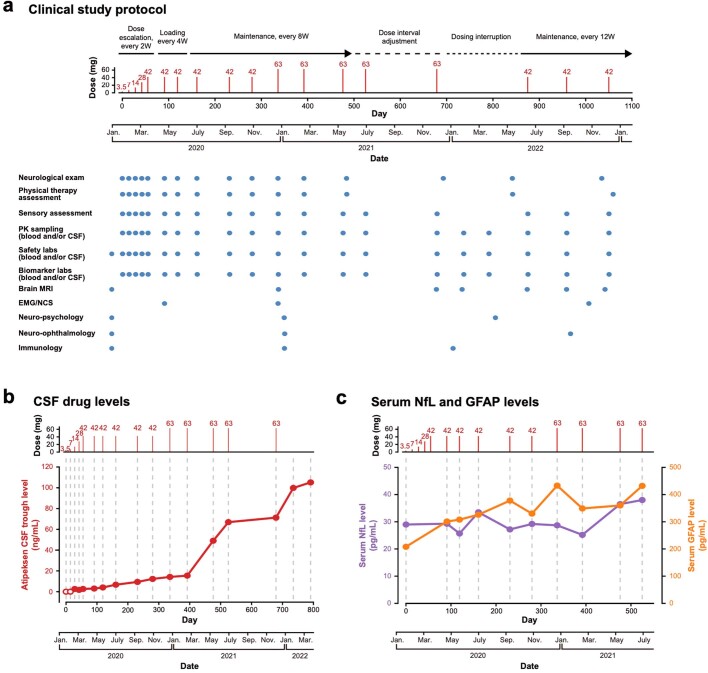
N-of-1 clinical study of AT008/atipeksen. **a**, Dosing schedule and clinical protocol. EMG, electromyography; NCS, nerve conduction study. **b**, Cerebrospinal fluid (CSF) drug levels. **c**, Neurofilament light chain (NfL) and GFAP levels in serum, which are biomarkers of neuronal cell death and neuroinflammation.

Following appropriate good manufacturing practice (GMP) and animal safety studies (previously described^1^), and in accordance with draft guidance from the US Food and Drug Administration (FDA; FDA-2020-D-2199, FDA-2021-D-0320, FDA-2021-D-1139 and FDA-2021-D-1140), the child was enrolled in an expanded access trial to determine whether atipeksen might prevent or slow disease progression. Intrathecal injection was chosen rather than intracerebroventricular injection after considering safety, pharmacokinetics and biodistribution, as outlined in Supplementary Note 8. Dosing was initiated at the age of two, with careful exploratory dose escalation followed by a maintenance regimen of 42 mg every three months (for dosing and assessments schedules, pharmacokinetic measurements, exploratory disease biomarkers and dose justification simulations, see Extended Data Fig. 12 and Supplementary Fig. 12). This is anticipated to be a multi-year trial, given that the most notable neurological decline in classic A-T occurs between the ages of 5 and 10 (refs. ^34–36^; at present, the patient is six years old). Investigational treatment has been well tolerated for three years so far with some encouraging signs (Supplementary Note 6).

## Discussion

ASOs exemplify a class of therapeutic agents suitable for developing variant-targeted therapies, including those applicable to as few individuals as a single patient^1^. Albeit promising, these opportunities also present challenges^1,6^. Our results begin to shed light on the generalizability and scalability of using ASOs to ameliorate the effect of splice-switching genetic variants.

In our cohort, we found that 9% of individuals with A-T had at least one variant probably amenable to intervention with splice-switching ASOs, and an additional 6% had possibly ASO-amenable variants, totalling 15% of the cohort. Notably, reaching this fraction required the use of WGS, as most splice-switching ASO-amenable variants (72%, 26/36) were located within deep intronic regions that would have been missed by exon-based sequencing. Furthermore, a substantial fraction of the ASO-amenable intronic variants (23%, 6/26) were not SNVs or short indels but rather SVs or mobile DNA element insertions, highlighting the need for comprehensive variant analytics. We note that upfront screening with RNA-seq in addition to WGS could be of additional benefit^37^ but could present some complexities (for example, logistical challenges collecting high-quality RNA samples) or other limitations (for example, the inability to assess genes not expressed in accessible tissues). Certainly, RNA-seq as a follow-up test is crucial for variants flagged as being potentially ASO-amenable.

These results provide a useful first estimate (9–15%) of the prevalence of this class of variants with potential therapeutic implications in the recessive genetic disorder A-T. Through a meta-analysis of published literature^38,39^, we also found that individuals with inherited retinal disease caused by recessive *ABCA4* deficiency (Stargardt disease or cone rod dystrophy) are likely to have ASO-amenable variants in similar proportions (Supplementary Note 7 and Supplementary Tables 15 and 16), leading us to suggest that around 15% is a reasonable first estimate for other recessive genetic conditions. Of course, conditions with unusual genetic architecture, such as spinal muscular atrophy (SMA), will pose exceptions: although SMA is caused by recessive variants in *SMN1*, all individuals with SMA can be treated via ASO- or small-molecule-mediated splice modulation^40,41^ because all patients also have an evolutionarily acquired, pathogenic splice ‘variant’ in *SMN2*, a gene that can substitute for *SMN1*. Another example is familial dysautonomia, in which almost all cases are associated with a founder variant that causes a tissue-specific splicing defect^42^ and may be amenable to splice modulation rescue^43^. With respect to dominant loss-of-function conditions, because these are associated with one pathogenic variant per individual, not two, one can expect the prevalence of splice-switching ASO-amenable variants to be roughly half (that is, about 5–8%).

Within the cohort of 235 individuals with A-T, we identified 35 individuals with ASO-amenable variants, and successfully conducted small-scale ASO screening for several probably amenable variants. ASOs successfully rescued mis-splicing for each of the tested variants, providing evidence of the utility of our predictive taxonomy. Splice-switching ASOs that are capable of rescuing functional *ATM* deficits in patient cell lines were successfully developed for two recurrent *ATM* variants. One of these is being tested in a pilot first-in-human investigational trial, which has been underway for the past 36 months.

We note that it might be possible to treat around 70% of these individuals with as few as 5 splice-switching ASOs, whereas treating all 35 individuals would require 15 distinct drugs (Extended Data Fig. 4b). This illustrates both the opportunities and the challenges of developing individualized splice-switching ASOs under a traditional ‘one-drug-at-a-time’ model. These opportunities and challenges will apply to other variant-specific treatment strategies (for instance, CRISPR editing), underscoring the need for innovative, platform-based scientific and regulatory approaches to unlock their potential.

Overall, our study provides a framework for how individualized genetic therapy might work, using genome sequencing and analysis to efficiently identify variants that might be amenable to splice-switching intervention, in time to design, test and deploy an appropriate ASO. If successful, these approaches could someday encourage a re-evaluation of which genetic findings are considered ‘actionable’. However, it is worth emphasizing that this approach at present remains investigational, and contains uncertainty and risk that have to be weighed carefully in a clinical context. Key logistical, regulatory, economic and ethical considerations remain as well^1,6,44,45^ (FDA draft guidance: FDA-2020-D-2199, FDA-2021-D-0320, FDA-2021-D-1139 and FDA-2021-D-1140). Continued study of patient-customized ASO therapies in serious genetic illness, facilitated by this framework, will be important to gather the body of evidence necessary to support the viability of this approach.

## Methods

### Patients

The WGS and clinical data of 235 patients with A-T were provided by the Global A-T Family Data Platform of ATCP. Our access to the data was approved by the Data Access Committee of ATCP.

Selected patients with A-T enrolled at the Manton Center for Orphan Disease Research under the approval of the Institutional Review Board (IRB) at Boston Children’s Hospital (10-02-0053). These included the individual with c.7865C>T who has been treated with AT008 (atipeksen), and individuals in the ATCP cohort, who were enrolled for WGS variant call validation by Sanger sequencing and mis-splicing validation by minigene assay and RNA-seq. gDNA samples extracted from the saliva of patients were provided by the Broad Institute. Whole-blood samples were provided by their physicians through the ATCP foundation, and RNA samples were extracted from these.

Functional studies using the cells derived from patients and their families were performed after obtaining appropriate consent under the auspices of an IRB-approved protocol maintained by the Manton Center for Orphan Disease Research Gene Discovery Core at Boston Children’s Hospital. Skin fibroblasts were derived from a 2-mm punch biopsy taken from the patient’s skin using explant culture. Fibroblasts were maintained and passaged in medium containing DMEM (Fisher Scientific) supplemented with 10% fetal bovine serum (Gibco). Fibroblasts used in experiments were under passage 20.

### Variant calling

WGS reads were aligned to GRCh38/hg38 using BWA (v.0.7.17) (ref. ^46^) and pre-processing and quality control were performed according to GATK Best Practice Workflows^12^. Multiple computational tools were used to call diverse types of variants, including GATK-HaplotypeCaller (v.3.5) (ref. ^12^), VarScan2 (v.2.4.4) (ref. ^13^) and Strelka2 (v.2.9.10) (ref. ^14^) for SNVs and short indels (less than 50 nt in length; Supplementary Table 2) and Delly (v.0.8.6) (ref. ^15^), Pindel (v.0.2.5b8) (ref. ^16^), MELT (v.2.2.2) (ref. ^17^), and xTea (v.0.1.7)^18^ for SVs (50 or more nt in length; Supplementary Table 3), with MELT and xTea used specifically for transposons. For large CNVs with imprecise boundaries, we manually inspected read alignments on Integrative Genome Viewer (IGV; v.2.8.9) (ref. ^47^) to determine rough boundaries of the variants.

### Relatedness

To analyse relatedness among the 235 individuals in the ATCP cohort, we used VCFtools (v.0.1.17) with the ‘relatedness2’ option^48^ (Supplementary Table 1), which is based on the KING software package^49^. For individuals with the annotated relatedness information in the clinical record, all annotations were consistent with the inferred relatedness.

### Variant effect prediction

For variant effect prediction, VEP (release 100)^50^ was used to annotate all SNVs and short indels. Protein-coding impact was evaluated using REVEL^21^ and experimental evidence of protein functionality in the literature (Supplementary Table 7); mis-splicing impact was evaluated using MaxEntScan^24^, SpliceAI^20^ and LaBranchoR^25^. LaBranchoR-predicted branchpoint coordinates on GRCh37/hg19 were downloaded. After converting them to the GRCh38/hg38 coordinates (using LiftOver), their potential overlap and distance to variants were examined. For variants shorter than 50 nucleotides, the allele frequencies were looked up in gnomAD (v.3.1) (ref. ^51^) and TOPMed (freeze 8; ref. ^52^). For SVs, the allele frequencies were looked up in dbVar^53^, DGV^54^ and gnomAD SVs (v.2.1) (ref. ^55^). The clinical significance of variants was looked up in ClinVar (as of 2 June 2020; ref. ^56^). All disease candidate SV events were confirmed by manually inspecting the raw sequencing data on IGV.

### ACMG classification

Disease candidate variants were classified using a five-tiered system in accordance with the guidelines outlined by ACMG^19,57^. For predicted loss-of-function variants, we used specialized ACMG recommendations to apply the PVS1 criteria^58^. For CNVs, we used a specialized scoring framework proposed by ACMG and Clinical Genome Resource (ClinGen)^59^.

### Determination of disease candidate variants

First, we defined SDVs. These include frameshift, stop-gain, start-loss and splice-site-destroying variants, and structural variants affecting one or more exons, as well as missense variants and short in-frame indels with previous experimental evidence of functional loss. Splice-site-destroying variants were defined as: (SpliceAI donor/acceptor loss score ≥ 0.1 at a canonical splice site) AND (MaxEntScan donor/acceptor score with the ALT allele < MaxEntScan donor/acceptor score with the REF allele) AND [(MaxEntScan donor/acceptor score with the ALT allele < 2) OR (MaxEntScan donor/acceptor score with the ALT allele < 0.3 × MaxEntScan donor/acceptor score with the REF allele)]. All SDVs were considered as disease candidate variants. Second, variants that were annotated as pathogenic or likely pathogenic in ClinVar were considered as disease candidate variants.

For the patients in whom fewer than two disease candidate events were identified in the previous two steps, we analysed the remaining variants in each patient on the basis of the population and cohort allele frequencies. We filtered out variant calls whose population or cohort allele frequencies are higher than that of c.5932G>T (p.Glu1978Ter); this variant has the highest allele frequency in this ATCP cohort among the variants annotated as pathogenic in ClinVar. It has gnomAD v.3.1 and ATCP cohort allele frequencies of 0.0000349045 and 0.034 (16/470), respectively. For the variant calls that had passed the allele frequency filter, their protein-coding and splicing impacts were examined on the basis of multiple computational tools: REVEL (for protein-coding impacts) and SpliceAI and MaxEntScan (for splicing impacts). Missense variants that were predicted as pathogenic by REVEL (score ≥ 0.5) were considered as disease candidate variants. Mis-splicing events with a SpliceAI score of 0.1 or higher were considered as likely true events. If the consequence of the mis-splicing is predicted to result in frameshift or loss of a crucial domain of the protein, the variant that caused the mis-splicing was classified as a disease candidate variant. For the patients in whom fewer than two disease candidate events were identified up to this step, we reviewed the remaining variants on a case-by-case basis (Supplementary Note 2).

Sanger sequencing validation of a subset of disease candidate variants was performed using available patient gDNA samples. The PCR protocol comprised 10 ng template DNA, 10 µl KAPA2G Robust HotStart ReadyMix (2X; Kapa Biosystems), 1 µl site-specific primer pairs (10 µM), and PCR-grade water to a final volume of 20 µl. The cycling parameters were 94 °C for 3 min; 30 cycles of 94 °C for 15 s, 60 °C for 15 s, 72 °C for 15 s; 72 °C for 3 min; and held at 4 °C. Validation primers are listed in Supplementary Table 4. All PCR amplicons were visualized on 2% agarose gels. Variants and corresponding genotypes were confirmed by Sanger sequencing (Supplementary Table 5).

### Phasing of disease candidate variants

#### Trio Sanger sequencing

Trio Sanger sequencing was performed on the family of the patient (with c.7865C>T) who has been under treatment with atipeksen, as well as on five individuals in the ATCP cohort (four families; DDP_ATCP_42 (with c.5763-1050A>G), DDP_ATCP_218, DDP_ATCP_38/39, DDP_ATCP_96). In all six cases, we confirmed with Sanger sequencing that the two disease candidate variants in each case are in *trans* (Supplementary Tables 1 and 6).

#### Homozygosity

In 32 cases (32 families), disease candidate variants were found to be homozygous. In five other cases (five families), disease candidate variants appeared homozygous owing to being in *trans* with a deletion at a locus overlapping the variants (Supplementary Table 1).

#### Read-based phasing

When the distance between two disease candidate variants is shorter than the read length, the two variants can be phased using read-based phasing methods. We used WhatsHap (v.1.0) (ref. ^23^), a read-based phasing tool, to analyse such cases, and found that in two cases (one family), the two disease candidate variants were in *trans*. These two variants were only 62 bp apart and were also confirmed by manual inspection of the raw sequencing data on IGV (Supplementary Table 1).

#### Variant co-occurrence

The gnomAD variant co-occurrence database can be used to predict that the two variants are likely to be in *cis* or in *trans*^60^. If two variants are in the same haplotype (that is, in *cis*), they tend to appear in the same individual. This analysis could be performed only for individuals whose two disease candidate variants are represented in the gnomAD database (v.2.1.1, in GRCh37/hg19 coordinates) at a global allele frequency of higher than 0% and less than 5%. A total of 47 individuals (38 families) in the ATCP cohort met these criteria. The analysis showed that 2 disease candidate variants are highly likely to be on different haplotypes in all of the 47 individuals (Supplementary Table 1).

### ASO amenability taxonomy

#### General rules

(1) If a variant damages both a canonical splice site and protein-coding function at the same time, more severe damage is considered as the representative damage of the variant. (2) Solid experimental evidence on mis-splicing or coding impact of a variant, if available, can override computational predictions. For a schematic illustration of the taxonomy, see Fig. 2.

#### Damage to canonical splicing

(1) Severe: (i) SpliceAI donor/acceptor loss score at a canonical splice site ≥ 0.1, (ii) MaxEntScan donor/acceptor score with the ALT allele at the site < MaxEntScan donor/acceptor score with the REF allele at the site, AND (iii) [MaxEntScan donor/acceptor score with the ALT allele at the site < 2] OR [MaxEntScan donor/acceptor score with the ALT allele at the site < 0.3 × MaxEntScan donor/acceptor score with the REF allele at the site].

(2) Moderate: (i) NOT severe (as defined above), (ii) SpliceAI donor/acceptor loss score at a canonical splice site ≥ 0.1, AND (iii) [MaxEntScan donor/acceptor score with the ALT allele at the site < MaxEntScan donor/acceptor score with the REF allele at the site, MaxEntScan donor/acceptor score with the ALT allele at the site ≥ 2, AND MaxEntScan donor/acceptor score with the ALT allele at the site ≥ 0.3 × MaxEntScan donor/acceptor score with the REF allele at the site] OR [The variant is ≤3 nt away from the LaBranchoR-predicted branchpoint OR the distance between the LaBranchoR-predicted branchpoint and the site is changed by >3 nt by the variant].

(3) No to little: NEITHER severe NOR moderate (as defined above).

#### Damage to protein-coding function

(1) Severe: (i) frameshift, stop-gain, or start-loss variant OR (ii) missense variant predicted as pathogenic by REVEL (score > 0.5).

(2) No to little: (i) NOT severe AND (ii) synonymous variant or missense variant predicted as benign by REVEL (score ≤ 0.5).

#### Mis-splicing type

(1) Gain of mis-splicing (gain): (i) SpliceAI donor/acceptor gain score at a non-canonical site ≥ 0.1 AND (ii) MaxEntScan donor/acceptor score with the ALT allele at the site ≥ 2.

(2) Exon skipping or intron retention (skipping or retention): SpliceAI donor/acceptor loss score at any canonical site ≥ 0.1 without an accompanying gain of mis-splicing by SpliceAI (donor/acceptor gain score < 0.1 at any non-canonical splice site).

(3) Neither: NEITHER gain, skipping, NOR retention.

### Minigene assay

#### Plasmid construction

To generate a minigene, we used the pSpliceExpress plasmid, which was a gift from S. Stamm (Addgene plasmid 32485; http://n2t.net/addgene:32485; RRID: Addgene_32485; ref. ^61^). The genomic fragment with a variant of interest was cloned into the pSpliceExpress donor vector using the BP recombination reaction. The inserted fragments for reference and alternative alleles were generated by a two-step PCR procedure. In the first round of PCR, the genomic region of interest was amplified from patient gDNA with attB tagged primers, which added 12 nucleotides of the attB1 and attB2 sites to the ends of amplicons. The second PCR reaction used the first PCR products as templates and extended them to contain complete attB sequences using universal adapter primer pairs. All PCR reactions were performed with Phusion Hot Start II DNA polymerase (Thermo Fisher Scientific) or PrimeSTAR GXL DNA polymerase (Takara Bio). Primer sequences used for minigene constructions were listed in Supplementary Table 10. Full attB PCR products were purified using the PureLink PCR Purification Kit or PureLink Quick Gel Extraction Kit (Invitrogen). Gateway BP Clonase II Enzyme Mix (Invitrogen) was used to recombine attB PCR products into pSpliceExpress. In brief, approximately 25 fmol (1 kb PCR product is 0.65 ng fmol^−1^) of purified attB PCR product was added to 75 ng of donor vector, TE buffer and 1 µl of BP Clonase Enzyme Mix to a final reaction volume of 5 µl. The reaction was incubated at room temperature for 1 h, after which 0.5 µl Proteinase K was added to stop the reaction. One microlitre of each BP Clonase reaction product was transformed into 25 µl OneShot TOP10 Chemically Competent *Escherichia coli* (Thermo Fisher Scientific). Transformed *E. coli* was spread on LB agar plates with ampicillin (1× LB agar with 50 µg ml^−1^ ampicillin) and incubated overnight at 37 °C. To screen for positive colonies containing the desired plasmids, a dozen colonies for each variant were picked up and diluted in 50 µl sterile water. Subsequently, colony PCRs were performed using Phusion Hot Start II DNA polymerase (Thermo Fisher Scientific), followed with 2% agarose gel inspection. The cycling programme was: bacteria were lysed and DNA was denatured at 98 °C for 10 min, followed by 30 cycles of 98 °C for 10 s, optimal annealing temperature for 20 s and 72 °C for 30 s, and final extension for 5 min at 72 °C. Primer sequences used for colony PCR are listed in Supplementary Table 10. Positive colonies were inoculated in liquid LB with ampicillin (1× LB and 50 µg ml^−1^ ampicillin) and were cultured in a shaking incubator at 275 rpm at 37 °C for 12–18 h. Plasmid DNA was extracted from overnight cultures using PureLink Quick Plasmid Miniprep Kit (Invitrogen) or ZR plasmid Miniprep Kit (Zymo Research). The genotypes and the sequences of plasmid inserts were confirmed by Sanger sequencing (Supplementary Table 11). At least one wild-type and one mutant plasmid were identified for each variant.

In some variants, full attB PCR products could not be amplified directly from patient gDNA owing to low quality or unavailability of the patient gDNA. In these cases, a wild-type fragment was amplified from human male gDNA (Promega) and used to construct reference plasmids as described above. The Q5 site-directed mutagenesis kit was used to introduce the variants into the reference plasmids (New England Biolabs). Twenty-five-microlitre PCR reactions were set up with mutagenic primers (Supplementary Table 10) and Q5 Hot Start High Fidelity 2X Master Mix to introduce the variant into the reference plasmids and amplify the mutant plasmids. The samples were denatured at 98 °C for 30 s and subjected to 25 cycles of 98 °C for 10 s, 50–72 °C (various annealing temperatures were tested) for 10 to 30 s, 72 °C for 20–30 s per kb, followed by a final extension at 72 °C for 2 min. The linear PCR products were ligated into the plasmid through DpnI restriction digestion and ligation. The mutant plasmids were transformed into competent *E. coli*. Single colonies were screened and inoculated in liquid LB and ampicillin. Plasmid DNA was collected from overnight cultures.

#### Splicing assay

Around 1 × 10^5^ HEK293T cells were seeded in 24-well plates. When the cells reached about 90% confluency, they were transfected using Lipofectamine 3000 (Thermo Fisher Scientific). For each transfection, 4 µl of plasmid was added to each well along with 1.5 µl Lipofectamine, 2 µl P3000 and 50 µl Opti-MEM (Thermo Fisher Scientific). For some transfections, ASOs were also added at a final concentration of 200 µM. Twenty-four hours after transfection, total RNA was extracted using the PureLink RNA Mini Kit (Invitrogen). RNA was then reverse-transcribed into cDNA in a 4-μl total reaction consisting of 3 µl RNA and 1 µl of SuperScript IV VILO Master Mix (Thermo Fisher Scientific). The reverse transcription reactions were incubated at 25 °C for 10 min, 50 °C for 10 min and 85 °C for 5 min. To detect transcripts transcribed from the transfected plasmids, 1 µl cDNA was amplified using Phusion Hot Start II DNA polymerase (Thermo Fisher Scientific), 2× KAPA SYBR Fast qPCR Master Mix (Kapa Biosystems) or 2× KAPA HiFi HotStart ReadyMix (Kapa Biosystems). For primers, we used rat insulin primers that bind to the minigene exons flanking the inserted *ATM* gene region (Supplementary Table 10). The final PCR products were run and visualized on 2% agarose gel. Mis-splicing bands were extracted using the PureLink Quick Gel Extraction (Invitrogen) and confirmed by Sanger sequencing (Supplementary Table 12).

#### Quality control

If the amount of the canonical splicing isoform represented less than 50% of the total amount of all *ATM* isoforms, we disqualified and excluded the minigene assay plasmids for further analysis. We found that some of the plasmids bearing the *ATM* gene region did not express the normally spliced isoform even without any variant, which makes them unsuitable to assess the mis-splicing effects of variants. Therefore, we excluded them from the analysis. The minigene assay plasmids carrying the *ATM* gene contexts of two variants (c.3489C>T [in DDP_ATCP_138] and c.4801A>G [in DDP_ATCP_302]) did not pass this criterion as they showed predominant skipping of the exon of interest even in the absence of the variant of interest in the *ATM* gene region of the plasmids.

### ASO development

#### ASOs

For c.7865C>T, a total of 32 ASOs were designed (12 for the initial screening and 20 for the fine-tuning screening). The ASOs were designed to be complementary to either the region encompassing the novel splice donor site in exon 53 created by c.7865C>T or predicted splice silencers surrounding the exon 53 canonical splice donor site. These silencers were predicted on the basis of a previously published hexamer-based model^62^. For c.5763-1050A>G, a total of 27 ASOs were designed (12 for the initial screening and 15 for the fine-tuning screening) to be complementary to the regions encompassing the novel splice donor site in intron 38 created by c.5763-1050A>G, the cryptic acceptor site of the pseudoexon in intron 38 or predicted splice silencers within the pseudoexon (also predicted on the basis of the hexamer model). For minigene-based validation of ASO amenability, a total of 24 ASOs were designed for 4 ASO-amenable variants (c.2839-579_2839-576del, c.2839-581G>A, c.6348-986G>T and c.3994-159A>G). The ASOs were designed to block either the splice donor/acceptor site or predicted exonic splicing silencers within a pseudoexon of interest. NT-20 and NT-22 (non-targeting oligonucleotides with the same chemistry) were used as negative controls^1^. For in vitro toxicity testing, ASO-tox, a gapmer with known toxicity, was used. All ASO sequences and detailed chemical modifications of ASOs are provided in Supplementary Table 13. All ASOs were manufactured by Microsynth. The ASO drug substance used in the atipeksen N-of-1 clinical trial was manufactured by ChemGenes in accordance with GMP guidelines.

#### ASO screening

Fibroblasts were transfected with 200 nM ASOs using Lipofectamine 3000 (Thermo Fisher Scientific). Twenty-four hours after transfection, total RNA was isolated using PureLink RNA Mini (Invitrogen). cDNA synthesis using oligo-dT and random hexamers was performed using the Superscript VILO reverse transcriptase kit (Invitrogen). For allele-specific PCR, primers were designed to specifically exclude the non-target allele in each patient (Extended Data Figs. 6d and 9c and Supplementary Table 14). For c.5763-1050A>G, the distance between the two *ATM* variants was too far (around 2 kb) to distinguish the two bands representing normally and abnormally spliced products (which differ by 137 bp) on a agarose gel; therefore, a nested PCR was performed. PCR was performed using 1 µl of cDNA and a standard condition (35 cycles; 98 °C for 5 s, 60 °C for 15 s, 72 °C for 45 s). Relative quantities of the normally and abnormally spliced transcripts were measured by 1.5% agarose gel electrophoresis and densitometry analysis using ImageJ.

### ASO validation

#### Immunoblotting

Fibroblasts were transfected with 400 nM ASO as described above. Forty-eight hours after transfection, cells were irradiated with 10 Gy using a caesium-137 source, and then incubated for 30 min at 37 °C. Cell lysates were then collected using RIPA buffer (Boston Bioproducts) supplemented with Roche PhosSTOP (Sigma-Aldrich). Lysates were incubated with 4× Laemmli buffer (BioRad) and loaded onto 4–15% precast gradient protein gels (BioRad) and separated by electrophoresis. Protein samples were then transferred to PVDF membranes, which were subsequently incubated overnight with primary antibodies for phospho-P53 (Cell Signaling Tech, diluted 1:500) and phospho-KAP1 (Bethyl Lab, diluted 1:1,000). GAPDH was used as a loading control and primary antibody for GAPDH (Proteintech) was diluted to 1:250. Following incubation with secondary antibodies that were diluted to 1:5,000 for phospho-P53, phospho-KAP1 and GAPDH (Li-Cor), targets were visualized with the Li-Cor Odyssey system and quantified with densitometry analysis (ImageJ).

#### Immunocytochemistry

Fibroblasts were transfected with 200 nM of ASOs as described above. Forty-eight hours after transfection, cells were irradiated with 1.5 Gy using a caesium-137 source, and then incubated for 60 min at 37 °C. Cells were washed in PBS, fixed in 4% (w/v) paraformaldehyde and permeabilized with 0.1% (w/v) Triton X-100 in PBS at room temperature. Cells were then incubated overnight in PBS with 3% BSA and antibodies to phospho-P53 (Cell Signaling Tech) and phospho-KAP1 (Bethyl Lab) and were visualized with immunoglobulin G Alexa Fluor conjugates (Life Technologies). DNA was counterstained with Hoechst 33342. Images were collected with the ImageXpress Micro microscope (Molecular Devices) and processed with MetaXpress (Molecular Devices). The abundance of targets expressed in nuclei was quantified.

#### Dose–response

Fibroblasts were electroporated using the Neon Transfection System (Thermo Fisher Scientific) with varying amounts of ASOs: 0–1,000 nM (0, 1, 2, 5, 10, 20, 50, 100, 200, 500, 1,000 nM; final concentrations). Twenty-four hours after electroporation, total RNA was isolated as described above. cDNA synthesis, RT–PCR, gel electrophoresis and densitometry were performed as described above.

#### RNA-seq

Fibroblasts were transfected with 200 nM of ASOs as described above. Forty-eight hours after transfection, total RNA was isolated as described above. RNA-seq libraries were prepared using the KAPA Hyper Prep kit (KAPA Biosystems). Sequencing was performed on an Illumina HiSeq 2500 (for sequencing; 2 × 100 bp). For alignment, STAR (v.2.7.5c) (ref. ^63^) was used to map reads on GRCh38/hg38 in the paired-end, two-pass mode to yield BAM files that were sorted by chromosomal coordinates. Gene annotation was not provided to the alignment program to avoid any biased alignment favouring annotated splice junctions. The sorted BAM files were indexed using SAMtools (v.1.10) (ref. ^64^). IGV was used to draw sashimi plots, which showed the number of reads supporting splice junctions.

#### Off-target analysis

The following derivative sequences were computationally generated from the sequences of AT008 (atipeksen), AT026, AT056, nusinersen and milasen: (1) sequences with progressively trimmed ends, starting from the full-length ASO sequences down to 16 nt in length; (2) sequences with up to 2 nt mismatches; and (3) sequences with a 1-nt internal insertion or deletion (Supplementary Figs. 5–8 and 11). BWA (v.0.7.17) (ref. ^46^) was used to align the generated sequences on GRCh38/hg38 and the RefSeq transcriptome sequences, downloaded from the UCSC Genome Browser.

#### In vitro ASO toxicity assay

An FITC Annexin V Apoptosis Detection Kit I (BD 556547, BD Biosciences) was used to quantitatively measure the percentage of cells undergoing apoptosis after transfection with ASOs at different concentrations as described above. Cells were collected, washed with PBS and resuspended in 1× binding buffer four days after transfection. Five hundred microlitres of the resuspended cells was stained with 5 µl of Annexin V-FITC and 5 µl propidium iodide (PI) in the dark at room temperature for 15 min. The cells were analysed using a flow cytometer (BD FACSAria III system) and were quantified by FlowJo software. The Annexin-V-positive and PI-negative fraction was ‘early apoptotic’, and the Annexin-V-positive and PI-positive fraction was ‘late apoptotic or necrotic’.

### Reporting summary

Further information on research design is available in the Nature Portfolio Reporting Summary linked to this article.

## Online content

Any methods, additional references, Nature Portfolio reporting summaries, source data, extended data, supplementary information, acknowledgements, peer review information; details of author contributions and competing interests; and statements of data and code availability are available at 10.1038/s41586-023-06277-0.

### Supplementary information

Supplementary Figure 1 Unprocessed gel and blot images.

Reporting Summary

Supplementary Information This file contains Supplementary Notes, Supplementary Figures 2-12, Supplementary Tables 5, 6 and 11, and Supplementary References.

Supplementary Tables This file contains Supplementary Tables 1-4, 7-10, and 12-16.

## Figures and Tables

**Extended Data Table 1:**
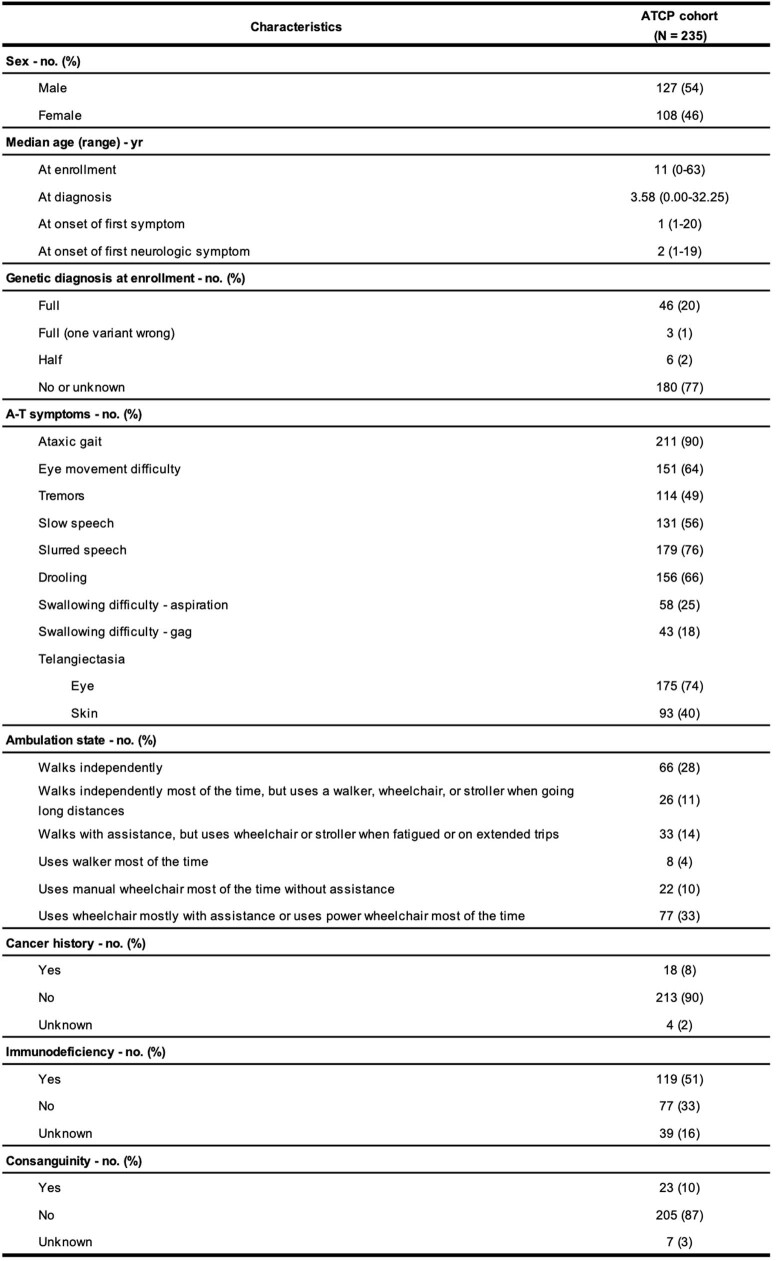
Cohort summary

Clinical and demographic characteristics of 235 individuals with A-T in the ATCP cohort. All the information is as of the time of enrolment and as reported by the patients, their families and/or clinicians. AFP, alpha-fetoprotein.

## Data Availability

Raw WGS data for the ATCP cohort are available from the Global A-T Family Data Platform (https://atfamilies.org) through the Terra cloud server after obtaining approval from ATCP. Raw RNA-seq data from the patients and patient-derived cells described in this study are available through Zenodo (10.5281/zenodo.7783848) upon request to investigators conducting IRB-approved research. The following public datasets were used: gnomAD v.3.1 and gnomAD SVs v.2.1 (https://gnomad.broadinstitute.org/), TOPMed freeze 8 (https://bravo.sph.umich.edu/freeze8/hg38/), dbVar (https://www.ncbi.nlm.nih.gov/dbvar/), DGV (http://dgv.tcag.ca/dgv/app/home) and ClinVar (https://www.ncbi.nlm.nih.gov/clinvar/).
