# Supplementary material for: A framework for individualized splice-switching oligonucleotide therapy
Source: Nature. 2023 Jul 12;619(7971):828–36. doi: 10.1038/s41586-023-06277-0 (PMC10371869; doi:10.1038/s41586-023-06277-0)
Supplement: Supplementary file 1 — Unprocessed gel and blot images. [file 41586_2023_6277_MOESM1_ESM.pdf]

nature portfolio

https://doi.org/10.1038/s41586-023-06277-0

Supplementary information

A framework for individualized splice-switching oligonucleotide therapy

In the format provided by the authors and unedited

Supplementary Fig. 1 (page 1/18)

Fig. 4

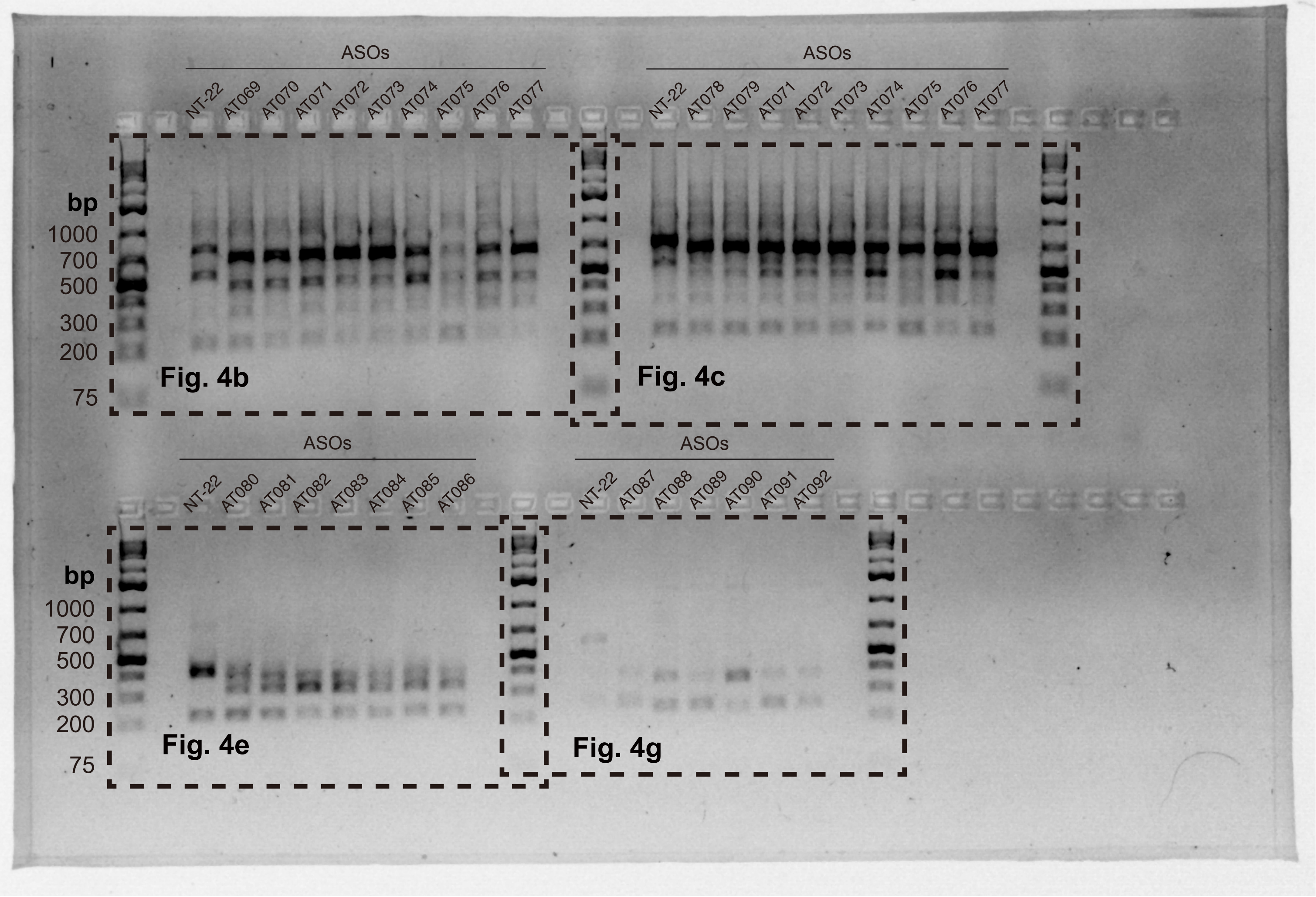

Supplementary Fig. 1 (page 2/18)

Fig. 5a/Extended Data Figs. 6d,7b

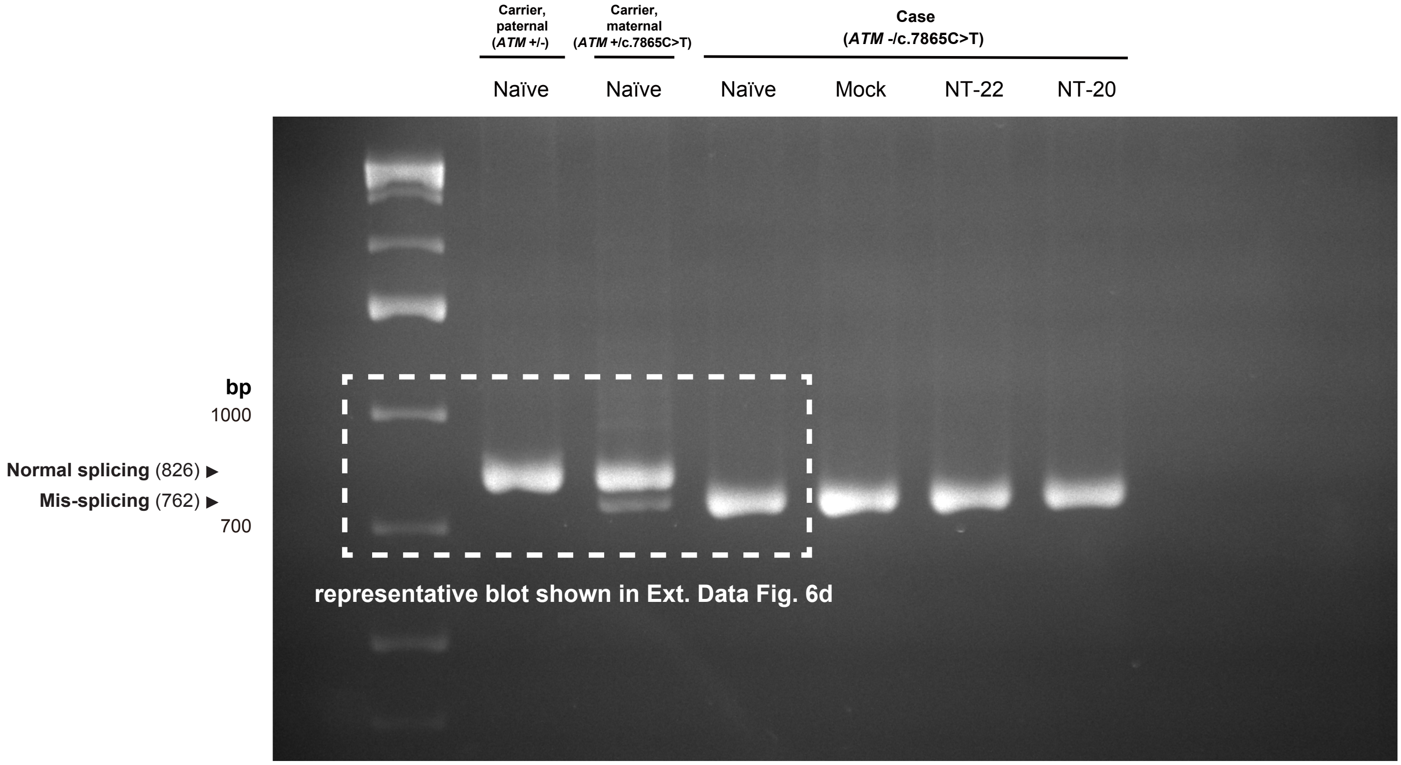

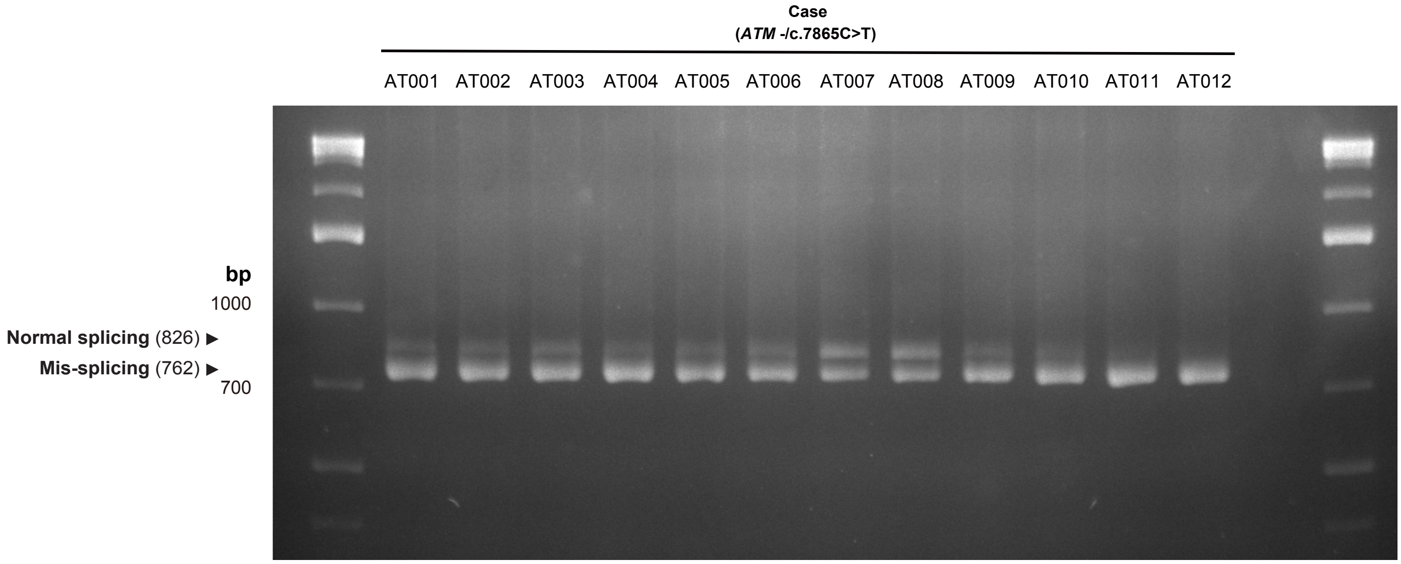

Supplementary Fig. 1 (page 3/18)

Fig. 5a/Extended Data Fig. 7b

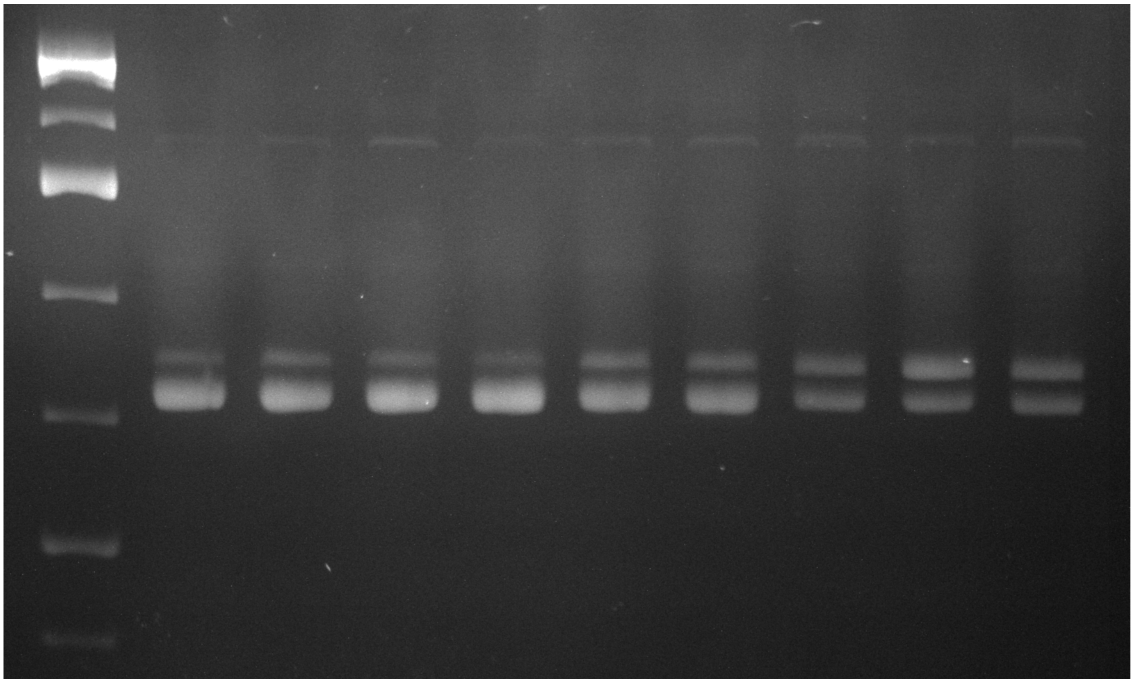

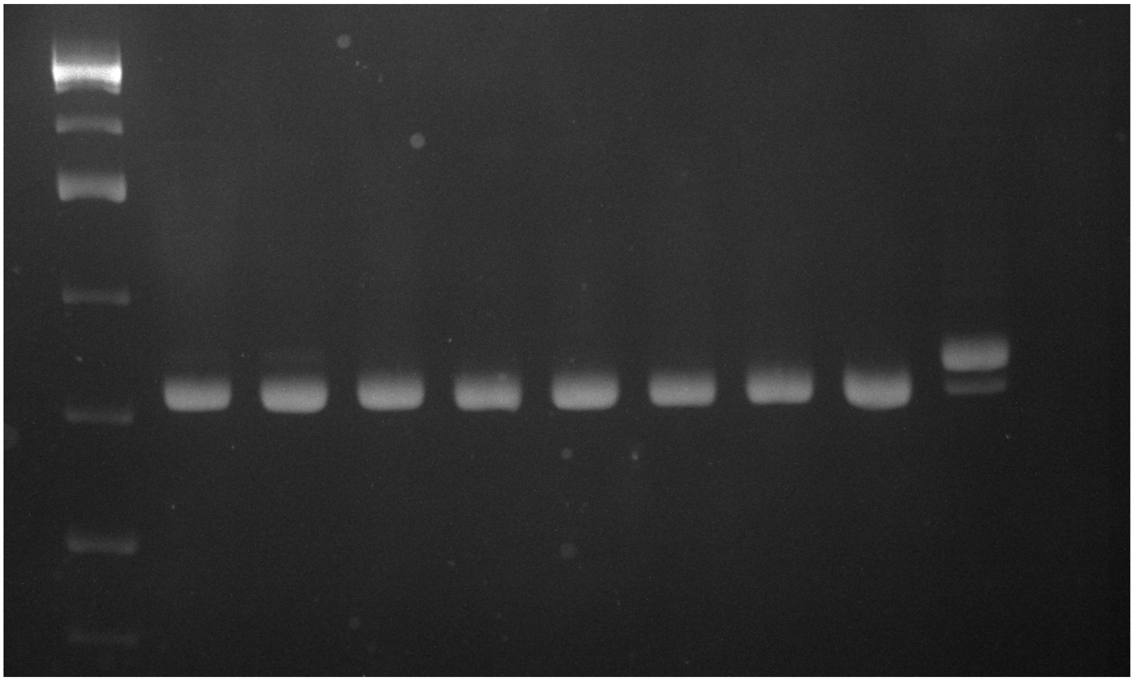

Supplementary Fig. 1 (page 4/18)

Fig. 5a/Extended Data Fig. 7b

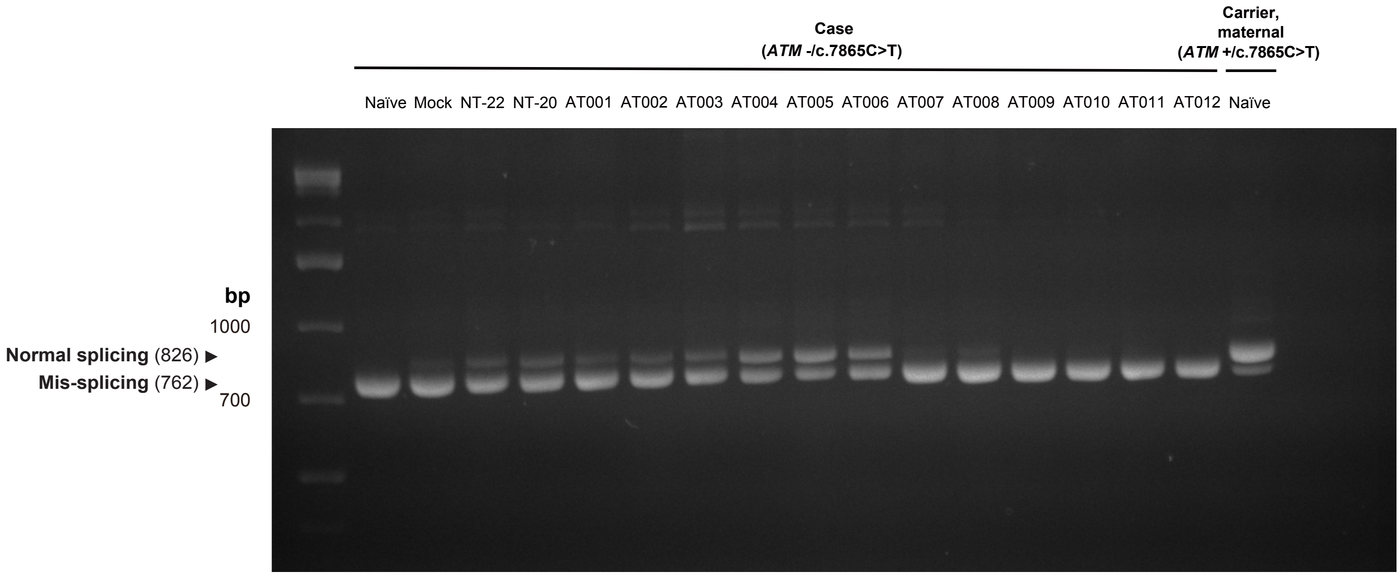

Supplementary Fig. 1 (page 5/18)

Fig. 5a/Extended Data Fig. 7b

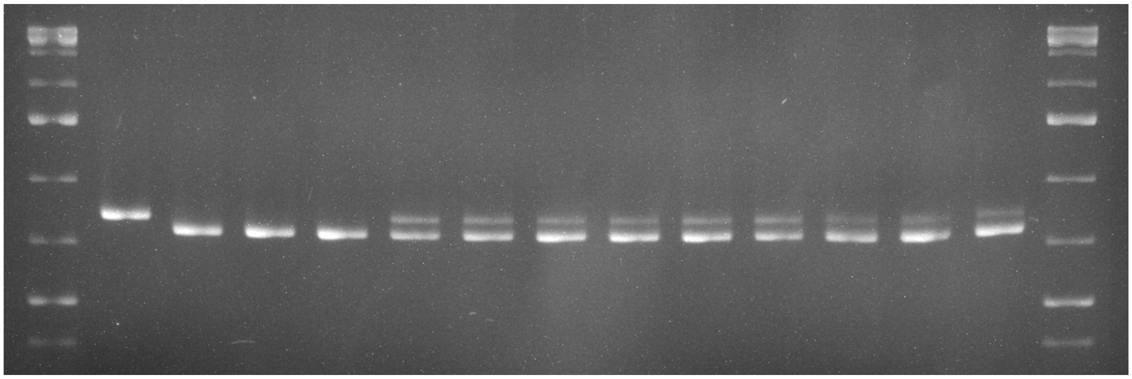

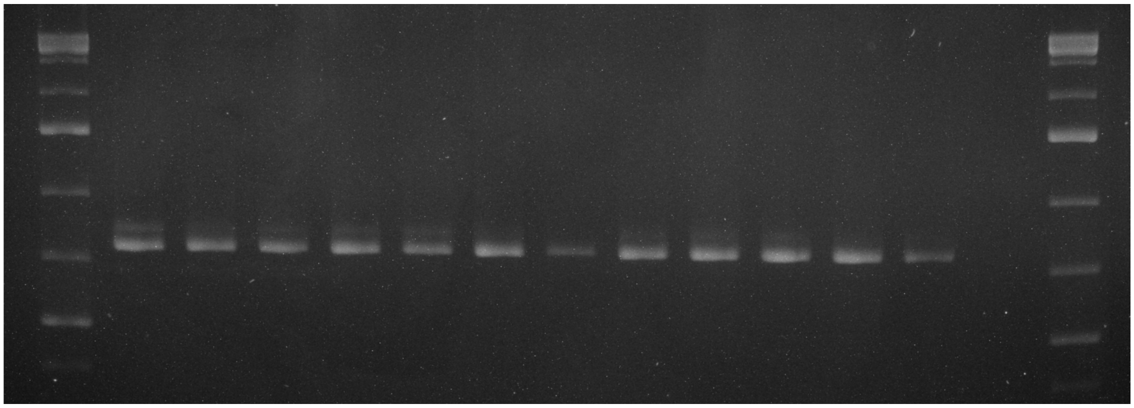

Supplementary Fig. 1 (page 6/18)

Fig. 5a/Extended Data Fig. 7b

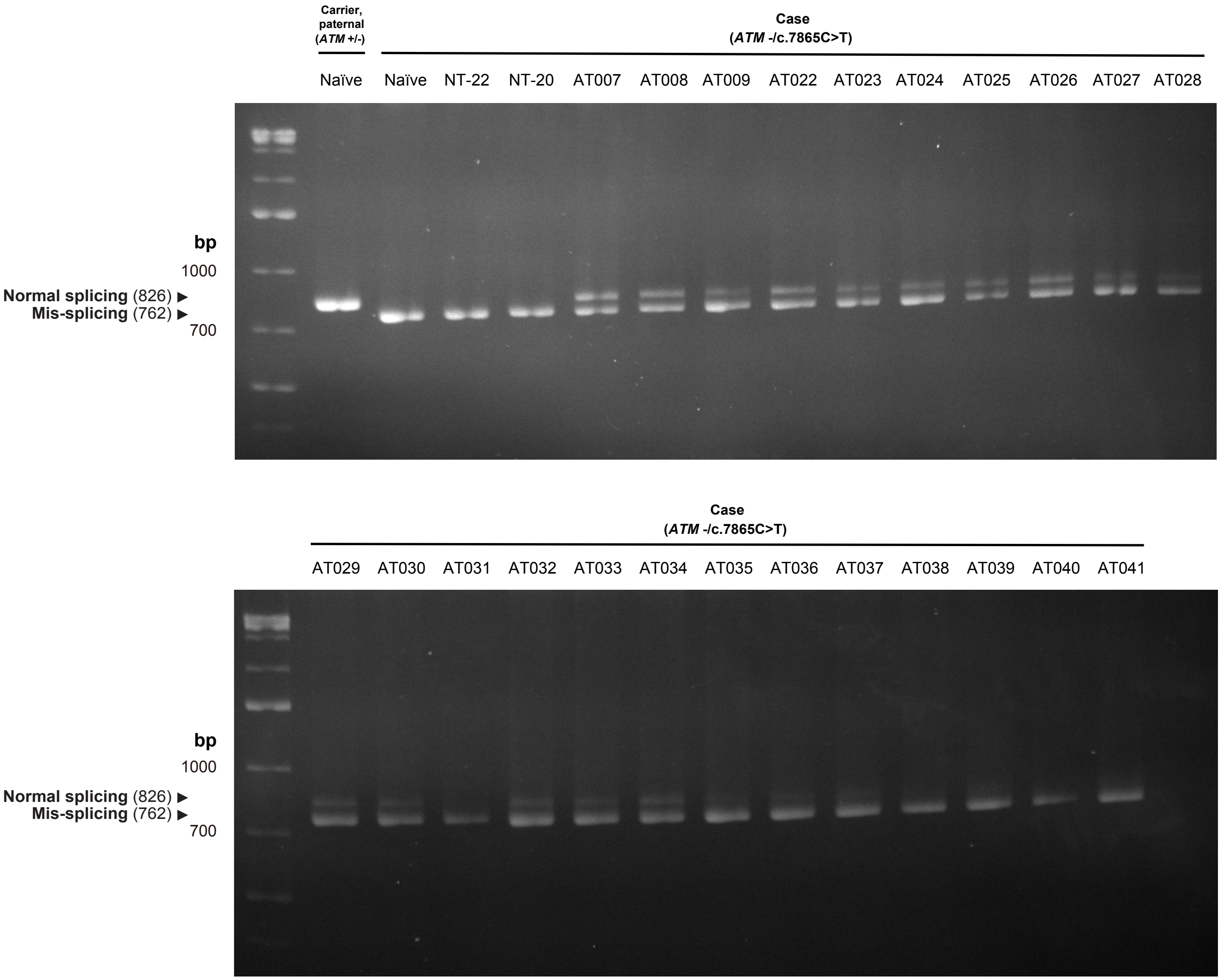

Supplementary Fig. 1 (page 7/18)

Fig. 5b/Extended Data Fig. 8a

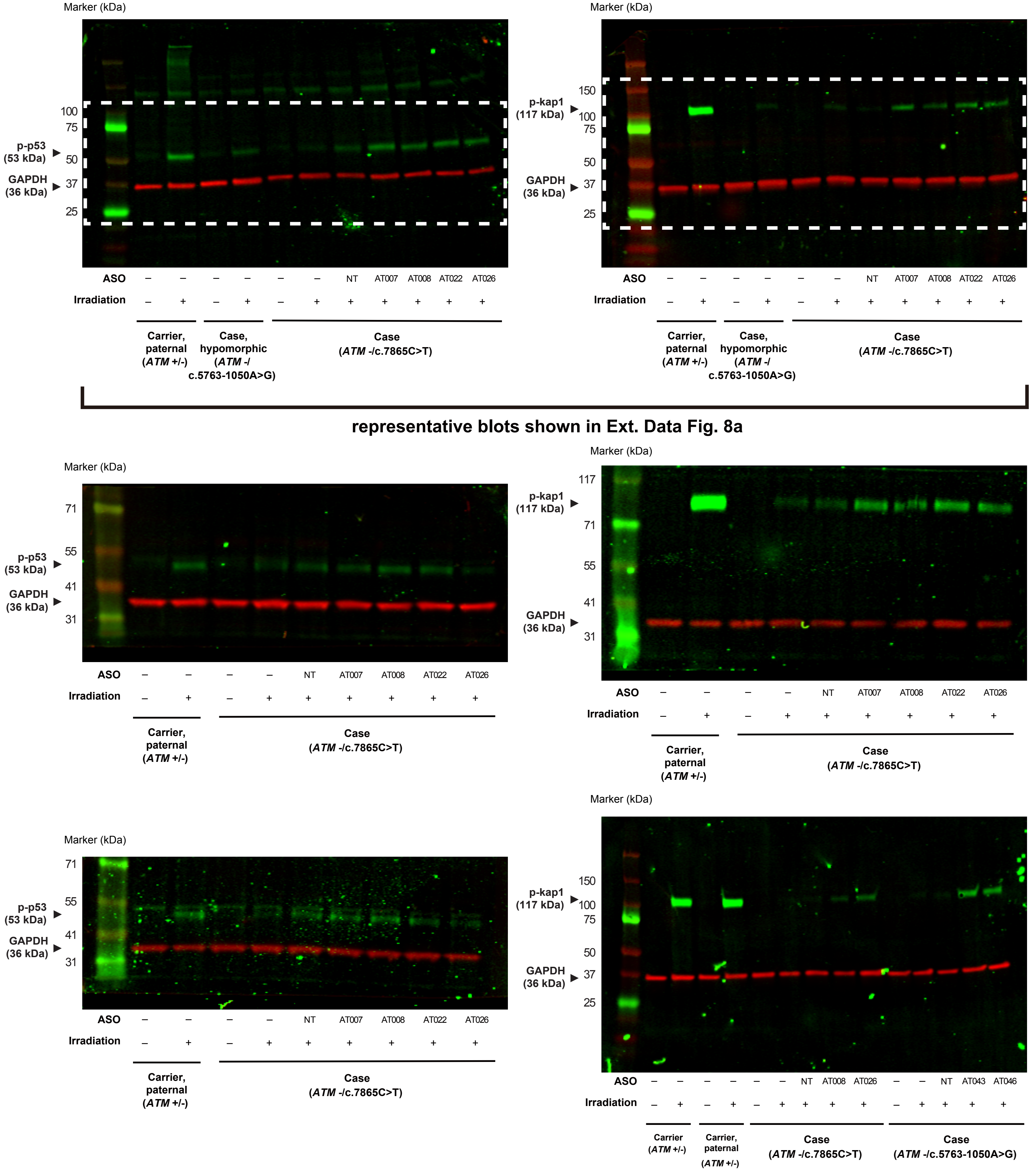

Supplementary Fig. 1 (page 8/18)

Fig. 5b/Extended Data Fig. 8a

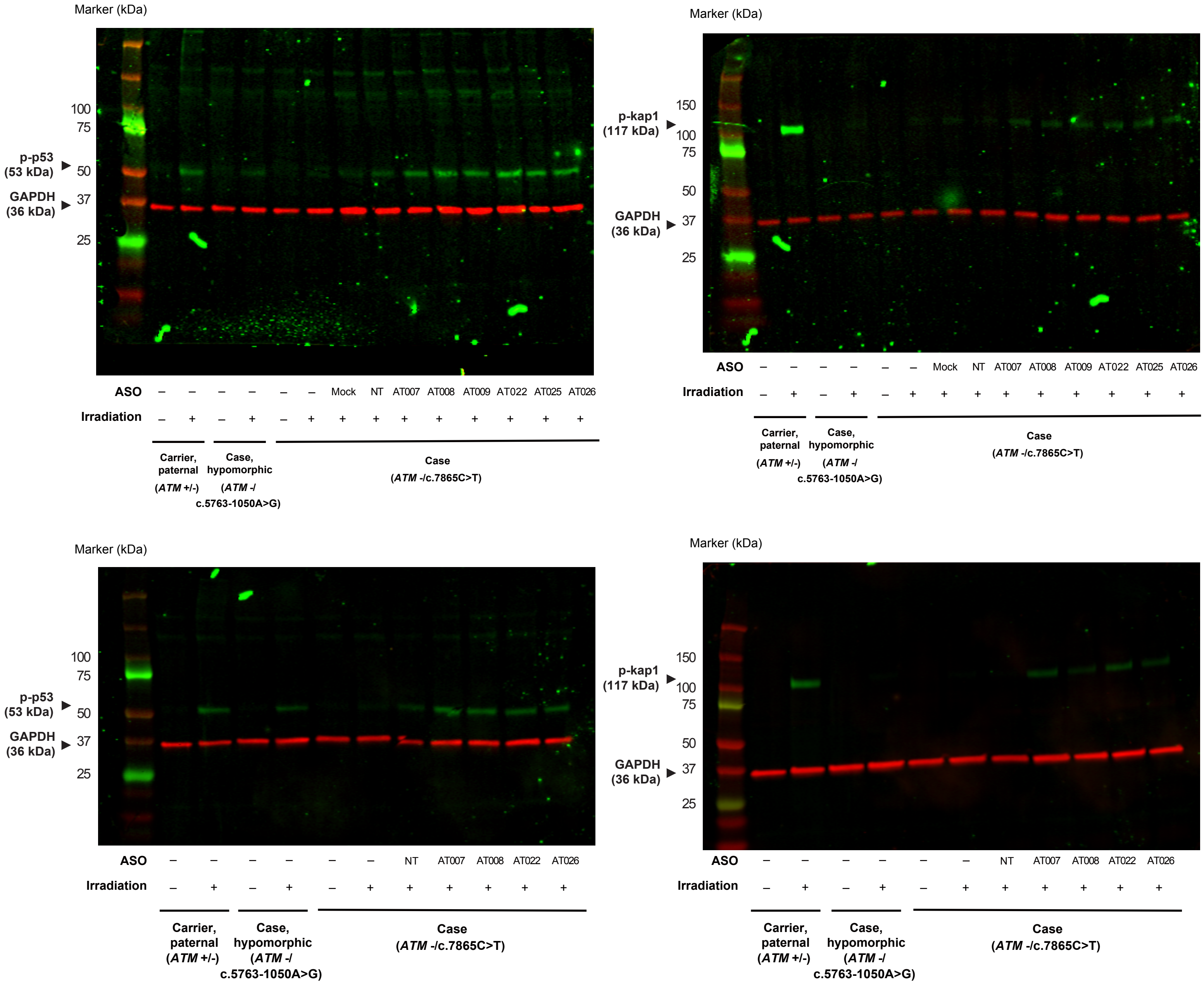

Supplementary Fig. 1 (page 9/18)

Extended Data Fig. 5

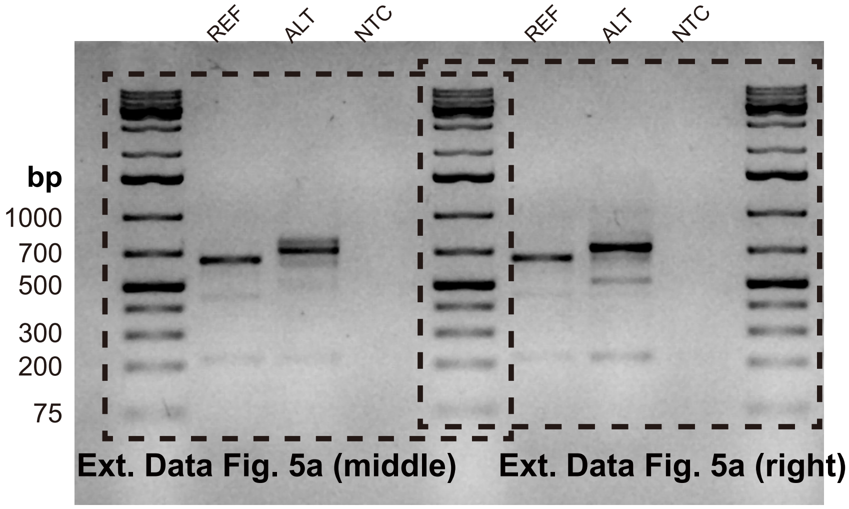

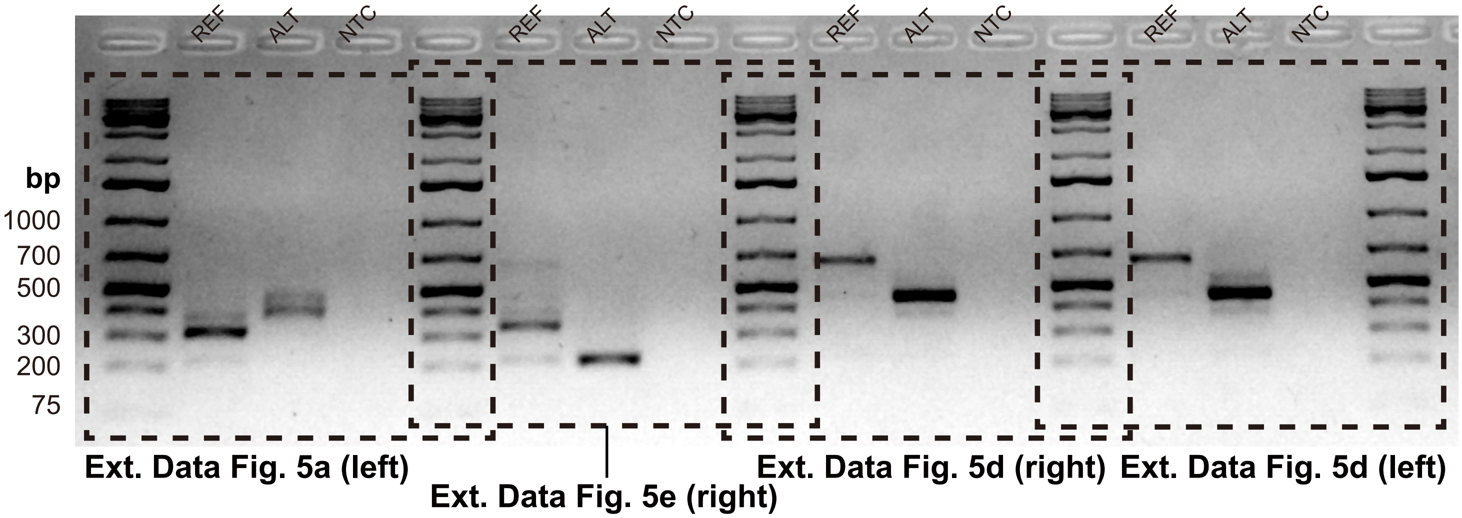

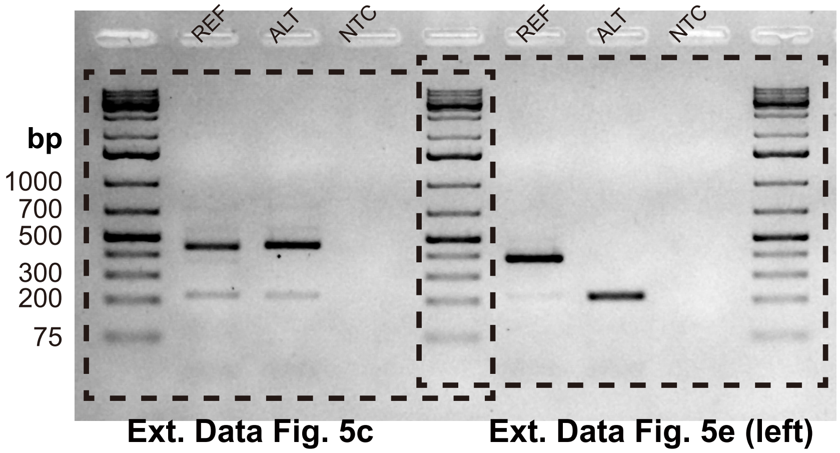

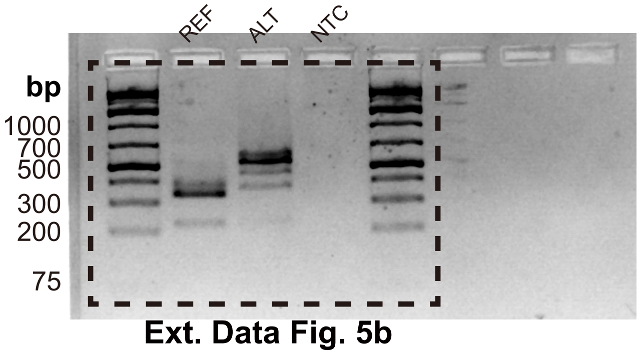

Supplementary Fig. 1 (page 10/18)

Extended Data Fig. 8c - AT008

NTC Naïve NT-20 NT-22 0 1 2 5 10 20 50 100 200 500 1000

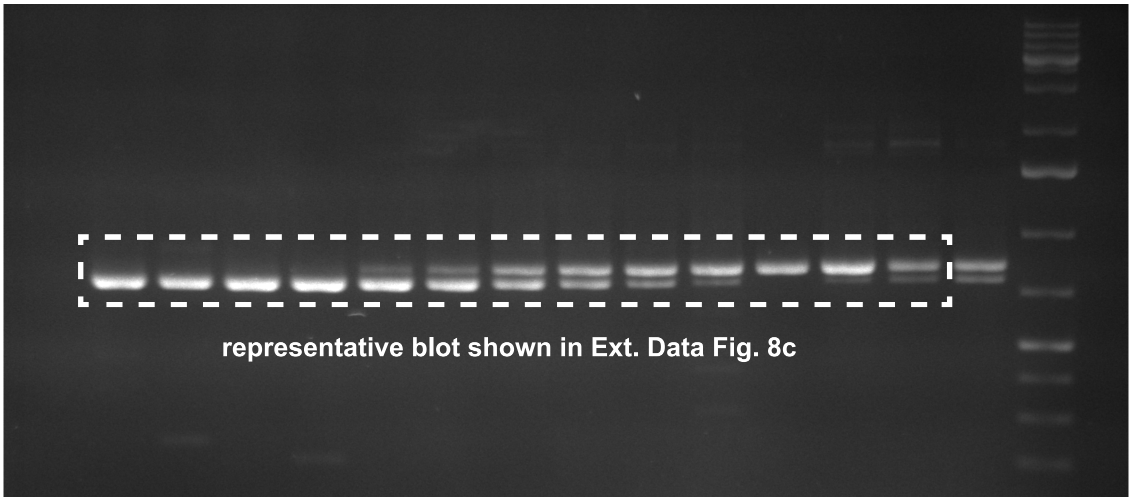

bp
1000
◄ **Normal splicing** (826)
◄ **Mis-splicing** (762)
700

NTC Naïve NT-20 NT-22 0 1 2 5 10 20 50 100 200 500 1000

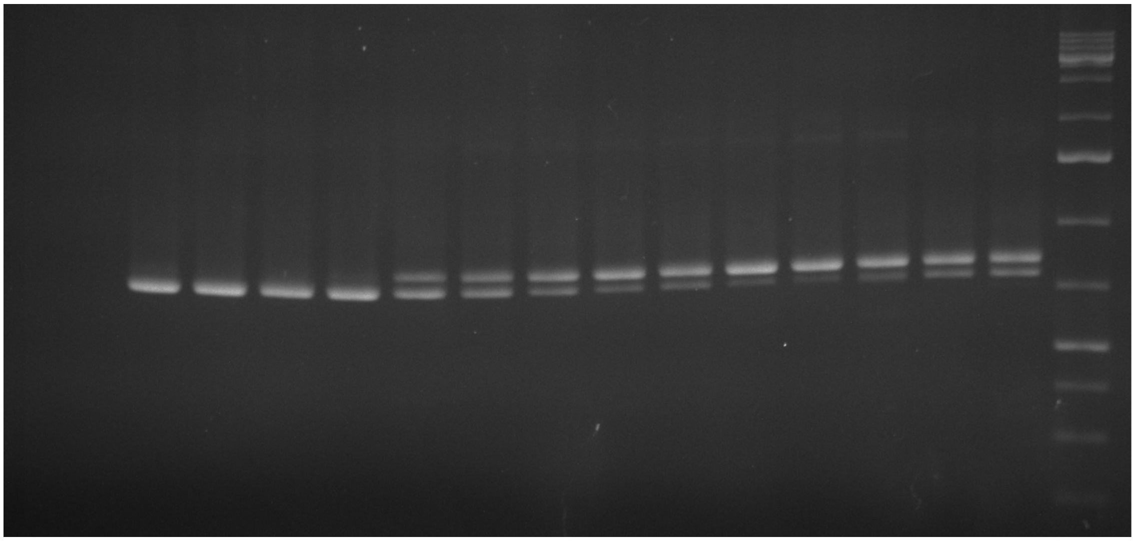

bp
1000
◄ **Normal splicing** (826)
◄ **Mis-splicing** (762)
700

NTC Naïve NT-20 NT-22 0 1 2 5 10 20 50 100 200 500 1000

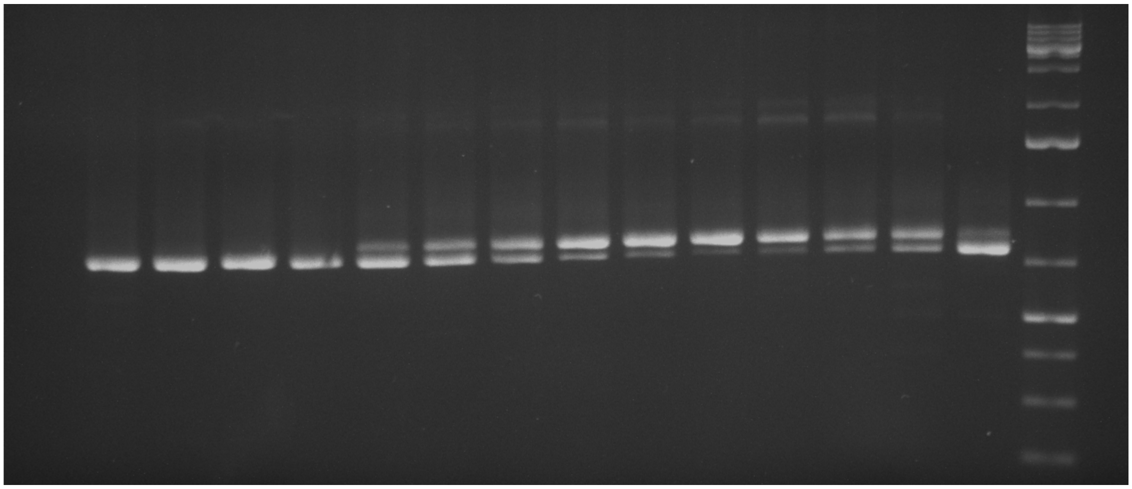

bp
1000
◄ **Normal splicing** (826)
◄ **Mis-splicing** (762)
700

Supplementary Fig. 1 (page 11/18)

Extended Data Fig. 8c - AT026

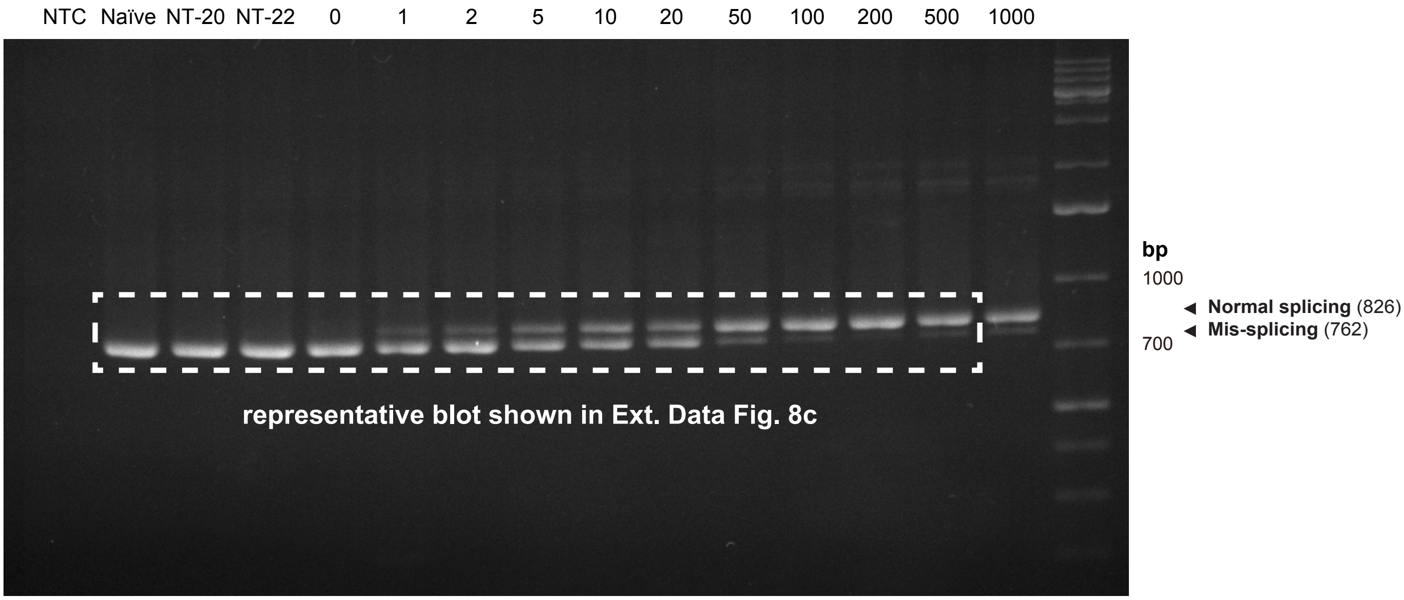

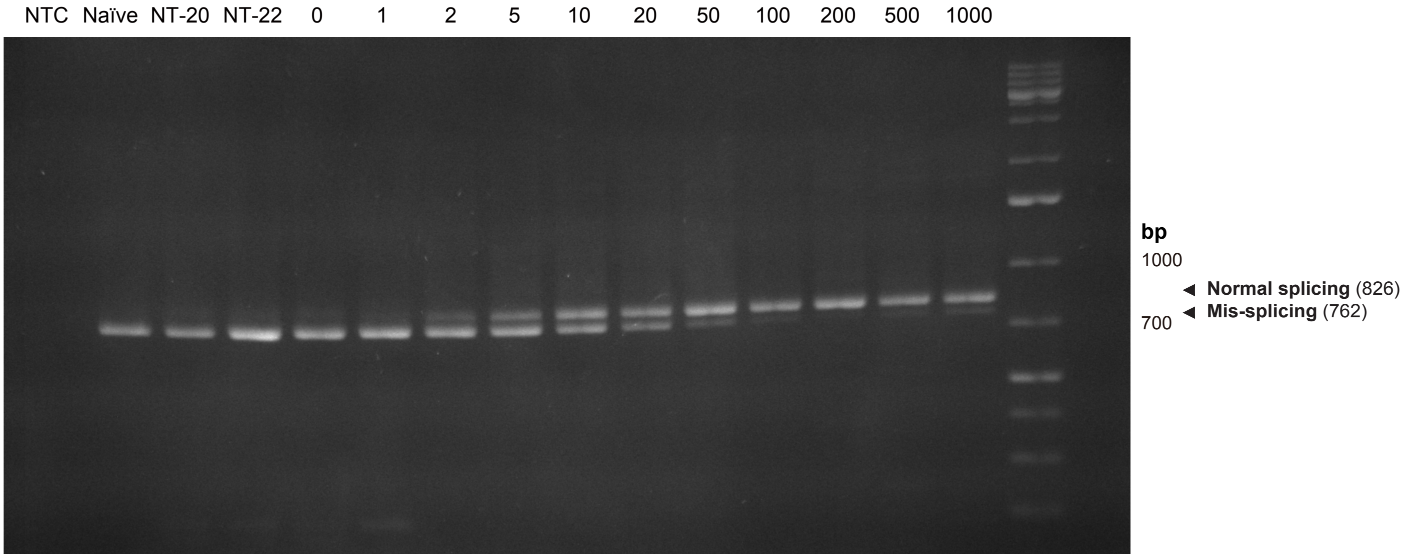

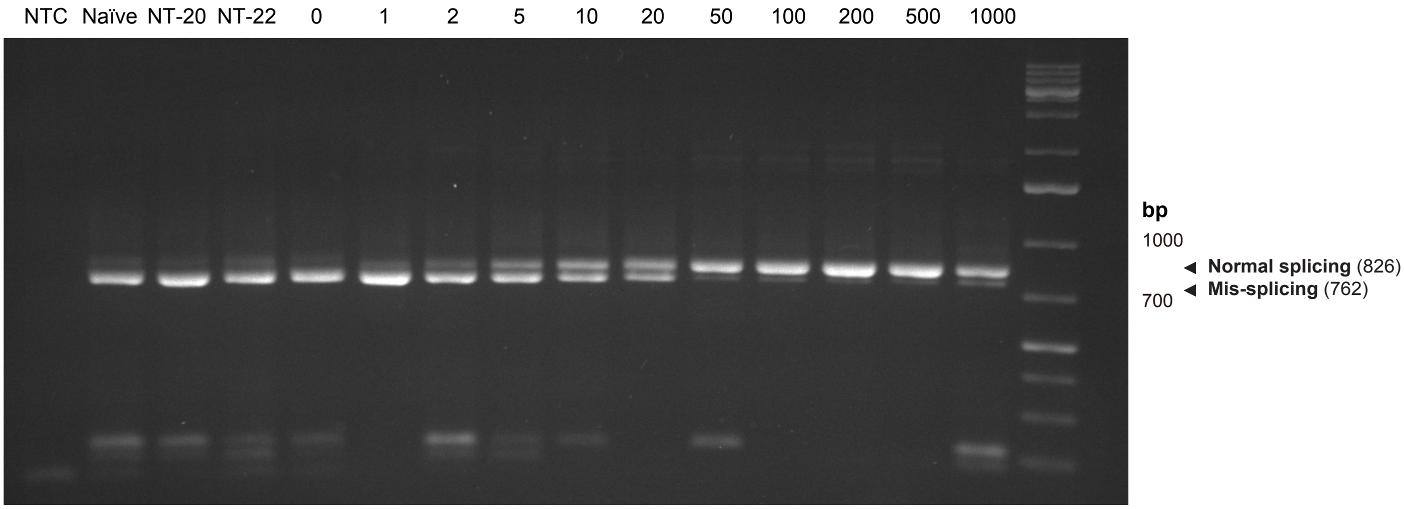

Supplementary Fig. 1 (page 12/18)

Extended Data Figs. 9c,10

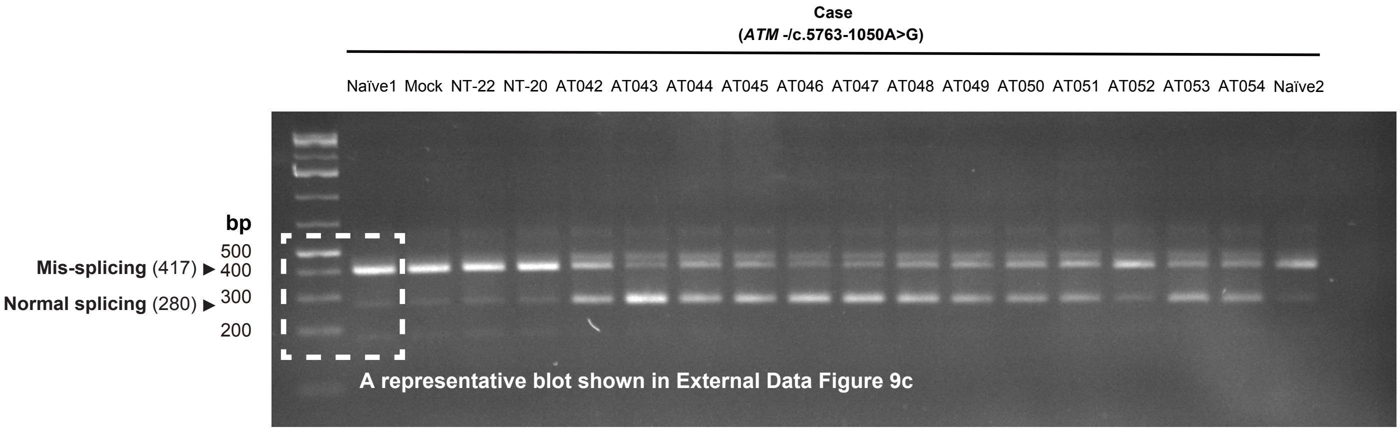

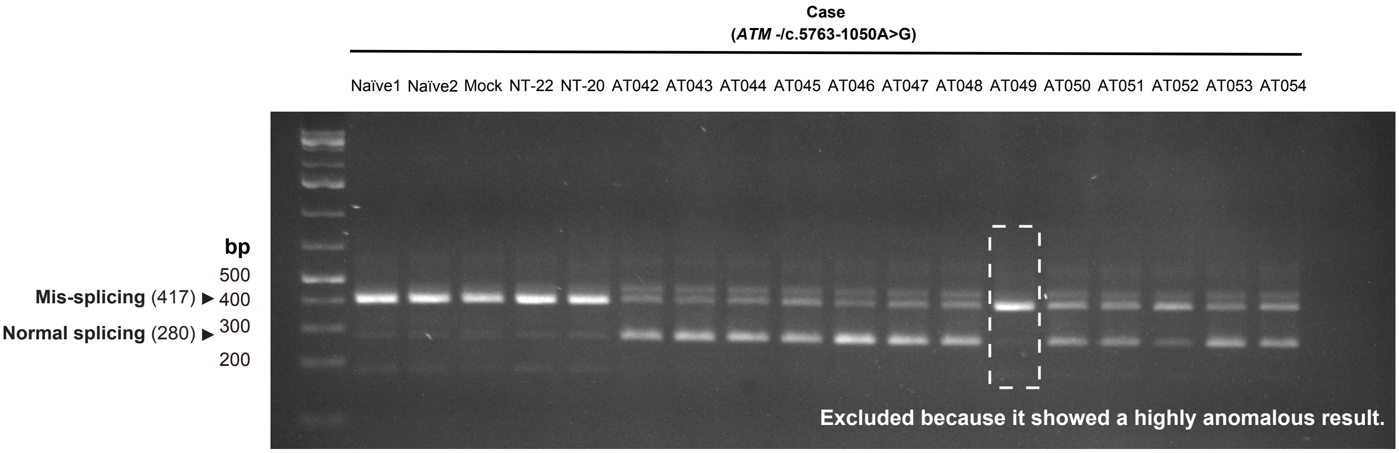

Supplementary Fig. 1 (page 13/18)

Extended Data Fig. 10

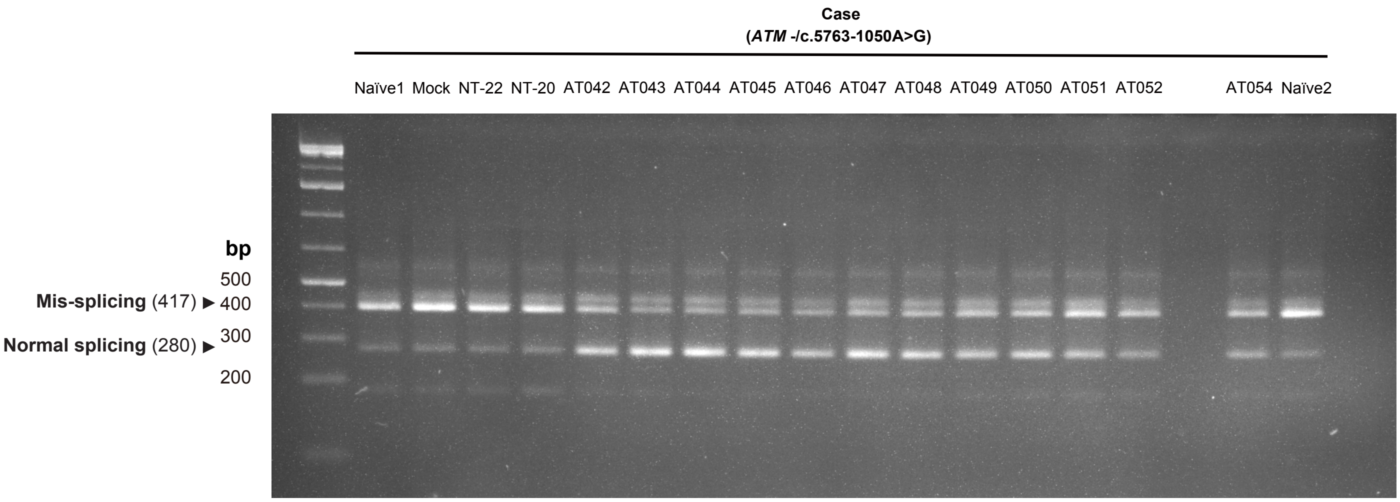

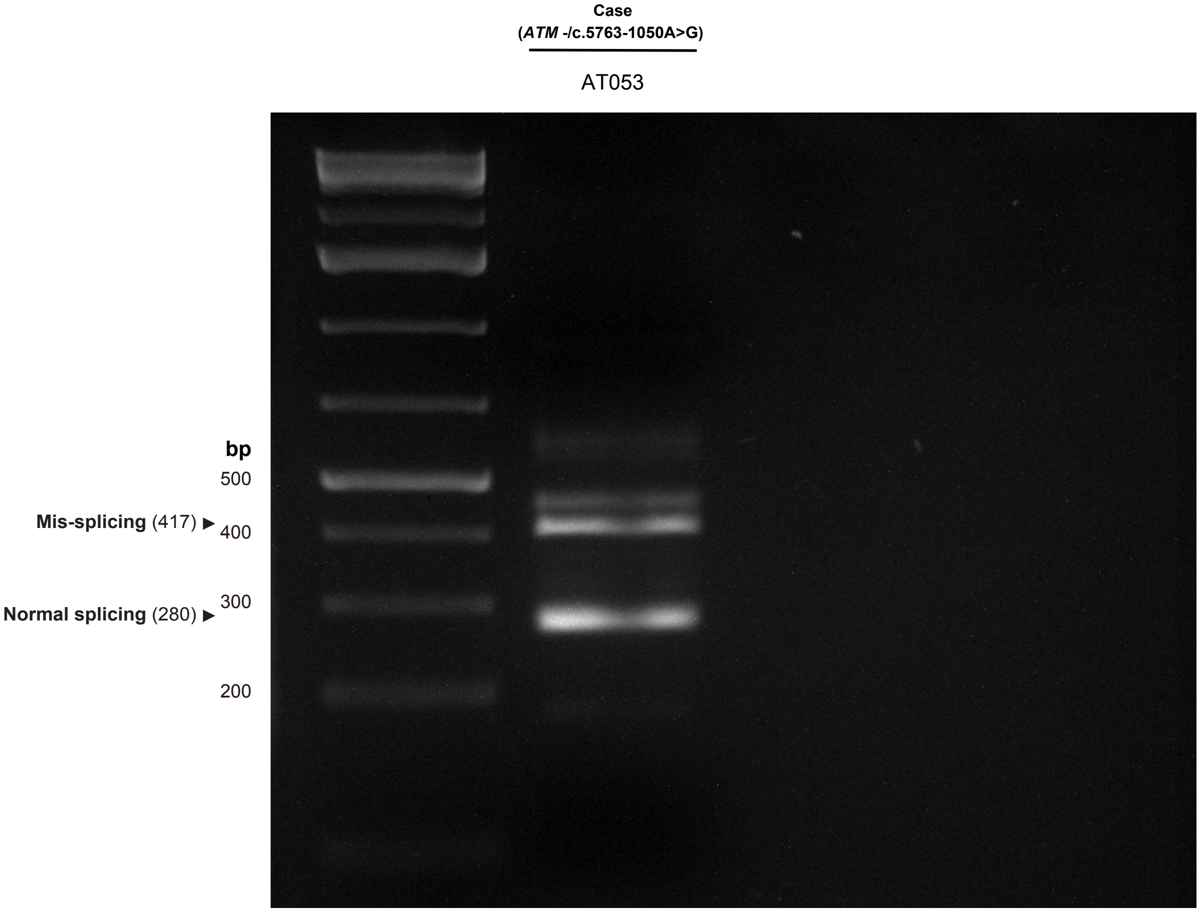

Supplementary Fig. 1 (page 14/18)

Extended Data Fig. 10

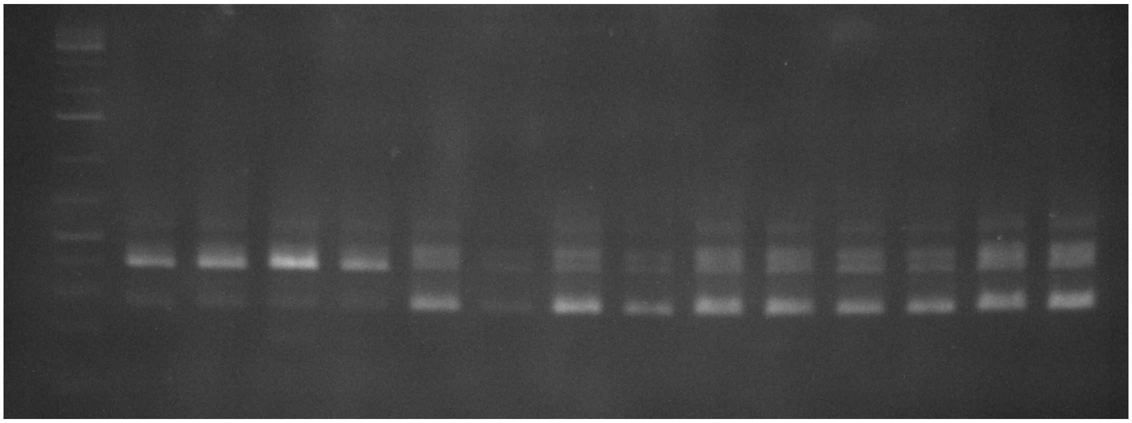

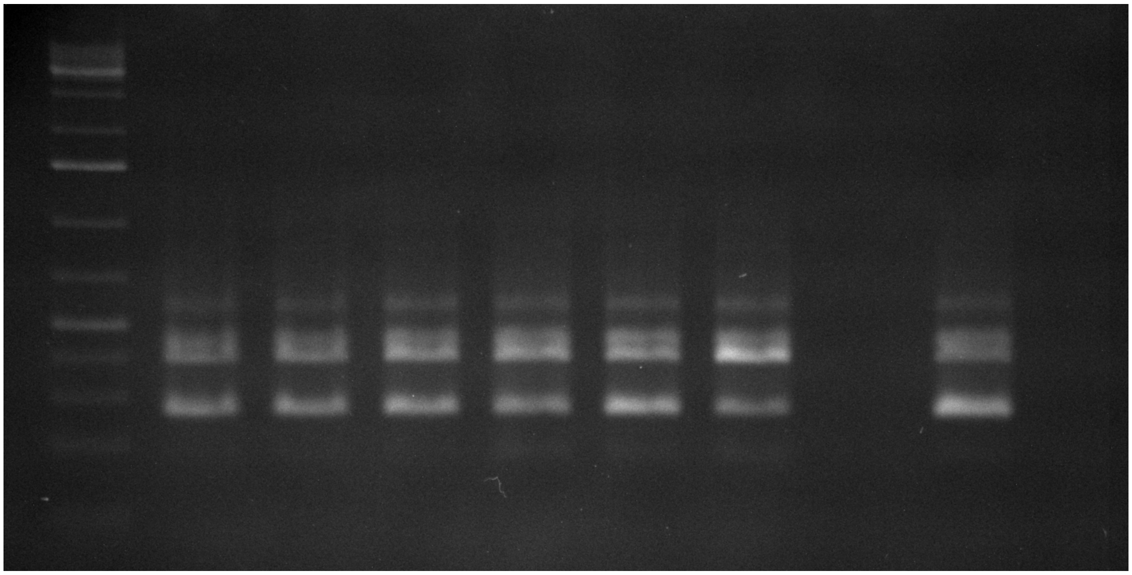

Supplementary Fig. 1 (page 15/18)

Extended Data Fig. 10

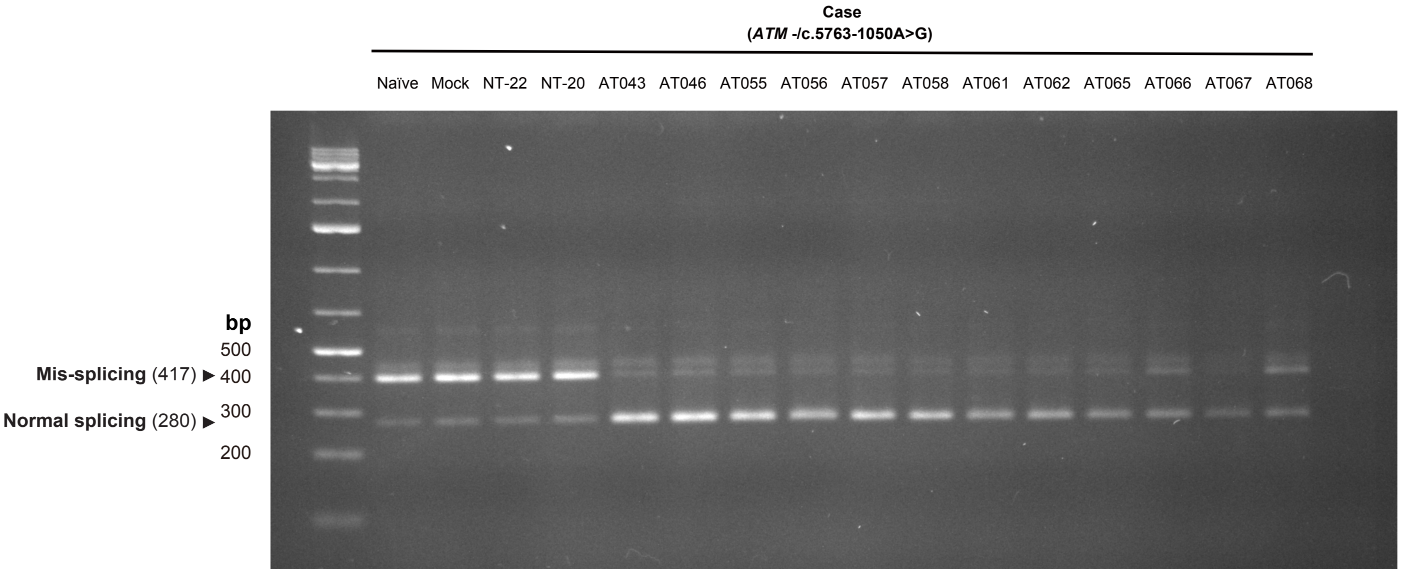

Supplementary Fig. 1 (page 16/18)

Extended Data Fig. 10

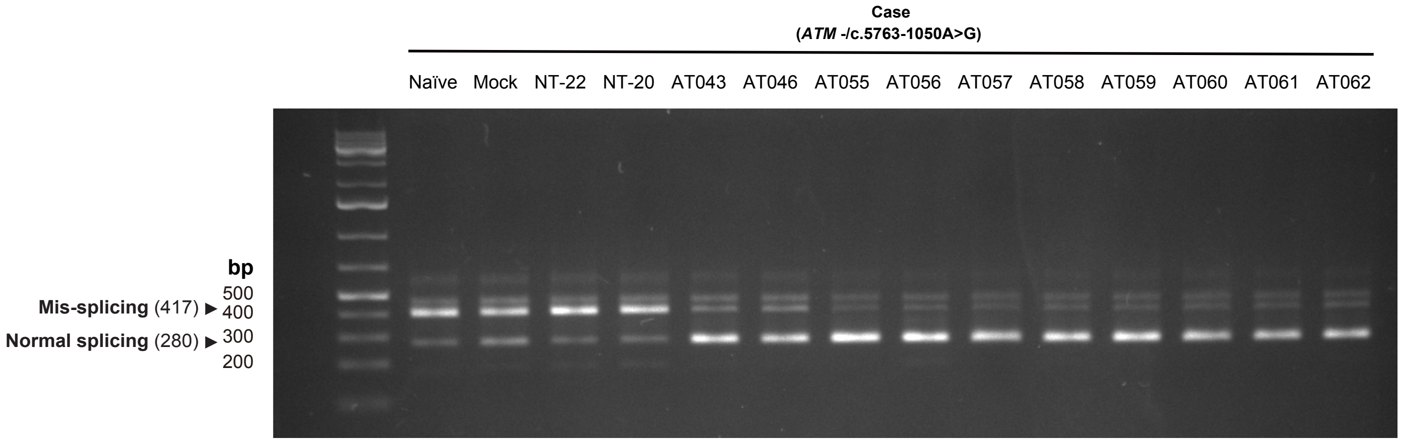

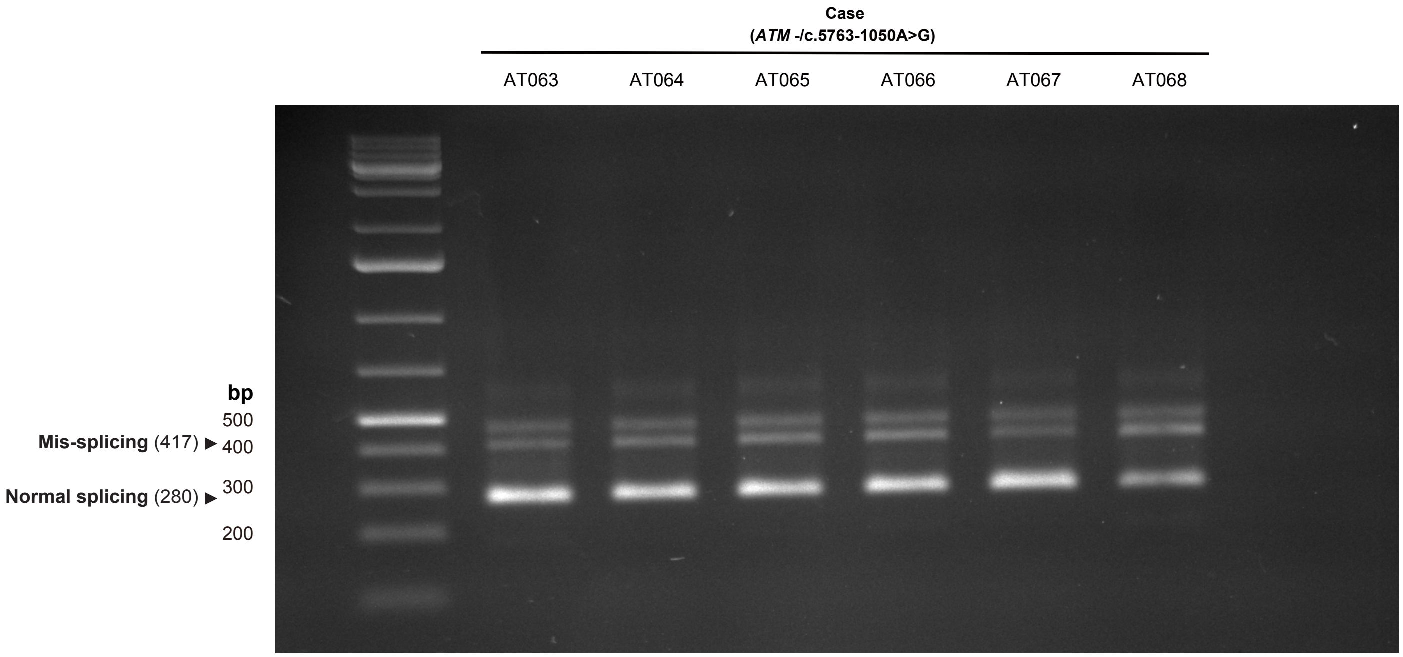

Supplementary Fig. 1 (page 17/18)

Extended Data Fig. 10

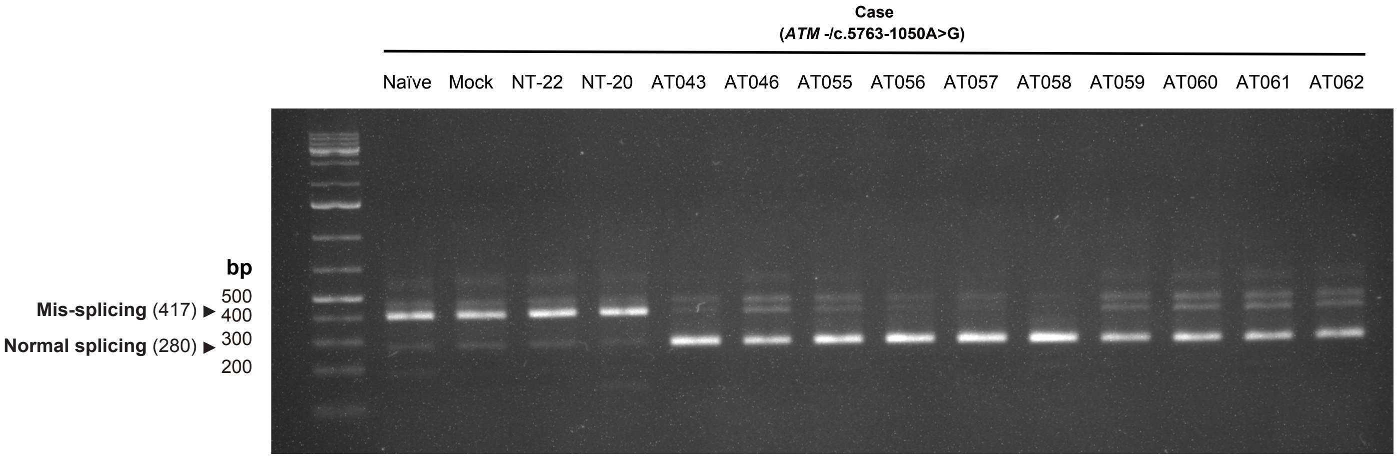

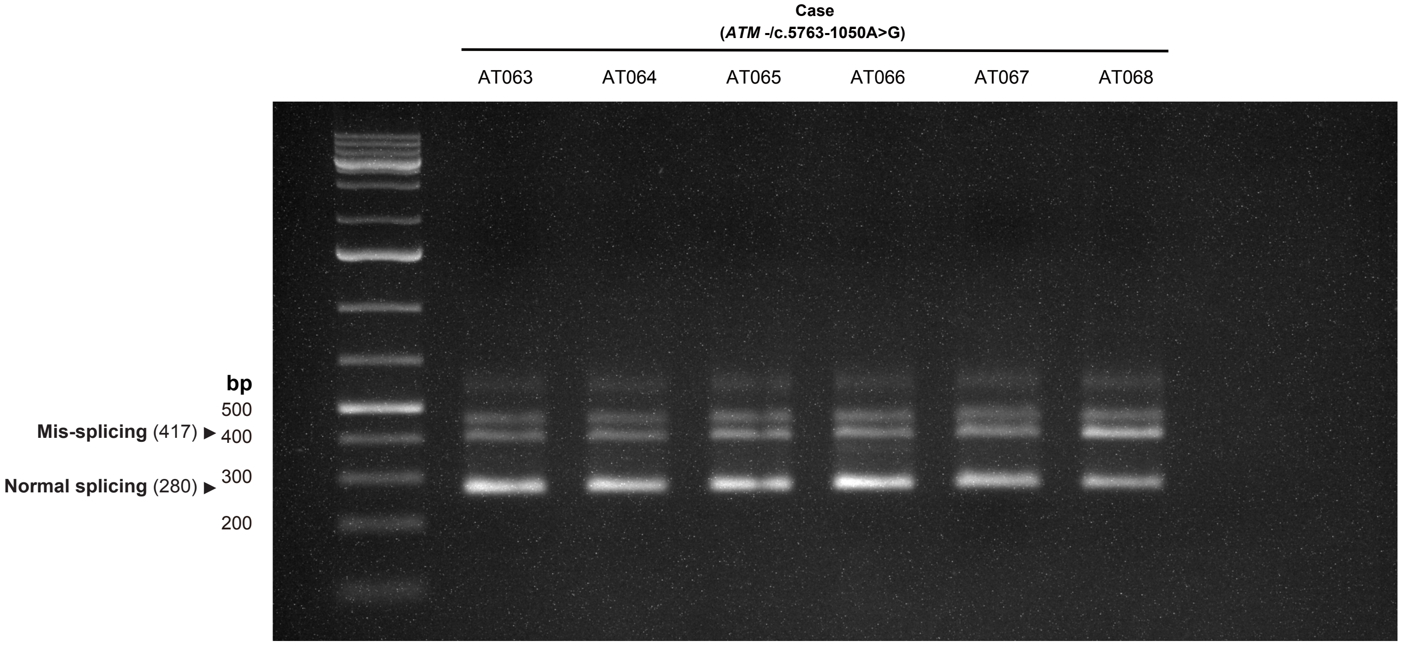

Supplementary Fig. 1 (page 18/18)

Extended Data Figs. 11a,b

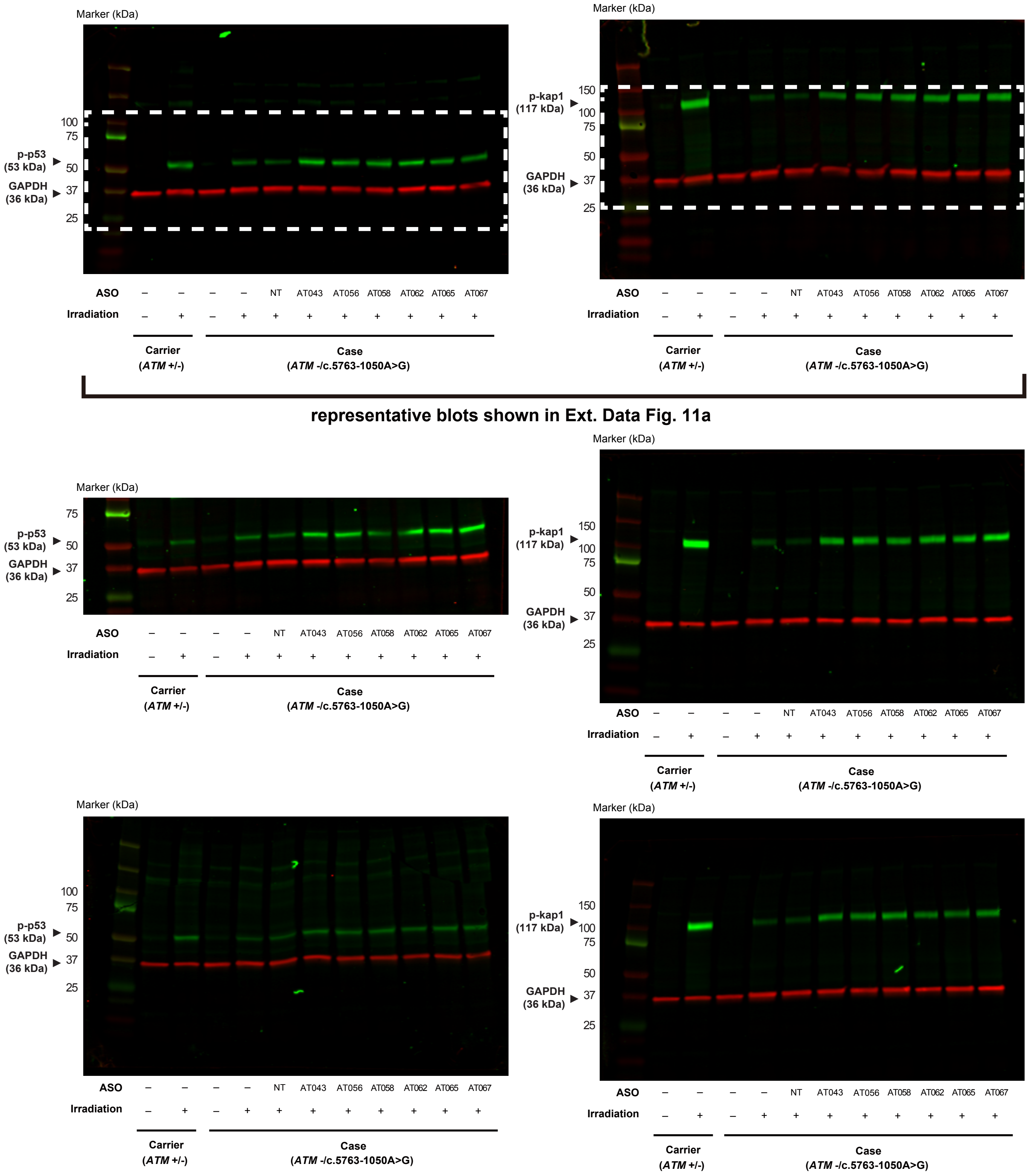

Supplementary Fig 1. Unprocessed gel and blot images
