# Supplementary material for: A framework for individualized splice-switching oligonucleotide therapy
Source: Nature. 2023 Jul 12;619(7971):828–36. doi: 10.1038/s41586-023-06277-0 (PMC10371869; doi:10.1038/s41586-023-06277-0)
Supplement: Supplementary file 2 — Reporting Summary [file 41586_2023_6277_MOESM2_ESM.pdf]

nature portfolio

Corresponding author(s): Timothy W. Yu, Jinkuk Kim

Last updated by author(s): Mar 13, 2023

Reporting Summary

Nature Portfolio wishes to improve the reproducibility of the work that we publish. This form provides structure for consistency and transparency in reporting. For further information on Nature Portfolio policies, see our Editorial Policies and the Editorial Policy Checklist.

Statistics

For all statistical analyses, confirm that the following items are present in the figure legend, table legend, main text, or Methods section.

n/a	Confirmed	
☐	☒	The exact sample size (*n*) for each experimental group/condition, given as a discrete number and unit of measurement
☐	☒	A statement on whether measurements were taken from distinct samples or whether the same sample was measured repeatedly
☐	☒	The statistical test(s) used AND whether they are one- or two-sided<br>*Only common tests should be described solely by name; describe more complex techniques in the Methods section.*
☒	☐	A description of all covariates tested
☐	☒	A description of any assumptions or corrections, such as tests of normality and adjustment for multiple comparisons
☐	☒	A full description of the statistical parameters including central tendency (e.g. means) or other basic estimates (e.g. regression coefficient) AND variation (e.g. standard deviation) or associated estimates of uncertainty (e.g. confidence intervals)
☐	☒	For null hypothesis testing, the test statistic (e.g. *F*, *t*, *r*) with confidence intervals, effect sizes, degrees of freedom and *P* value noted<br>*Give P values as exact values whenever suitable.*
☒	☐	For Bayesian analysis, information on the choice of priors and Markov chain Monte Carlo settings
☒	☐	For hierarchical and complex designs, identification of the appropriate level for tests and full reporting of outcomes
☒	☐	Estimates of effect sizes (e.g. Cohen's *d*, Pearson's *r*), indicating how they were calculated

Our web collection on statistics for biologists contains articles on many of the points above.

Software and code

Policy information about availability of computer code

Data collection	No software was used for data collection.
Data analysis	The manuscript includes descriptions of published software tools used in the study. The full list of the softwares: BWA (version 0.7.17), GATK (version 3.5), VarScan2 (version 2.4.4), Strelka2 (version 2.9.10), Delly (version 0.8.6), Pindel (version 0.2.5b8), MELT (version 2.2.2), and xTea (version 0.1.7), IGV (version 2.8.9), VEP (release 100), MaxEntScan, SpliceAI, LaBranchoR, VCFtools (version 0.1.17), WhatsHap (version 1.0), STAR (version 2.7.5c), Samtools (version 1.10)

For manuscripts utilizing custom algorithms or software that are central to the research but not yet described in published literature, software must be made available to editors and reviewers. We strongly encourage code deposition in a community repository (e.g. GitHub). See the Nature Portfolio guidelines for submitting code & software for further information.

Data

Policy information about availability of data

All manuscripts must include a data availability statement. This statement should provide the following information, where applicable:

- Accession codes, unique identifiers, or web links for publicly available datasets
- A description of any restrictions on data availability
- For clinical datasets or third party data, please ensure that the statement adheres to our policy

The manuscript includes a data availability statement that fully describes the requested information.

March 2021

Field-specific reporting

Please select the one below that is the best fit for your research. If you are not sure, read the appropriate sections before making your selection.

☒ Life sciences ☐ Behavioural & social sciences ☐ Ecological, evolutionary & environmental sciences

For a reference copy of the document with all sections, see nature.com/documents/nr-reporting-summary-flat.pdf

Life sciences study design

All studies must disclose on these points even when the disclosure is negative.

Sample size	235 families were available within the global A-T data registry.
Data exclusions	N/A
Replication	Experimental findings were replicated as described in the main text.
Randomization	N/A
Blinding	N/A

Reporting for specific materials, systems and methods

We require information from authors about some types of materials, experimental systems and methods used in many studies. Here, indicate whether each material, system or method listed is relevant to your study. If you are not sure if a list item applies to your research, read the appropriate section before selecting a response.

Materials & experimental systems

n/a	Involved in the study	
☐	☒	Antibodies
☐	☒	Eukaryotic cell lines
☒	☐	Palaeontology and archaeology
☒	☐	Animals and other organisms
☐	☒	Human research participants
☐	☒	Clinical data
☒	☐	Dual use research of concern

Methods

n/a	Involved in the study	
☒	☐	ChIP-seq
☒	☐	Flow cytometry
☒	☐	MRI-based neuroimaging

Antibodies

Antibodies used	anti-phospho-p53 (Cell Signaling Tech, 9284), anti-phospho-kap1 (Bethyl Lab, A300-767A), anti-GAPDH (Proteintech, 60004-1-Ig)
Validation	- anti-phospho-p53: tested reactivity (Human, Mouse, Rat, Monkey), tested application (WB, IP, ChIP), https://www.cellsignal.com/products/primary-antibodies/phospho-p53-ser15-antibody/9284<br>- anti-phospho-kap1: tested reactivity (Human, Mouse), tested application (ICC, ICC-IF, IHC, IP, WB), https://www.fortislife.com/products/primary-antibodies/rabbit-anti-phospho-kap-1-s824-antibody/BETHYL-A300-767<br>- anti-GAPDH: tested reactivity (Human, Mouse, Rat, Yeast, Plant, Zebrafish), tested application (WB, IP, IHC, IF, FC, CoIP, ChIP, ELISA), https://www.ptglab.com/Products/GAPDH-Antibody-60004-1-Ig.htm

Eukaryotic cell lines

Policy information about cell lines

Cell line source(s)	Skin fibroblasts from two patients and their family members (established from skin biopsy samples)
Authentication	The cell lines were subjected to RNA-seq and the patient-specific mutations were confirmed.
Mycoplasma contamination	The cell lines were not tested for mycoplasma contamination.
Commonly misidentified lines (See ICLAC register)	None

Human research participants

Policy information about studies involving human research participants

Population characteristics	See Extended Data Table 1 and Supplementary Table 1.
Recruitment	The participants were recruited by the A-T Children's Project, a patient advocacy foundation for A-T, and all A-T patients who agreed to provide a sample were enrolled. Since the recruitment process was carried out independently of the current study design, it is unlikely that any self-selection bias could have influenced the results.
Ethics oversight	The IRB of Boston Children's Hospital has approved the study protocol.

Note that full information on the approval of the study protocol must also be provided in the manuscript.

Clinical data

Policy information about clinical studies

All manuscripts should comply with the ICMJE guidelines for publication of clinical research and a completed CONSORT checklist must be included with all submissions.

Clinical trial registration	Not applicable (the study was approved by the FDA as a single patient expanded access IND)
Study protocol	Since the trial is still going on, the results of the clinical trial will be published in a separate manuscript, accompanied by the study protocol.
Data collection	The n-of-1 clinical trial has been conducted at Boston Children's Hospital since January 2020 till now.
Outcomes	Since the trial is going on, the outcomes will be reported in a follow-up manuscript.
