# Supplementary material for: A framework for individualized splice-switching oligonucleotide therapy
Source: Nature. 2023 Jul 12;619(7971):828–36. doi: 10.1038/s41586-023-06277-0 (PMC10371869; doi:10.1038/s41586-023-06277-0)
Supplement: Supplementary file 3 — This file contains Supplementary Notes, Supplementary Figures 2-12, Supplementary Tables 5, 6 and 11, and Supplementary References. [file 41586_2023_6277_MOESM3_ESM.pdf]

Supplementary Information Guide

for

A Framework for Individualized Splice-Switching Oligonucleotide Therapy

by Kim, Woo, de Gusmao, Zhao *et al.*

<u>TABLE OF CONTENTS</u>

SUPPLEMENTARY NOTES 1–8 2

SUPPLEMENTARY FIGURES 1–12 14

SUPPLEMENTARY TABLES 1–16 27

REFERENCES 35

SUPPLEMENTARY NOTES 1–8

Supplementary Note 1. VUSs that were considered as disease candidate variants.

Copy number variants: Two copy number variants (CNVs), which are duplications involving *ATM,* were classified as uncertain significance according to ACMG guidelines for CNVs (**Extended Data Fig. 2, Supplementary Table 3**).

- Exon 17–61 duplication: It duplicates ~70% of the coding region of *ATM*. Although it is in-frame and not expected to cause nonsense-mediated decay, the duplicated region is so large that it seems reasonable to expect that it severely disrupts the protein function. The patient DDP_ATCP_179 is heterozygous for this CNV and an *ATM* stop-gain variant, c.1110C>G (p.Tyr370Ter). The other variants found in this patient were determined unlikely disease-causing variants.
- Exon 53–61 duplication: It duplicates ~12% of the coding region of *ATM*. Although it is in-frame and not expected to cause nonsense-mediated decay, the duplicated region is sufficiently large that it seems reasonable to expect that it can disrupt the protein function. The patient DDP_ATCP_400 is heterozygous for this CNV and an *ATM* intronic variant, c.5763-1050A>G (a known pathogenic variant causing pseudoexon inclusion)[1]. The other variants found in this patient were determined unlikely disease-causing variants.

Large inversion: Unlike for CNVs, there are no specialized ACMG guidelines for complex structural variant events such as inversions – therefore, we used ACMG guidelines for single nucleotide variants/short indels. A heterozygous inversion event spanning a >463 kb region from *SLC35F2* intron 7 to *ATM* intron 16 was found in the patient DDP_ATCP_289 (**Supplementary Fig. 3, Supplementary Table 3**). Although it completely dislocates the first 16 exons and is expected to destroy the gene function, it is classified as uncertain significance due to the lack of applicable criteria in ACMG guidelines. Only PM2 criterion could be applied based on the fact that this inversion has not been reported in public SV databases. The patient DDP_ATCP_289 is heterozygous for this SV and an *ATM* frameshift variant, c.3245_3247delATCinsTGAT (p.His1082LeufsTer14). The other variants in *ATM* found in this patient were determined unlikely disease-causing variants.

Pseudogene insertion: A homozygous pseudogene (*DUSP16*) insertion variant was found in the patient DDP_ATCP_394 (**Supplementary Table 3**). The variant was classified as uncertain significance based on the computational prediction that it induces mis-splicing and pseudoexon inclusion (PP3) and the fact that it has not been reported in controls (PM2). The other variants in *ATM* found in this patient were determined unlikely disease-causing variants.

Tandem duplication: A heterozygous 382 bp tandem duplication variant was found in the patient DDP_ATCP_395 (**Fig. 3, Supplementary Fig. 4, Supplementary Table 3**). This variant is predicted by SpliceAI to activate cryptic splice acceptor and donor sites and cause the inclusion of a pseudoexon that contains a premature termination codon (**Fig. 3**). Based on this prediction (PP3) and the fact that it has not been reported in control databases (PM2), it is classified as uncertain significance. The patient DDP_ATCP_395 also harbors a heterozygous *ATM* frameshift variant, c.2730_2731insAG (p.Ala911ArgfsTer19). The other variants in *ATM* found in this patient were determined unlikely disease-causing variants.

Transposon (Alu) insertion: 2 Alu insertion variants in *ATM* (3 events, one *n* = 1 and one *n* = 2; **Supplementary Table 3**) were classified as uncertain significance based on the SpliceAI/MaxEntScan prediction that they cause exon skipping and subsequent frameshift (PP3) and the fact that they are absent in the gnomAD SVs v.2.1 database (PM2). The patients who were heterozygous for one of these Alu insertion variants also harbor a heterozygous disease candidate variant in *ATM* (**Supplementary Table 1**). The other variants in *ATM* found in these patients were determined unlikely disease-causing variants.

Synonymous variant: A synonymous variant, c.3489C>T (p.Ser1163=), was classified as uncertain significance based on the SpliceAI/MaxEntScan prediction that the variant causes mis-splicing and subsequent exon truncation (PP3) and absent population allele frequency in the gnomAD v.3.1 and the TOPMed freeze 8 databases (PM2; **Fig. 3**, **Supplementary Table 9**). The patient who was heterozygous for the synonymous variant also harbors a heterozygous *ATM* stop-gain variant, c.4432C>T (p.Gln1478Ter) (**Supplementary Table 1**). The other variants in *ATM* found in this patient were determined unlikely disease-causing variants.

Missense variants: 14 missense variants were classified as uncertain significance based on the SpliceAI/MaxEntScan or REVEL prediction (PP3) and absent or low population allele frequency (PM2).

- A missense variant, c.4801A>G (p.Ser1601Gly), was classified as uncertain significance based on the SpliceAI/MaxEntScan prediction that the variant causes mis-splicing and subsequent exon truncation (PP3) and an absent or extremely low prevalence in the gnomAD v.3.1 and the TOPMed freeze 8 databases (PM2; **Fig. 3**, **Supplementary Table 9**). The patient who was heterozygous for the missense variant also harbors a heterozygous *ATM* frameshift variant, c.1402_1403del (p.Lys468GlufsTer18) (**Supplementary Table 1**). The other variants in *ATM* found in this patient were determined unlikely disease-causing variants.
- 13 missense variants in *ATM* (**Supplementary Table 7**) were classified as uncertain significance based on the REVEL prediction that they are detrimental (PP3) and the fact that they are absent or have an extremely low prevalence in the gnomAD v.3.1 and the TOPMed freeze 8 databases (PM2). The patients who were heterozygous for one of these missense variants also harbor another heterozygous disease candidate variant in *ATM* (**Supplementary Table 1**). The other variants in *ATM* found in these patients were determined unlikely disease-causing variants.

Supplementary Note 2. VUSs that were not considered as disease candidate variants.
In two of the 235 A-T patients in the ATCP cohort (DDP_ATCP_299, DDP_ATCP_368), we found one disease candidate variant in *ATM* and one variant of uncertain significance (VUS) in *ATM*. We did not consider the VUSs as disease candidate variants for now, but has the potential to be ones with future validation.

A missense variant, c.7328G>A, in DDP_ATCP_299: This individual had two coding region variants in *ATM*. The first was an in-frame deletion variant, c.7638_7646del (p.Arg2547_Ser2549del), which was experimentally validated to confer complete loss-of-function[2]. The second was a missense variant that is a VUS, c.7328G>A (p.Arg2443Gln). The allele frequency of the VUS (gnomAD v.3.1, 0.0000069771; TOPMed freeze 8, 0.000003778) was compatible with the prevalence of A-T (less frequent than the most prevalent, previously

known pathogenic variant, c.5932G>T (p.Glu1978Ter), found in the ATCP cohort; gnomAD v.3.1, 0.0000349045; TOPMed freeze 8, 0.000007556). Although in silico prediction by REVEL did not support pathogenicity of the missense variant (REVEL, "Benign" (0.466)), the variant is located in the FAT domain, which interacts with ATM's kinase domain to stabilize the C-terminus region of ATM. No experimental data in the literature were found for this variant. No other potentially disease-causing SV was found involving the *ATM* region either by computational tools or by manual inspection of sequencing reads on IGV. The only other *ATM* variants found in this individual were a 3'UTR variant and intronic variants that we deemed unlikely to be contributory, as MaxEntScan and SpliceAI predicted they lack mis-splicing potential and the ENCODE browser[3] did not indicate overlap with any potential regulatory elements.

A potential enhancer-disrupting variant, c.496+670T>G, in DDP_ATCP_368: This patient harbored a pathogenic missense variant, c.8494C>T (p.Arg2832Cys). REVEL strongly predicted pathogenicity: "Pathogenic" (0.830). This variant was experimentally validated to confer reduced ATM protein level and reduced kinase activity.[4] All the other *ATM* region variants found in this patient were intronic variants. MaxEntScan and SpliceAI predicted they lacked mis-splicing potential, but one, c.496+670T>G was positioned in the distal enhancer-like elements cataloged by ENCODE (**Extended Data Fig. 1**). This variant had an allele frequency (gnomAD v.3.1, 0.0; TOPMed freeze 8, 0.0) compatible with the prevalence of A-T (less frequent than the most prevalent, previously known pathogenic variant, c.5932G>T (p.Glu1978Ter), found in the ATCP cohort; gnomAD v.3.1, 0.0000349045; TOPMed freeze 8, 0.000007556). No other potentially disease-causing SV was found involving the *ATM* region either by computational tools or by manual inspection of sequencing reads on IGV.

Supplementary Note 3. Correction of clinical genetic testing results.

Clinical testing results were available for 55 (23.4%) of the 235 individuals in the cohort. In three cases (DDP_ATCP_78, DDP_ATCP_160, DDP_ATCP_248), our analyses led to a disqualification of previously reported variants that had been originally interpreted as pathogenic; instead, in each case we nominated a different variant as a disease candidate variant.

DDP_ATCP_78: Clinical genetic testing reported two variants as potentially disease-causing: a frameshift variant c.8395_8404del (p.Phe2799LysfsTer4) and a missense variant c.2932T>C (p.Ser978Pro). Whereas the frameshift variant is pathogenic by the ACMG classification, the missense variant, although it is predicted pathogenic (REVEL, "Pathogenic" (0.874)), is unlikely disease-causing. The allele frequencies of the variant in general populations (gnomAD v.3.1, 0.000404932; TOPMed freeze 8, 0.000370244) were more than 10 times higher than that of c.5932G>T (p.Glu1978Ter; gnomAD v.3.1, 0.0000349045; TOPMed freeze 8, 0.000007556), the known pathogenic variant with the highest prevalence in the ATCP cohort, despite the fact that the allelic prevalence of this variant in the cohort (5/470) is more than 3 times lower than that of c.5932G>T (16/470). Moreover, in other individuals in this cohort, this variant was found in patients with two other pathogenic hits, which suggests that it is unlikely a disease-causing variant. For instance, (1) c.2932T>C was found in the homozygous state in an individual (DDP_ATCP_122) who also had a homozygous frameshift variant c.8395_8404del (p.Phe2799LysfsTer4), (2) it was found in the heterozygous state in two siblings (DDP_ATCP_28, DDP_ATCP_29) who also had two heterozygous variants, a frameshift variant c.217_218del (p.Glu73MetfsTer26) and a stop-gain variant c.4735C>T (p.Gln1579Ter), and (3) it was found in the heterozygous state in an individual (DDP_ATCP_78) who also had two heterozygous variants, a frameshift variant c.8395_8404del (p.Phe2799LysfsTer4) and a synonymous variant c.2250G>A (p.Lys750=). Although c.2250G>A (p.Lys750=) is a synonymous variant, since it is located in the last base of *ATM* exon 14, it damages the canonical splice donor site of the exon (MaxEntScan donor site score changed from 9.45 to 3.63; SpliceAI donor loss and acceptor loss score, 0.94 and 0.77, respectively). The exon 14 skipping event caused by the variant was experimentally verified by this study (**Extended Data Fig. 5**) as well as a previous study[5]. It has an allele frequency of 0.0000279 in gnomAD v.3.1, and was found in five individuals in the cohort; for all of these patients, the clinical diagnosis of A-T cannot be explained without considering this variant as disease-causing.

DDP_ATCP_160: Clinical genetic testing reported a "homozygous Norwegian - 3245ATC>TGAT variant." However, our WGS analysis found that the frameshift variant is heterozygous. Instead, our analysis found another heterozygous variant that can be the second disease-causing hit for this patient: c.7914G>T (p.Trp2638Cys). This missense variant is

predicted to have damaging coding impact by REVEL ("Pathogenic" (0.604)), and its allele frequency is compatible with the prevalence of A-T: gnomAD v.3.1, 0.0000069811; TOPMed freeze 8, 0.00000378. Lacking experimental validation yet, this variant is classified as uncertain significance by ACMG guidelines, but we considered it as a disease candidate variant, because there is no other variant in this individual that completes the genetic diagnosis for this patient.

DDP_ATCP_248: Clinical genetic testing reported two heterozygous variants as potentially disease-causing: a stop-gain variant, c.5932G>T (p.Glu1978Ter) and an intronic deletion variant, c.3402+32_3402+34del. Whereas the stop-gain variant is pathogenic by the ACMG classification, the intronic variant is unlikely to be disease-causing because (1) it is unlikely to damage a canonical splice site because it is relatively far (32 bp away) from the closest donor site, (2) the site of the variant is not conserved in mammals (*e.g.*, mice and rats), (3) the allele frequency of the variant (gnomAD v.3.1, 0.00155622; TOPMed freeze 8, 0.00145453) is too high to be a causal variant for A-T. Our WGS analysis found an alternative variant that is classified as pathogenic by ACMG guidelines: c.8147T>C (p.Val2716Ala) (**Supplementary Table 1**).

Supplementary Note 4. An A-T patient with c.7865C>T.

This is a currently 6-year-old girl with an uncommonly early diagnosis of A-T. Her prenatal medical history was unremarkable, but she came to medical attention at birth after newborn screening revealed a concern for possible severe combined immunodeficiency (SCID). This was on the basis of an abnormally low T Cell Receptor Excision Circle count. However, she exhibited no clinical signs or symptoms of SCID and appeared otherwise healthy. Her physical and neurologic examination were unremarkable. Her paternal grandmother had ovarian cancer, but otherwise there was no known family history of neurologic, immunologic, or neoplastic conditions in first or second-degree relatives. Her medical workup eventually included whole exome sequencing which led to a diagnosis of A-T at 7 months of age (making her one of the youngest patients ever diagnosed with A-T). Only a handful of patients with A-T have been diagnosed in this manner (due to the relatively recent rollout of newborn screening for SCID in some states in approximately 2011); the first two infants to arrive at an A-T diagnosis via this route were reported in 2013[6].

This individual has two variants in *ATM*: c.8585-13_8598del, inherited from her father, and c.7865C>T (p.Ala2622Val), inherited from her mother (*i.e.*, both of her copies of *ATM* are mutant). These variants are predicted to be null variants (*i.e.*, expected to produce no active ATM kinase protein) and are strongly associated with a "classical" (severe) A-T phenotype and poor prognosis[7]. In more detail:

- c.8585-13_8598del, the paternal allele, is a deletion at the boundary of intron 58 and exon 59, and is strongly predicted to be a molecular null (*i.e.*, produce no functional protein): (1) it directly removes coding sequence for 5 highly conserved amino acids from the ATM PI3 kinase domain, and (2) it removes the exon 59 splice acceptor, resulting in skipping of exon 59 (87 bp, 29 amino acids) which includes two residues that are experimentally proven to be critical for the ATM kinase activity[8].
- c.7865C>T (p.Ala2622Val), the maternal allele, creates a new (*i.e.*, extra) splice donor site within exon 55. Despite the continued presence of the normal splice-donor site, the new site is used exclusively, resulting in the deletion of 64 nucleotides at the end of the exon. It has been seen in the homozygous state in patients with classical A-T, and fibroblasts with this variant harbor no residual protein or enzymatic activity[9] (Malcolm Taylor, personal communication). Despite this variant being predicted to also have a benign coding impact (p.Ala2622Val; predicted to be benign by REVEL), its pathogenic impact is predominantly mediated by mis-splicing[10].

A few months after her diagnosis, the patient was referred to Boston Children's Hospital by A-T Children's Project (ATCP), an advocacy organization for patients and families impacted by A-T.

Supplementary Note 5. Preclinical development of ASOs targeting c.5763-1050A>G.

In parallel with the preclinical development of AT008, serial screening and validation experiments were performed for ASOs that target c.5763-1050A>G. A fibroblast cell line was established from an A-T patient with c.5763-1050A>G in the ATCP cohort, DDP_ATCP_42. In light of the potential for an individualized trial, this patient was selected based on her relatively young age at the cohort enrollment (age at enrollment, 12) and the delayed onset of disease symptoms (age at A-T diagnosis, 11.67; age at onset of first symptom, 9; age at onset of first neurologic symptom, 10). Using the patient-derived fibroblasts, mis-splicing consequence of

c.5763-1050A>G, 137 nt pseudoexon inclusion between exon 38 and exon 39, was confirmed by allele-specific PCR and RNA-seq (**Fig. 3, Extended Data Fig. 9**). The allele-specific PCR result demonstrated the leakiness of the mis-splicing event.

For the initial round of screening, thirteen ASOs (AT042-AT054) were designed to block the pseudoexon usage induced by the c.5763-1050A>G variant, targeting either a cryptic splice acceptor site (AT042), a predicted splice regulatory element in the pseudoexon (AT043-AT045), or a strengthened splice donor site (AT046-AT054) (**Extended Data Fig. 10, Supplementary Table 13**). Within the initial round of thirteen ASOs, AT043 and AT046 exhibited the highest efficacy. Of the two ASOs, AT043 was chosen for optimization, as AT046 has a CpG dinucleotide, a potential immunogenic motif. Fourteen additional ASOs (AT055-AT068) were designed by altering the length or offset from AT043. Upon evaluating the twenty ASOs in conjuction with AT043 and AT046, four fine-tuned ASOs (AT055-AT058) showed slightly enhanced efficacy, with AT056 emerging as the most effective.

To identify functional rescue of ATM kinase activity, six ASOs were selected for immunoblotting analysis based on their efficacy and length (picking one ASO from each of the 17-, 18-, 19-, 20-, 21-, and 22-mers; AT067, AT065, AT062, AT043, AT058, and AT056 respectively). In the immunoblotting analysis for phospho-P53 (p-P53) and phospho-KAP1 (p-KAP1), all six tested ASOs showed increased levels of p-P53 and p-KAP1, although statistical significance was observed only for p-KAP1 levels compared to non-targeting ASO-transfected cells (**Extended Data Fig. 11**). Additionally, three ASOs (AT043, AT056, AT057) were selected for RNA-seq validation. Pseudoexon inclusion levels were decreased from 18.4-20.7% (untreated patient fibroblasts) to 7.7-11.4% by the ASO treatment. Among the three ASOs, AT056 showed the highest efficacy for mitigating the pseudoexon inclusion.

Based on the results of screening and validation experiments, AT056 was nominated as the lead ASO for c.5763-1050A>G. In silico off-target analysis and in vitro toxicity assay were performed for AT056. Like AT008 and AT026, the off-target profiles of AT056 were clean (**Supplementary Fig. 11**). In the in vitro toxicity assay, AT056 showed comparable tolerability with non-targeting control ASO (**Supplementary Fig. 9**).

Supplementary Note 6. Preliminary clinical observations in an A-T patient with c.7865C>T who has been treated with AT008/atipeksen.

A single A-T individual was enrolled in a clinical investigational protocol to receive the mutation-specific antisense oligonucleotide, atipeksen. This individual's newborn medical history and A-T diagnosis are described in **Supplementary Note 4**. Over the first two years of life, she met appropriate social and language developmental milestones, but exhibited motor delay (independent walking at age 24 months). Mild truncal and gait ataxia were also evident – consistent with classic A-T in which ataxic gait is the first presenting symptom, with a median onset of 1.5 years[11] (and consistent with the fact that both of her *ATM* variants were predicted to produce no active ATM kinase protein).

Intrathecal administration of AT008/atipeksen began at age 2 years and 9 months. At that time she was at the 5th percentile for weight-for-age. Her body mass index (BMI) was 16.4 (5th percentile). Her neurological examination demonstrated gait imbalance, with swaying of her trunk in sitting and standing stances. Speech was mildly dysarthric and there was intermittent drooling. Sight choreiform movements were noted in her extremities. She would run better than walking. The remainder of the exam was unremarkable. Nerve conduction studies were normal.

To date she has been receiving the investigational drug atipeksen for over three years. Her most recent clinical examination was at the age of 5 years and 8 months. Her BMI was 19.5 (70th percentile). Conjunctival telangiectasias were evident. Her oculomotor function demonstrated slight saccadic breakdown but no evidence of oculomotor apraxia. Dysarthria was still present, but drooling had improved. Minimal choreiform movements were detectable in her distal upper extremities. There was no appendicular dysmetria on a peg board test; she could button clothes and appropriately use a pair of scissors. She could stand still for 30 seconds without imbalance, although truncal swaying was intermittently present. With encouragement, she could walk tandem for a few steps. On walking longer distances some staggering was noted but she could turn without loss of balance; she did not require bracing or adaptive equipment. Her overall trajectory to date has been notable for developmental gains in social/language skills, cognition, and motor function, and her current clinical exam appears to be on the mild end of young children with A-T[12]. This trial is still ongoing.

Supplementary Note 7. Prevalence of ASO-amenable variants in *ABCA4* deficiency cohorts.
To assess the prevalence of ASO-amenable variants in genetic conditions other than A-T, we analyzed published datasets for an autosomal recessive retinal disease caused by biallelic loss-of-

function of *ABCA4* (Stargardt disease or cone-rod dystrophy; collectively referred to as "*ABCA4* deficiency" herein). We chose *ABCA4* deficiency because it was the only genetic disease we could find for which comprehensive genomic sequencing had been performed in a large and well-characterized cohort of individuals with the same clinical diagnosis. We demonstrate that the prevalence of ASO-amenable variants in this disease is in line with our estimates for A-T.

Two studies reported disease variants in 70 and 506 patients[13,14]; we will refer to these as the French and Spanish cohorts, respectively. We performed an extensive literature search to find experimental evidence supporting ASO-amenability of the disease variants, and concluded that 16-21% and 4-13% of the French and Spanish *ABCA4* deficiency patients, respectively, harbor an ASO-amenable variant, which is in line with the estimate in A-T.

(1) "Prevalence of *ABCA4* Deep-Intronic Variants and Related Phenotype in An Unsolved One-Hit Cohort with Stargardt Disease," Nassisi *et al.* (2019) described 70 French patients clinically diagnosed with *ABCA4* deficiency, but with only one pathogenic variant previously identified in each patient (*i.e.*, half-diagnosed). The pathogenic variants were mostly in the exonic regions of *ABCA4*, as they were mostly identified by exon-centric approaches. Since these exonic variants are less likely to be amenable to ASO therapy, only one variant was classified as an ASO-amenable variant (in the possibly category; **Supplementary Table 15**).

Assuming that the missing second variant is located in an intronic region, the authors performed targeted Sanger sequencing of 24 candidate intronic regions encompassing previously reported intronic *ABCA4* variants (spanning 3.5% of the intronic region of *ABCA4*). In 15 of the 70 patients, an intronic disease-causing variant was discovered, and all of the discovered variants were ASO-amenable variants (11 in the probably category and 4 in the possibly category). In other words, 21% (15/70) of the patients have at least one (either probably or possibly) ASO-amenable variant (one of the 15 patients had both a probably and a possibly ASO-amenable variant, one in each allele).

Notably, the authors' strategy had a low diagnostic yield, identifying biallelic hits in only 21% of their cohort (15/70 patients). Because the other 55 patients still remained half-diagnosed, the ASO-amenability estimate of 16-21% (16% if counting only probably ASO-amenable variants) could go up if the missing second variants are identified in other intronic regions. On the other hand, generalizability of this estimate requires caution since this study

population could have been subject to selection bias. Given that it included patients with a higher likelihood of finding intronic variants, it could be overestimating ASO-amenability and may not be reflective of the total population of individuals with *ABCA4*-disease. With these two considerations in balance, we considered the estimate of 16-21% is considered roughly in line with our estimate of 9-15% obtained from the A-T cohort.

(2) "Genotype–Phenotype Correlations in a Spanish Cohort of 506 Families With Biallelic *ABCA4* Pathogenic Variants," Del Pozo-Valero *et al.* (2019) described 506 Spanish patients with confirmed genetic diagnosis of *ABCA4* deficiency. Among the 506 patients, 21 (21/506, ~4%) and 47 (47/506, ~9%) were found to have a probably and a possibly ASO-amenable variant, respectively. This result suggests an ASO-amenability of 4-13% for this cohort (**Supplementary Table 16**). This ASO-amenability may be an underestimate of the real fraction. In contrast with the French cohort, in this study, only patients with biallelic pathogenic variants identified by exon-centric approaches were included. Since the majority of variants in this population were exonic, the ASO-amenability rate is likely lower since exonic variants are less likely to be amenable to ASO therapy.

Acknowledging the inherent biases of these cohorts, we consider that the prevalence of ASO-amenable variants in the two studies describing cohorts with *ABCA4* deficiency (16–21% and 4-13%) is generally commensurate with the estimate we obtained from the A-T cohort (9–15%). It supports the notion that a substantial fraction of patients with autosomal recessive diseases might be addressable by splice-switching ASO therapy.

Supplementary Note 8. Choosing intrathecal injection over intracerebroventricular injection as the route of drug administration for the patient.

In principle it is possible that intracerebroventricular (ICV) injection may achieve better cerebellar distribution than intrathecal (IT) injection because of the relative proximity of the injection site to the cerebellum. Despite this theoretical advantage, we deferred from using this modality due to safety concerns. Repeated ICV injections in humans would require neurosurgical implantation of an indwelling ventricular catheter connected to a subcutaneous drug port in the scalp, such as the Ommaya reservoir. This carries inherent risks including subdural hematoma,

misplacement of the catheter and infections such as ventriculitis. The latter could be particularly ominous in a patient harboring a disease that in itself affects the immune system. Furthermore, while there is experience with intraventricular use of chemotherapeutic agents and antibiotics, safety data regarding ICV ASO injections is sparse. In one study[15], use of intraventricular nusinersen was associated with complications including infection and catheter separation. We also note that in animal studies, mice treated with high dose ICV ASO injections may exhibit sedation and seizures.

Since cerebellar exposure to intrathecally administered ASOs is 2–5X lower than spinal cord, we employed doses of atipeksen (**Extended Data Fig. 12**) that were ~5 times higher than the current FDA-approved standard nusinersen dose of 12 mg. We also employed maintenance dosing intervals as short as 2 months (compared to the nusinersen maintenance dosing interval of 4 months). In a PK simulation (assuming the half-life of 150 days), this regimen provides up to ~9 times higher ASO steady-state level in CNS tissues compared to nusinersen dosing regimen (**Supplementary Fig. 12**), sufficient to compensate for the biodistribution gradients discussed above. In any case, with further de-risking of ICV injections – or under different clinical risk-benefit scenarios – the intraventricular route may play an important role in future ASO trials.

SUPPLEMENTARY FIGURES 1–12

Supplementary Fig. 1. Unprocessed gel and blot images.

Supplementary Fig. 2.

A complex structural variant (SV), involving deletion and inversion

a Schematic and IGV screenshot of a complex SV in DDP_ATCP_529

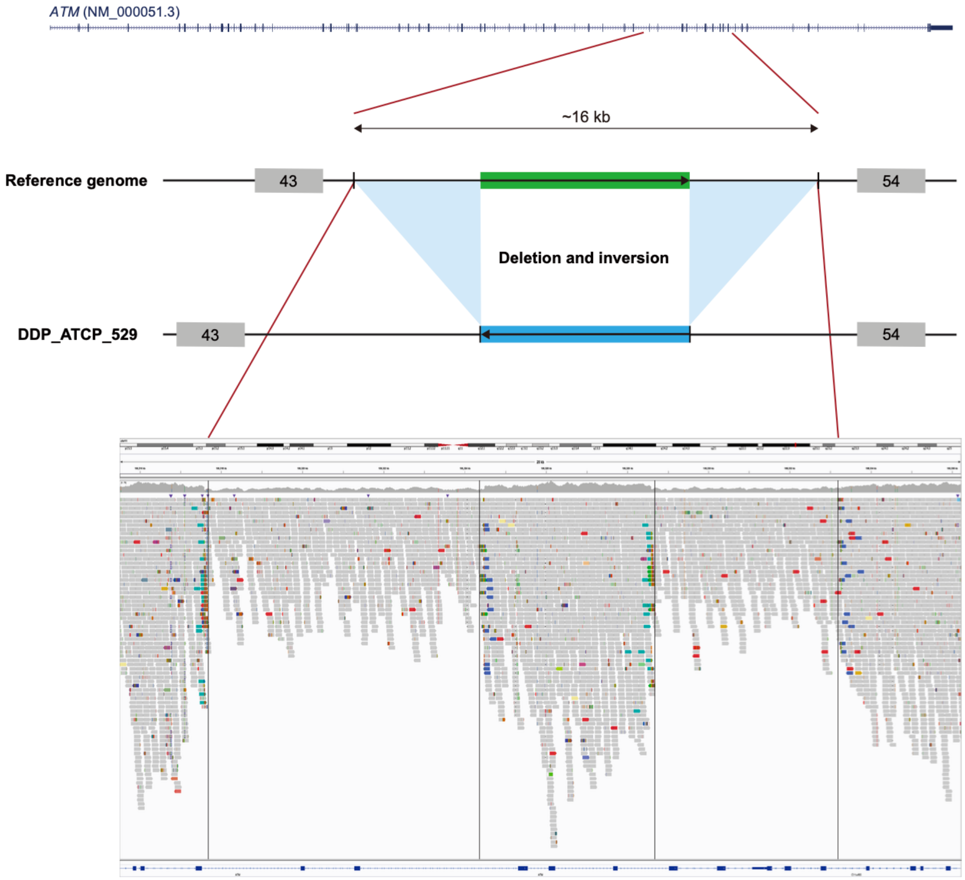

b Copy number profile of a complex SV in DDP_ATCP_529

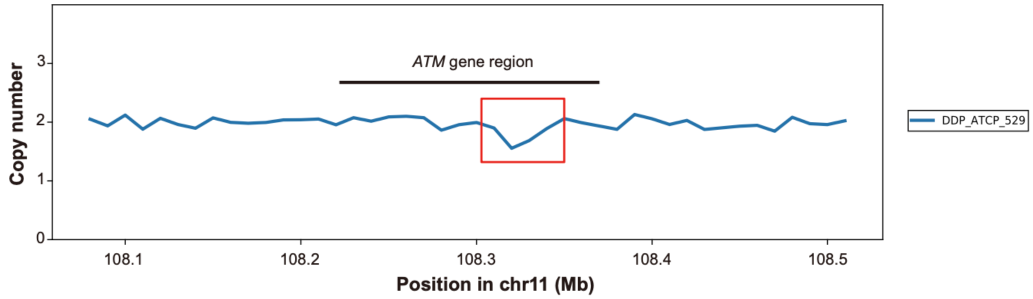

Supplementary Fig. 2. SVs - a complex CNV. (**a**) Schematic illustration and a screenshot of IGV of a complex CNV event in DDP_ATCP_529. This event is presumed to be a composite event of a dislocation of exon 44–53, followed by a terminal deletion of the dislocated fragment, followed by a reinsertion of the fragment back into the original locus. (**b**) Copy number profile of the *ATM* region (shown in **panel a**) in DDP_ATCP_529, showing only a subtle loss of the copy number due to the complex nature of the event.

Supplementary Fig. 3.

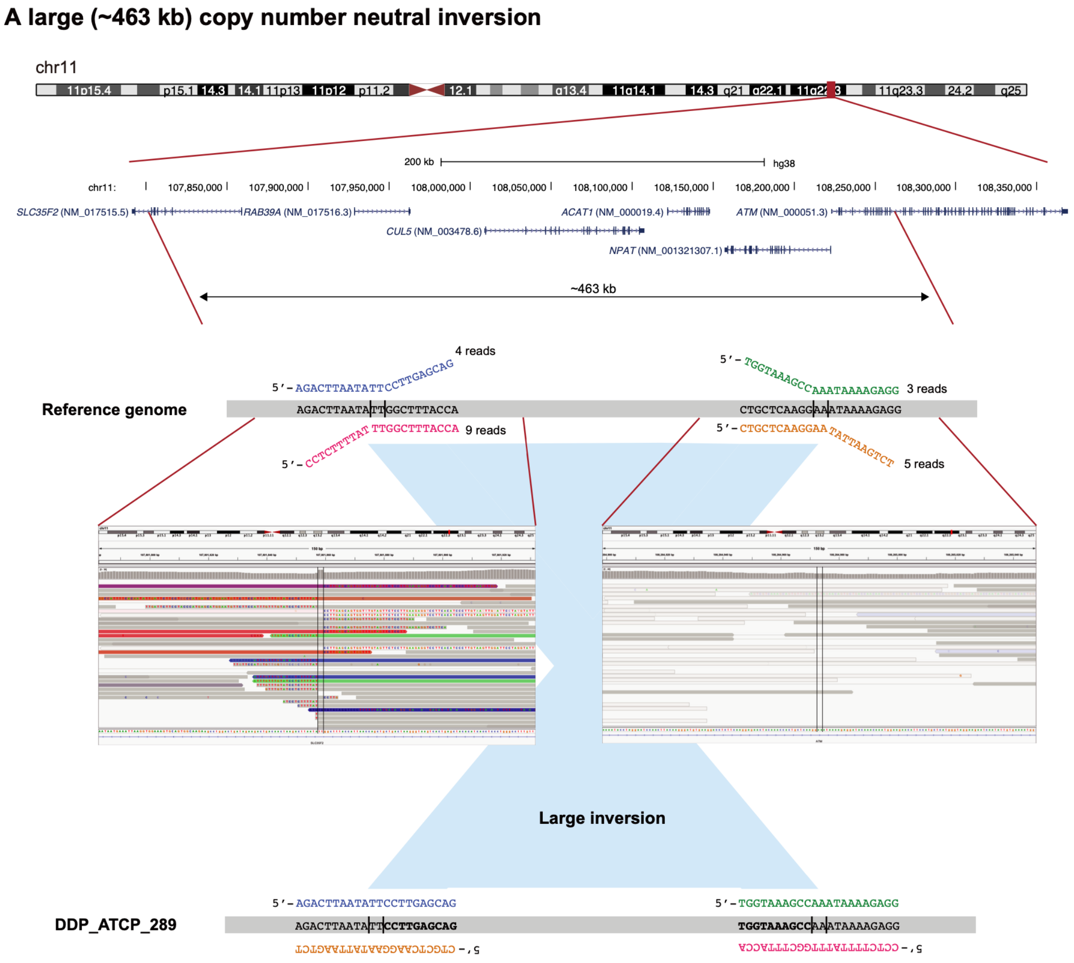

Supplementary Fig. 3. SVs - a copy number neutral inversion. Schematic illustration and a screenshot of IGV of a large (~463 kb) copy number neutral inversion event involving *SLC35F2* and *ATM* in DDP_ATCP_289.

Supplementary Fig. 4.

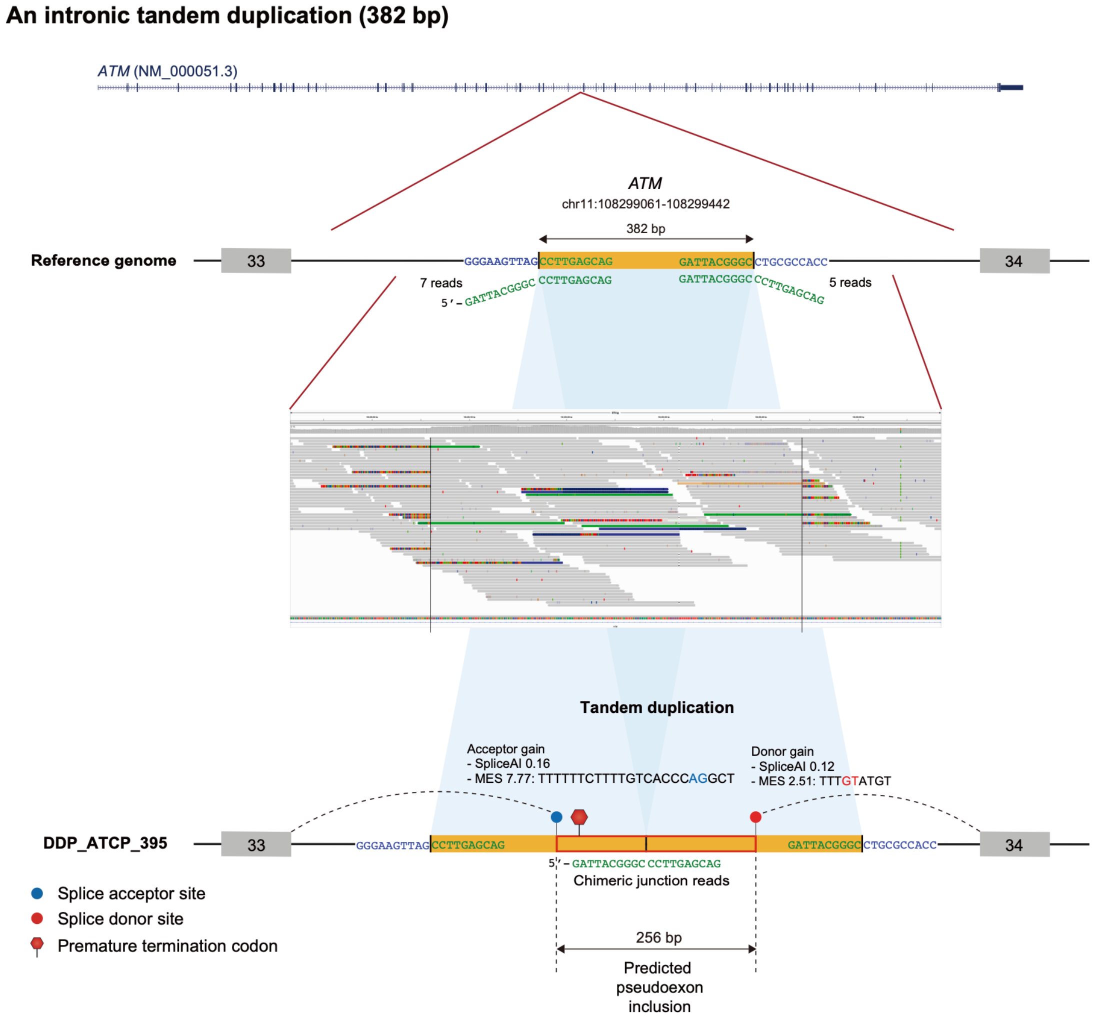

Supplementary Fig. 4. SVs - a tandem duplication. Schematic illustration and a screenshot of IGV of tandem duplication in *ATM* intron 33 in DDP_ATCP_395. One splice acceptor site and one splice donor site are predicted by SpliceAI to be activated by the tandem duplication, leading to a pseudoexon inclusion event. This event is classified as probably amenable to splice-switching ASO therapy. MES, MaxEntScan.

Supplementary Fig. 5.

Off-target profile of AT008

Genomic matches of AT008 subsequences

Length (nt)	Number of possible subsequences	Number of off-target hits	Off-target locus (hg38)	Gene annotation (RefSeq)		
				Gene (transcript ID)	Strand	Region (distance to juction)
22	1	0	-	-	-	-
21	2	0	-	-	-	-
20	3	0	-	-	-	-
19	4	0	-	-	-	-
18	5	1	chr9:20415802-20415819	*MLLT3 (NM_004529)*	+	intron (1376, 32303)
17	6	0	-	-	-	-
16	7	3	chr18:42751421-42751436	*RIT2 (NM_002930)*	+	intron (7700, 172135)
			chr2:149693939-149693954	*MMADHC-DT (NR_110240)*	-	intron (106088, 49763)
			chr1:12271823-12271838	*VPS13D (NM_015378)*	-	intron (698, 1164)

Genomic matches of AT008 sequence allowing internal mismatches

Length (nt)	Number of mismatches	Number of off-target hits	Off-target locus (hg38)	Gene annotation (RefSeq)		
				Gene (transcript ID)	Strand	Region (distance to juction)
22	1	0	-	-	-	-
22	2	0	-	-	-	-

Genomic matches of AT008 sequence allowing an internal 1 nt indel

Length (nt)	Number of insertion or deletion	Number of off-target hits	Off-target locus (hg38)	Gene annotation (RefSeq)		
				Gene (transcript ID)	Strand	Region (distance to juction)
23	1 (insertion)	0	-	-	-	-
21	1 (deletion)	0	-	-	-	-

Supplementary Fig. 5. Predicted off-targets of AT008. Variations of the AT008 sequence were generated. The sequences with variation include trimmed sequences until the length of the sequences became 16 nt, internally mismatched sequences by up to 2 nt, sequences with up to 1 nt deletion/insertion. The sequences were aligned to the reference human genome and transcriptome. The number and identity of the off-target matches are presented.

Supplementary Fig. 6.

Off-target profile of AT026

Genomic matches of AT026 subsequences

Length (nt)	Number of possible subsequences	Number of off-target hits	Off-target locus (hg38)	Gene annotation (RefSeq)		
				Gene (transcript ID)	Strand	Region (distance to juction)
21	1	0	-	-	-	-
20	2	0	-	-	-	-
19	3	0	-	-	-	-
18	4	1	chr9:20415802-20415819	*MLLT3 (NM_004529)*	+	intron (1376, 32303)
17	5	1	chr18:42751420-42751436	*RIT2 (NM_002930)*	+	intron (7699, 172135)
16	6	1	chr2:149693939-149693954	*MMADHC-DT (NR_110240)*	-	intron (106088, 49763)

Genomic matches of AT026 sequence allowing internal mismatches

Length (nt)	Number of mismatches	Number of off-target hits	Off-target locus (hg38)	Gene annotation (RefSeq)		
				Gene (transcript ID)	Strand	Region (distance to juction)
21	1	0	-	-	-	-
21	2	2	chr3:114830037-114830057	*ZBTB20 (NM_001348800)*	+	intron (28862, 70246)
			chr6:145938497-145938517	*SHPRH (NM_001042683)*	+	intron (3055, 2205)

Genomic matches of AT026 sequence allowing an internal 1 nt indel

Length (nt)	Number of insertion or deletion	Number of off-target hits	Off-target locus (hg38)	Gene annotation (RefSeq)		
				Gene (transcript ID)	Strand	Region (distance to juction)
22	1 (insertion)	0	-	-	-	-
20	1 (deletion)	0	-	-	-	-

Supplementary Fig. 6. Predicted off-targets of AT026. Same as in **Supplementary Fig. 5**, but for AT026.

Supplementary Fig. 7.

Off-target profile of nusinersen

Genomic matches of nusinersen subsequences

Length (nt)	Number of possible subsequences	Number of off-target hits	Off-target locus (hg38)	Gene annotation (RefSeq)		
				Gene (transcript ID)	Strand	Region (distance to juction)
18	1	0	-	-	-	-
17	2	2	chr15:32146950-32146965	*LOC100288637 (NR_038253)*	+	intron (457468, 274716)
			chr5_GL339449v2_alt:458772-458787	*GUSBP3 (NR_027386)*	+	intron (346621, 1812)
16	3	4	chr11:19571375-19571389	*NAV2 (NM_001111018)**	-	intron (220347, 261094)
			chr18:67894050-67894064	*LOC643542 (NR_033921)*	-	intron (19793, 3115)
			chr5_KI270897v1_alt:500437-500451	*GUSBP3 (NR_027386)*	-	intron (1806, 29253)
			chr8:40746847-40746861	*ZMAT4 (NM_024645)*	+	intron (49445, 20779)

Genomic matches of nusinersen sequence allowing internal mismatches

Length (nt)	Number of mismatches	Number of off-target hits	Off-target locus (hg38)	Gene annotation (RefSeq)		
				Gene (transcript ID)	Strand	Region (distance to juction)
18	1	1	chr12:112960443-112960460	*OAS3 (NM_006187)*	-	intron (9467, 610)

Genomic matches of nusinersen sequence allowing an internal 1 nt indel

Length (nt)	Number of insertion or deletion	Number of off-target hits	Off-target locus (hg38)	Gene annotation (RefSeq)		
				Gene (transcript ID)	Strand	Region (distance to juction)
19	1 (insertion)	3	chr11:113140403-113140419	*EFCAB6 (NM_022785)*	+	intron (367, 5039)
			chr20:61503412-61503430	*CDH4 (NM_001794)*	+	intron (248474, 240132)
			chr5_KI270897v1_alt:500436-500454	*GUSBP3 (NR_027386)*	-	intron (1805, 29250)
17	1 (deletion)	2	chr11:113140403-113140419	*NCAM1 (NM_181351)*	-	intron (178738, 61959)
			chr3:178704046-178704062	*KCNMB2-AS1 (NR_126560)*	+	intron (166649, 44341)

Supplementary Fig. 7. Predicted off-targets of nusinersen. Same as in **Supplementary Fig. 5**, but for nusinersen. Due to the large number of off-targets, mismatches were allowed by only 1 nt.

Supplementary Fig. 8.

Off-target profile of milasen

Genomic matches of milasen subsequences

Length (nt)	Number of possible subsequences	Number of off-target hits	Off-target locus (hg38)	Gene annotation (RefSeq)		
				Gene (transcript ID)	Strand	Region (distance to juction)
22	1	0	-	-	-	-
21	2	0	-	-	-	-
20	3	0	-	-	-	-
19	4	0	-	-	-	-
18	5	0	-	-	-	-
17	6	0	-	-	-	-
16	7	3	chr2:31196302-31196317	*CAPN14 (NM_001145122)*	+	intron (1818, 931)
			chr7:11518139-11518154	*THSD7A (NM_015204)*	+	intron (36156, 23264)
			chr5:32260228-32260243	*MTMR12 (NM_001040446)*	+	intron (4459, 2869)

Genomic matches of milasen sequence allowing internal mismatches

Length (nt)	Number of mismatches	Number of off-target hits	Off-target locus (hg38)	Gene annotation (RefSeq)		
				Gene (transcript ID)	Strand	Region (distance to juction)
22	1	0	-	-	-	-
22	2	0	-	-	-	-

Genomic matches of milasen sequence allowing an internal 1 nt indel

Length (nt)	Number of insertion or deletion	Number of off-target hits	Off-target locus (hg38)	Gene annotation (RefSeq)		
				Gene (transcript ID)	Strand	Region (distance to juction)
23	1 (insertion)	0	-	-	-	-
21	1 (deletion)	0	-	-	-	-

Supplementary Fig. 8. Predicted off-targets of milasen. Same as in **Supplementary Fig. 5**, but for milasen.

Supplementary Fig. 9.

In vitro toxicity assay for AT008 and AT056

a

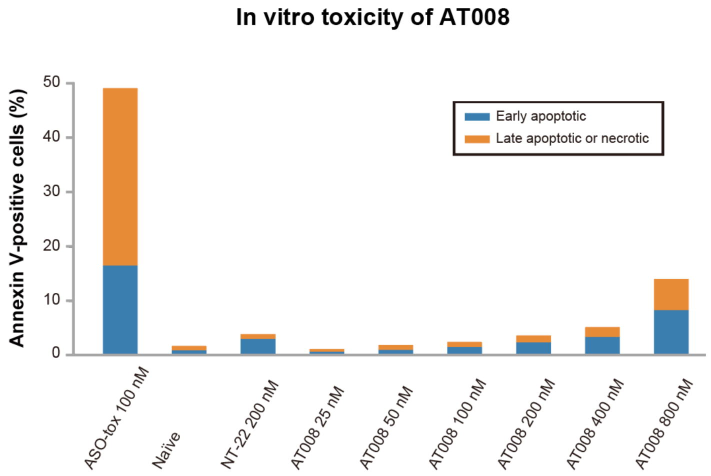

b

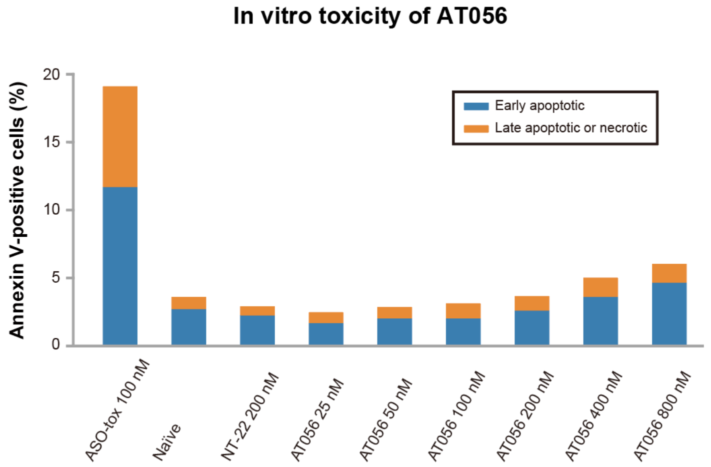

Supplementary Fig. 9. In vitro toxicity of AT008 and AT056. (**a**) Annexin V assay results for AT008 (targeting c.7865C>T). The Annexin V assay was conducted to confirm in vitro toxicity related to apoptosis. The fraction positive for Annexin V and negative for propidium iodide (PI) was classified as ‘early apoptotic’, while the fraction positive for both Annexin V and PI was classified as ‘late apoptotic or necrotic’. NT-22, non-targeting ASO; ASO-tox, an ASO known to be toxic (**Supplementary Table 13**). (**b**) Annexin V assay results for AT056 (targeting c.5763-1050A>G). For FACS sequential gating schemes, see **Supplementary Fig. 10**.

Supplementary Fig. 10.

FACS sequential gating schemes

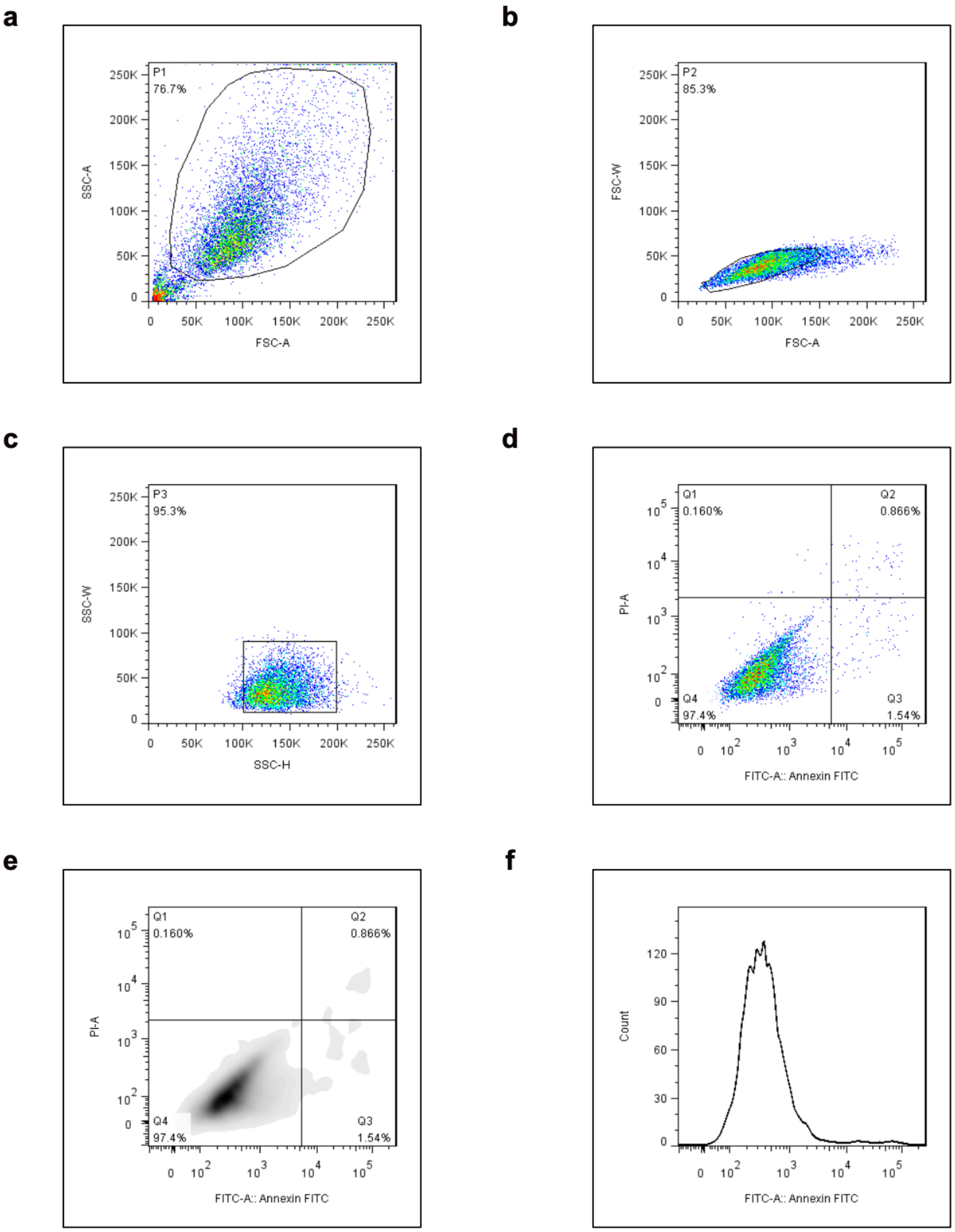

Supplementary Fig. 10. FACS sequential gating schemes. Representative illustrations of the FACS sequential gating schemes are shown using the AT008 100 nM sample (**Supplementary Fig. 9**). All plots were exported from FlowJo. (**a**) In the FSC-A vs. SSC-A plot, the first gate (P1) was set to discriminate the population containing small-sized debris from the live cell

population. (**b, c**) The second (P2) and third (P3) gates were set in the FSC-H vs. FSC-W and SSC-H vs. SSC-W plots, respectively, to remove doublets and clumps. (**d**) The quad gates were set to classify cells under different physiological and pathological states based on the FITC Annexin V and propidium iodide (PI) staining. The cells in the region Q4, Q3, and Q2 represented living cells, early apoptotic cells, and late apoptotic or necrotic cells, respectively. (**e**) The density plot with quad gates was set to classify cells under different physiological and pathological states based on the FITC Annexin V and PI staining, where Q4, Q3, and Q2 represent living, early apoptotic, and late apoptotic or necrotic cells, respectively. (**f**) The count plot of cells under different apoptotic states based on FITC Annexin V. Most of the cells were classified as living cells, with only a small fraction of the cells classified as early or late apoptotic or necrotic.

Supplementary Fig. 11.

Off-target profile of AT056

Genomic matches of AT056 subsequences

Length (nt)	Number of possible subsequences	Number of off-target hits	Off-target locus (hg38)	Gene annotation (RefSeq)		
				Gene (transcript ID)	Strand	Region (distance to juction)
22	1	0	-	-	-	-
21	2	0	-	-	-	-
20	3	0	-	-	-	-
19	4	0	-	-	-	-
18	5	0	-	-	-	-
17	6	0	-	-	-	-
16	7	6	chr4:145633846-145633861	*MMAA (NM_172250)*	-	intron (14438, 5213)
			chr7:88033323-88033338	*ADAM22 (NM_001324418)*	-	intron (54910, 42287)
			chr8:17397943-17397958	*MTMR7 (NM_004686)*	+	intron (24702, 15310)
			chr9:28235248-28235263	*LINGO2 (NM_001258282)*	+	intron (284541, 59944)
			chrX:75390576-75390591	*ZDHHC15 (NM_144969)*	+	intron (11377, 26495)
			chr1:99646962-99646977	*PALMD (NM_017734)*	-	intron (599, 15341)

Genomic matches of AT056 sequence allowing internal mismatches

Length (nt)	Number of mismatches	Number of off-target hits	Off-target locus (hg38)	Gene annotation (RefSeq)		
				Gene (transcript ID)	Strand	Region (distance to juction)
22	1	0	-	-	-	-
22	2	3	chr13:23261106-23261127	*SGCG (NM_000231)*	-	intron (10388, 18231)
			chrX:75390576-75390597	*ZDHHC15 (NM_144969)*	+	intron (11377, 26489)
			chr14:86051487-86051508	*LINC02328 (NR_110155)*	-	intron (116625, 19354)

Genomic matches of AT056 sequence allowing an internal 1 nt indel

Length (nt)	Number of insertion or deletion	Number of off-target hits	Off-target locus (hg38)	Gene annotation (RefSeq)		
				Gene (transcript ID)	Strand	Region (distance to juction)
23	1 (insertion)	0	-	-	-	-
21	1 (deletion)	0	-	-	-	-

Supplementary Fig. 11. Predicted off-targets of AT056. Same as in **Supplementary Fig. 5**, but for AT056.

Supplementary Fig. 12.

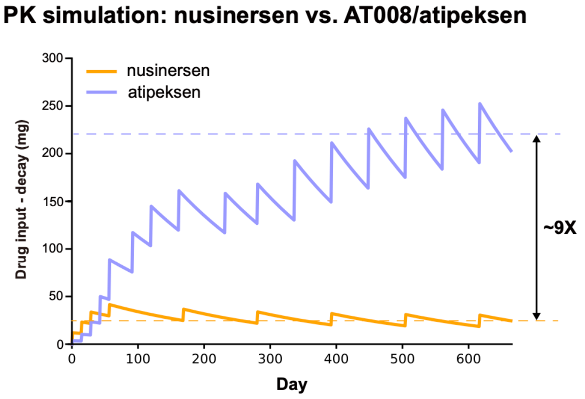

Supplementary Fig. 12. PK simulation - nusinersen vs. AT008/atipeksen.

SUPPLEMENTARY TABLES 1–16

Supplementary Table 1. Patient information.

Supplementary Table 2. GATK SNV and short indel calls.

Supplementary Table 3. Disease candidate SVs.

Supplementary Table 4. WGS variant calling validation - Sanger sequencing primers.

Supplementary Table 5. WGS variant calling validation - Sanger sequencing results.

Supplementary Table 5. WGS variant calling validation - Sanger sequencing results.

Patient ID	Variant 1	Variant 1 Sanger chromatogram	Variant 1 genotype	Variant 2	Variant 2 Sanger chromatogram	Variant 2 genotype
DDP_ATCP_139	NM_000051.3: c.8545C>T	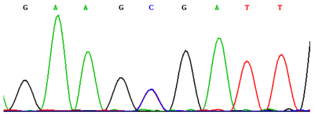	C>T Heterozygote	NM_000051.3: c.2251-10T>G	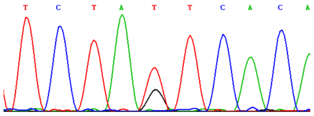	T>G Heterozygote
DDP_ATCP_42	NM_000051.3: c.3993+1G>A	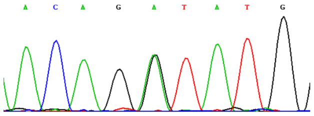	G>A Heterozygote	NM_000051.3: c.5763-1050A>G	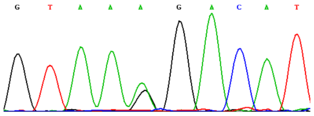	A>G Heterozygote
DDP_ATCP_38	NM_000051.3: c.1931C>A	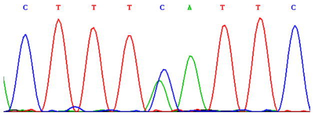	C>A Heterozygote	NM_000051.3: c.331+1G>A	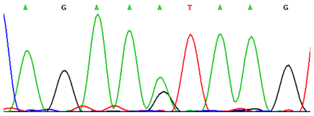	G>A Heterozygote
DDP_ATCP_39	NM_000051.3: c.1931C>A	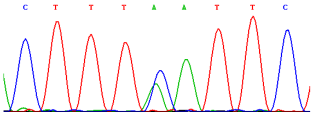	C>A Heterozygote	NM_000051.3: c.331+1G>A	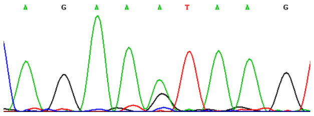	G>A Heterozygote
DDP_ATCP_96	NM_000051.3: c.8147T>C	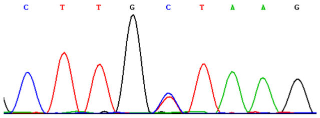	T>C Heterozygote	NM_000051.3: c.3529T>C	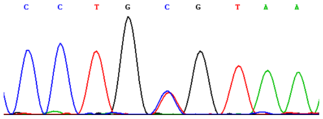	T>C Heterozygote
DDP_ATCP_151	CNV (exon 62-63 deletion)	Not available		NM_000051.3: c.8147T>C	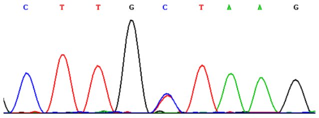	T>C Heterozygote
DDP_ATCP_152	CNV (exon 62-63 deletion)	Not available		NM_000051.3: c.8147T>C	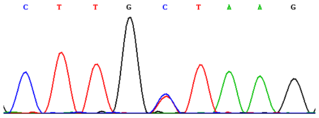	T>C Heterozygote
DDP_ATCP_218	NM_000051.3: c.8147T>C	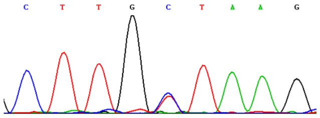	T>C Heterozygote	NM_000051.3: c.8494C>T	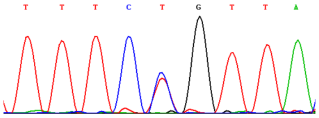	C>T Heterozygote
DDP_ATCP_34	NM_000051.3: c.1737G>A	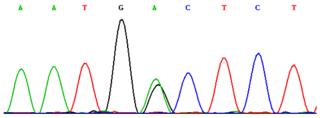	G>A Heterozygote	NM_000051.3: c.2839-579_2839-576del	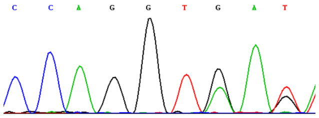	GGTAA>G Heterozygote

DDP_ATCP _231	NM_000051.3: c.170G>A	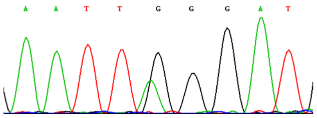	G>A Heterozygote	NM_000051.3: c.2839-581G>A	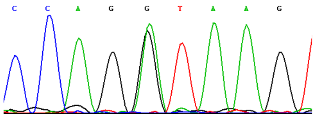	G>A Heterozygote
DDP_ATCP _138	NM_000051.3: c.4432C>T	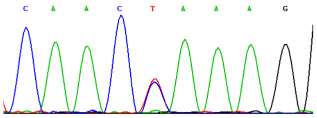	C>T Heterozygote	NM_000051.3: c.3489C>T	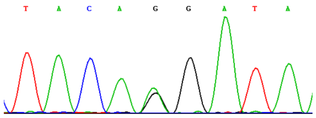	G>A (Reverse primer) Heterozygote
DDP_ATCP _302	NM_000051.3: c.1402_1403del	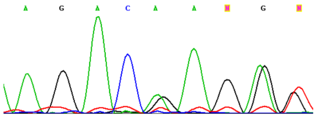	CAA>C Heterozygote	NM_000051.3: c.4801A>G	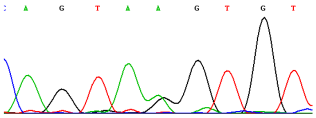	A>G Heterozygote
DDP_ATCP _265	NM_000051.3: c.3200del	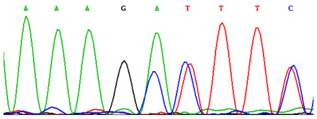	GA>G Heterozygote	NM_000051.3: c.6573-15T>G	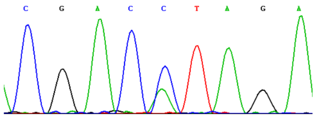	A>C (Reverse primer) Heterozygote
DDP_ATCP _124	NM_000051.3: c.1139_1142dup	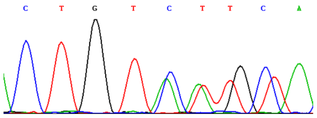	A>CTGTA (Reverse primer) Heterozygote	NM_000051.3: c.8080G>A	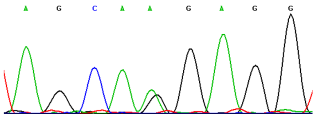	G>A Heterozygote
DDP_ATCP _124 (Variant 3)	NM_000051.3: c.6348-986G>T	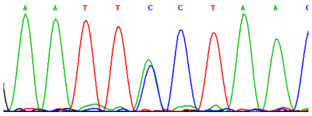	C>A (Reverse primer) Heterozygote			
DDP_ATCP _146	NM_000051.3: c.901G>A	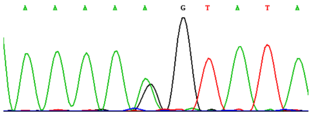	G>A Heterozygote	NM_000051.3: c.3994-159A>G	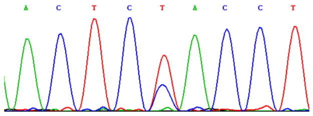	T>C (Reverse primer) Heterozygote
DDP_ATCP _344	NM_000051.3: c.4664del	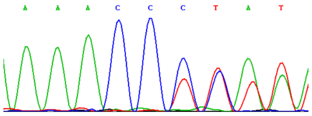	CT>C Heterozygote	NM_000051.3: c.2639-21A>G	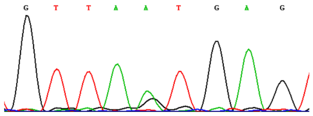	A>G Heterozygote

Supplementary Table 6. Phasing by trio Sanger sequencing.

Supplementary Table 6. Phasing by trio Sanger sequencing.

Individual	Variant 1	Variant 1 Sanger chromatogram	Variant 1 genotype	Variant 2	Variant 2 Sanger chromatogram	Variant 2 genotype
DDP_ATCP_42 Proband	NM_000051.3: c.3993+1G>A	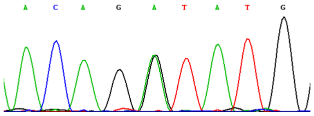	G>A Heterozygote (Maternal)	NM_000051.3: c.5763-1050A>G	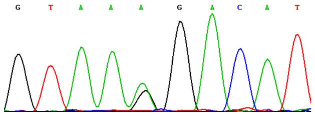	A>G Heterozygote (Paternal)
DDP_ATCP_42 Mother	NM_000051.3: c.3993+1G>A	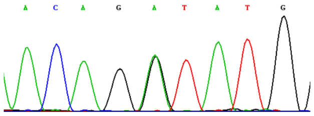	G>A Heterozygote		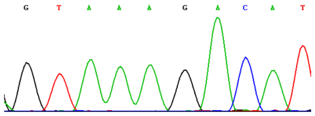	A (REF) Homozygote
DDP_ATCP_42 Father		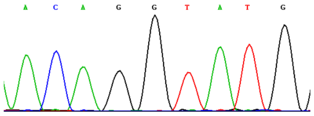	G (REF) Homozygote	NM_000051.3: c.5763-1050A>G	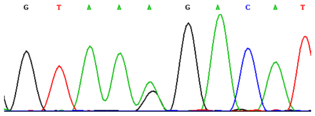	A>G Heterozygote
DDP_ATCP_38 Proband (sibling with 39)	NM_000051.3: c.1931C>A	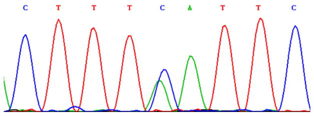	C>A Heterozygote (Paternal)	NM_000051.3: c.331+1G>A	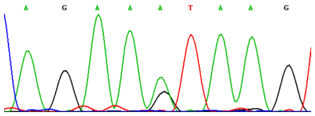	G>A Heterozygote (Maternal)
DDP_ATCP_39 Proband (sibling with 38)	NM_000051.3: c.1931C>A	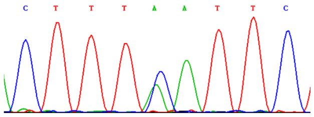	C>A Heterozygote (Paternal)		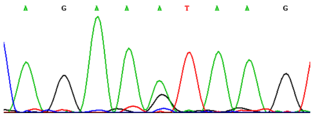	G>A Heterozygote (Maternal)
DDP_ATCP_38/39 Mother		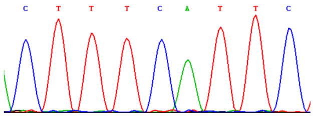	C (REF) Homozygote	NM_000051.3: c.331+1G>A	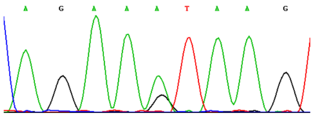	G>A Heterozygote
DDP_ATCP_38/39 Father	NM_000051.3: c.1931C>A	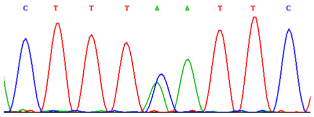	C>A Heterozygote		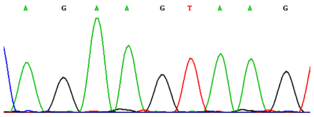	G (REF) Homozygote
DDP_ATCP_96 Proband	NM_000051.3: c.8147T>C	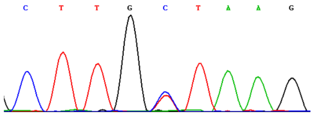	T>C Heterozygote (Paternal)	NM_000051.3: c.3529T>C	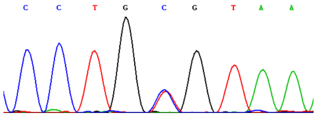	T>C Heterozygote (Maternal)
DDP_ATCP_96 Mother		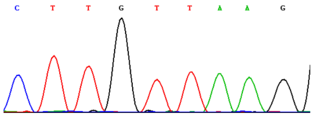	T (REF) Homozygote	NM_000051.3: c.3529T>C	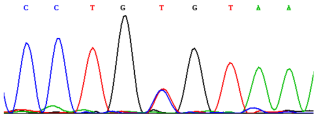	T>C Heterozygote
DDP_ATCP_96 Father	NM_000051.3: c.8147T>C	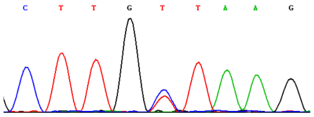	T>C Heterozygote		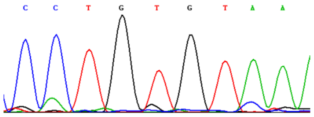	T (REF) Homozygote
DDP_ATCP_218 Proband	NM_000051.3: c.8147T>C	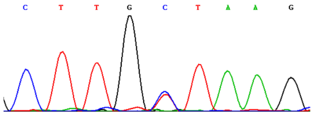	T>C Heterozygote (Paternal)	NM_000051.3: c.8494C>T	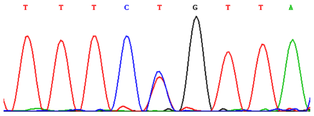	C>T Heterozygote (Maternal)
DDP_ATCP_218 Mother		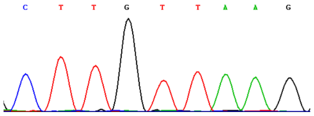	T (REF) Homozygote	NM_000051.3: c.8494C>T	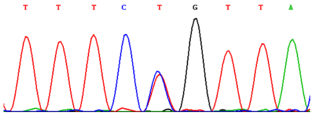	C>T Heterozygote

DDP_ATCP _218 Father	NM_000051.3: c.8147T>C	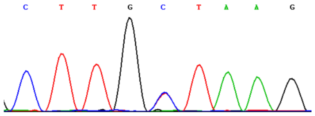	T>C Heterozygote		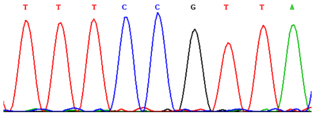	C (REF) Homozygote
DDP_ATCP _218 Sibling (unaffected)		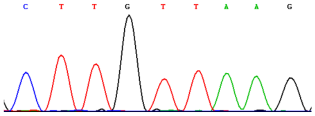	T (REF) Homozygote	NM_000051.3: c.8494C>T	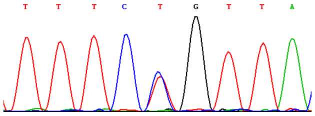	C>T Heterozygote (Maternal)
A-T child in expanded access trial Proband	NM_000051.3: c.8585-13_8598del	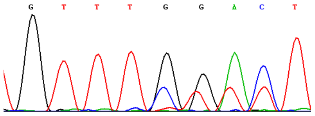	27 bp deletion Heterozygote (Paternal)	NM_000051.3: c.7865C>T	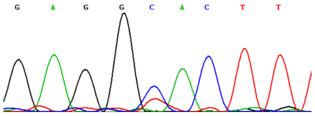	C>T Heterozygote (Maternal)
A-T child in expanded access trial Mother		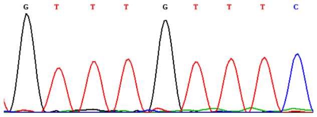	C (REF) Homozygote	NM_000051.3: c.7865C>T	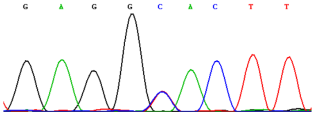	C>T Heterozygote
A-T child in expanded access trial Father	NM_000051.3: c.8585-13_8598del	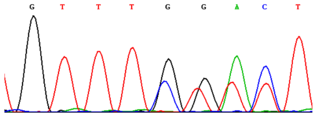	27 bp deletion Heterozygote		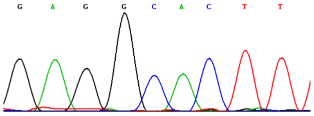	C (REF) Homozygote

Supplementary Table 7. **Disease candidate missense and in-frame deletion variants.**

Supplementary Table 8. Recurrence of disease candidate variants.

Supplementary Table 9. **ASO-amenable variants.**

Supplementary Table 10. **Minigene assay - primers.**

Supplementary Table 11. Minigene assay - Sanger sequencing genotype validation.

Supplementary Table 11. Minigene assay - Sanger sequencing genotype validation.

Patient ID	Availability of patient gDNA	Constructs with patient amplicon (PT) or mutagenesis kit (KIT) †	Variant	REF plasmid ID	REF Sanger chromatogram	ALT plasmid ID	ALT Sanger chromatogram
DDP_ ATCP_ 34	Yes	KIT	c.2839-579_2839-576del	REF.34. C1.1	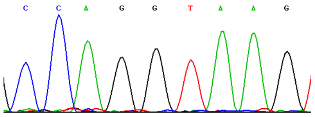	MUT.34. C1.2	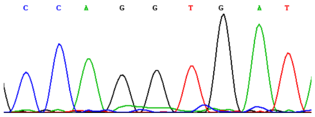
DDP_ ATCP_ 231	Yes	PT	c.2839-581G>A	REF.34. C1.1	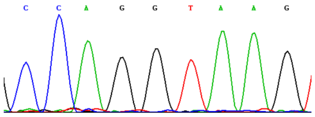	PT.231. C1.1	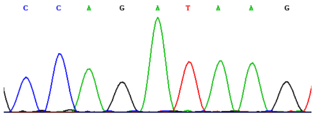
DDP_ ATCP_ 146	Yes	PT	c.3994-159A>G	PT.146. C3.2	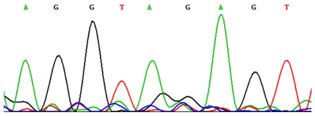	PT.146. C3.1	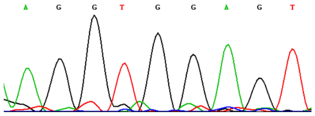
DDP_ ATCP_ 138	Yes	PT	c.3489C>T	PT.138. C2.5	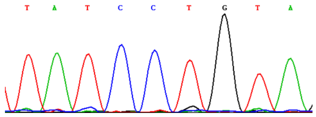	PT.138. C2.1	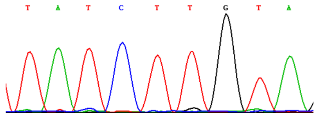
DDP_ ATCP_ 302	Yes	PT	c.4801A>G	PT.302. C2.1	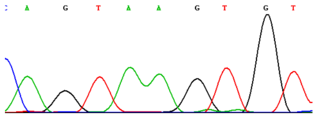	PT.302. C2.2	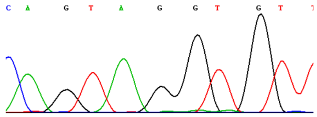
DDP_ ATCP_ 124	Yes	KIT	c.6348-986G>T	REF.124. C2.7	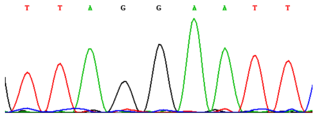	MUT.124 .C2.4	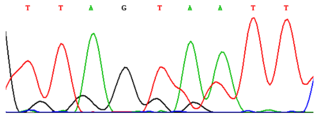
DDP_ ATCP_ 344	Yes	KIT	c.2639-21A>G	REF.34. C1.1	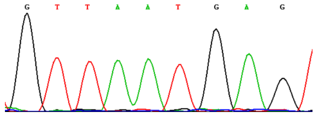	MUT.344 .C1.1	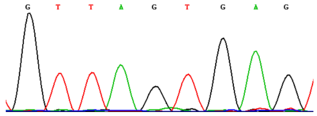
DDP_ ATCP_ 104/434	No	KIT	c.2639-22_2639-20del	REF.34. C1.1	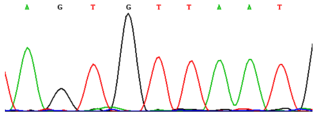	MUT.434 .C1.1	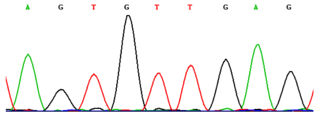
DDP_ ATCP_ 265	Yes	PT	c.6573-15T>G	PT.265. C2.1	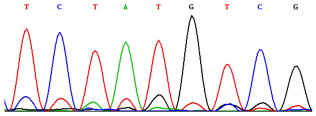	PT_265. C2.2	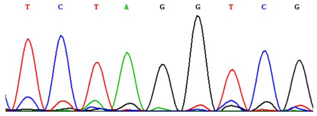
DDP_ ATCP_ 110	No	KIT	c.496+5G>A	REF.110. C2.2	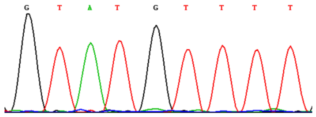	MUT.110 .C2.6	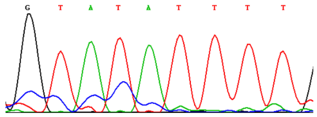
DDP_ ATCP_ 14/421/ 456/78/ 92	Yes	PT	c.2250G>A	MAN234 1.C1.5	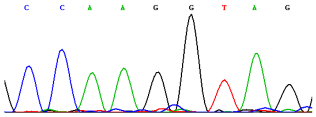	MAN234 1.C1.4	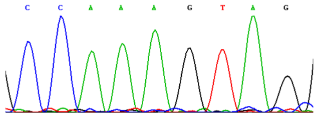

† Due to the technical challenge in long-range PCR with patient's saliva gDNA as template, *ATM* gene amplicons with several variants of interest could not be amplified from patient gDNA. These minigene plasmids were finally constructed via NEB Q5 site-directed mutagenesis kit.

Supplementary Table 12. **Minigene assay - Sanger sequencing mis-splicing validation.**

Supplementary Table 13. ASO sequences and chemistry.

Supplementary Table 14. Primers for ASO screening.

Supplementary Table 15. ASO-amenability of the *ABCA4* Nassisi cohort.

Supplementary Table 16. ASO-amenability of the *ABCA4* Del Pozo-Valero cohort.

REFERENCES

1. McConville, C. M. *et al.* Mutations associated with variant phenotypes in ataxia-telangiectasia. *Am J Hum Genet* **59**, 320–330 (1996).
2. Barone, G. *et al.* Modeling ATM mutant proteins from missense changes confirms retained kinase activity. *Hum Mutat* **30**, 1222–1230 (2009).
3. Abascal, F. *et al.* Expanded encyclopaedias of DNA elements in the human and mouse genomes. *Nature* **583**, 699–710 (2020).
4. Mitui, M. *et al.* Functional and computational assessment of missense variants in the ataxia-telangiectasia mutated (ATM) gene: mutations with increased cancer risk. *Hum Mutat* **30**, 12–21 (2009).
5. Sandoval, N. *et al.* Characterization of ATM Gene Mutations in 66 Ataxia Telangiectasia Families. *Hum Mol Genet* **8**, 69–79 (1999).
6. Mallott, J. *et al.* Newborn Screening for SCID Identifies Patients with Ataxia Telangiectasia. *J Clin Immunol* **33**, 540–549 (2013).
7. Micol, R. *et al.* Morbidity and mortality from ataxia-telangiectasia are associated with ATM genotype. *Journal of Allergy and Clinical Immunology* **128**, 382–390 (2011).
8. Canman, C. E. *et al.* Activation of the ATM kinase by ionizing radiation and phosphorylation of p53. *Science (1979)* **281**, 1677–1679 (1998).
9. Teraoka, S. N. *et al.* Splicing defects the ataxia-telangiectasia gene, ATM: Underlying mutations and consequences. *Am J Hum Genet* **64**, 1617–1631 (1999).
10. Du, L., Pollard, J. M. & Gatti, R. A. Correction of prototypic ATM splicing mutations and aberrant ATM function with antisense morpholino oligonucleotides. *Proceedings of the National Academy of Sciences* **104**, 6007–6012 (2007).
11. Verhagen, M. M. M. *et al.* Clinical spectrum of ataxia-telangiectasia in adulthood. *Neurology* **73**, 430–437 (2009).
12. Rothblum-Oviatt, C. *et al.* Ataxia telangiectasia: A review. *Orphanet J Rare Dis* **11**, 1–21 (2016).
13. Nassisi, M. *et al.* Prevalence of ABCA4 deep-intronic variants and related phenotype in an unsolved “one-hit” cohort with stargardt disease. *Int J Mol Sci* **20**, 1–19 (2019).
14. Del Pozo-Valero, M. *et al.* Genotype–Phenotype Correlations in a Spanish Cohort of 506 Families With Biallelic ABCA4 Pathogenic Variants. *Am J Ophthalmol* **219**, 195–204 (2020).
15. Iannaccone, S. T., Paul, D., Castro, D., Weprin, B. & Swift, D. Delivery of Nusinersen Through an Ommaya Reservoir in Spinal Muscular Atrophy. *J Clin Neuromuscul Dis* **22**, 129–134 (2021).
